# Supplementary material for: Interrogating a framework for diabetic retinopathy screening adherence: Qualitative insights from a severely affected and under-adherent population
Source: PLOS Glob Public Health. 2026 Apr 2;6(4):e0006230. doi: 10.1371/journal.pgph.0006230 (PMC13046135; doi:10.1371/journal.pgph.0006230)
Supplement: S1 Text — (PDF) [file pgph.0006230.s001.pdf]

Modeling the Decision-Making Process Underlying the Utilization of Screenings for Diabetic Retinopathy

Principal Investigator: Kristen Nwanyanwu MD, MBA, MHS

Interviewer: Joana Andoh, 4th year Medical Student

1

Preface

- Purpose: To ask about your experiences getting eye exams as a person with diabetes.
  - Your **honest** thoughts are invaluable.
  - You are the **expert** of your own experiences.
  - We may ask you to **elaborate.**
  - There are no right or wrong **answers.**
  - All your responses will be kept **anonymous.**

2

Resource Availability

- Insurance
- Money
- Transportation
- Homelessness
- Incarceration

- "I was in a tough place, you know. I was on the streets…after squinting for like two years to see everything I went back to an eye doctor finally."

3

Cues to Action

- Appointment reminders
- Annual exam schedule
- Prompting by primary care provider
- "Aha" moment

- "I went for an exam because the doctor that I had - she examined my eyes…she said I have to send you to the eye doctor…that's when I started getting these [eye] exams."

4

Knowledge-creating Experiences

- Family and friends with diabetes
- Education by provider
- Other resources

- "I mean nobody wants to be blind. I watched what my sister went through…when I think of her living in darkness for so long, you know, it's like I don't want to do that."

5

In-clinic Experiences

- Provider communication
- Trust and respect
- Customer service
- Overall experience

- "I asked [the provider] questions and they just…they never answered them. So I just basically – I tried finding answers on my own."

6

Vision Status

- Need for glasses
- Changes in vision
  - Blurry vision
  - Double vision
  - Loss of vision

- "It wasn't until I myself noticed a difference in my own eye sights that made me want to do [an eye exam]."

7

Competing Concerns

- Employment
- Childcare
- Health problems
- Substance Use

- "Well I wasn't keeping my appointments when I had the job…your employer don't give a doggone they just want you there."

8

Emotional Context

- Wanting to know more
- Fear of learning the severity
- Denial of current situation
- "Wake up call" event

- "I didn't think I needed it, and a lot of times when I go to the doctor all of a sudden I need stuff. And I didn't want that to happen, I wanted to think that my eyes were going to be OK."

9

Questions?

Thank you for participating!

10

Brief Post-Interview Survey

- Rate the visual quality of this interview
  - 1 2 3 4 5 6 7 8 9 10 NA
- Rate the audio quality of this interview
  - 1 2 3 4 5 6 7 8 9 10
- Any changes you would have made?

11
