# Supplementary material for: Interrogating a framework for diabetic retinopathy screening adherence: Qualitative insights from a severely affected and under-adherent population
Source: PLOS Glob Public Health. 2026 Apr 2;6(4):e0006230. doi: 10.1371/journal.pgph.0006230 (PMC13046135; doi:10.1371/journal.pgph.0006230)
Supplement: S2 Text — (DOCX) [file pgph.0006230.s002.docx]

# Study Title: Modeling the Decision-Making Process Underlying the Utilization of Screenings for Diabetic Retinopathy

Competing Concerns

Knowledge-creating Experiences

Cues to Action

Vision Status

Coding Density

Interview with participant EN9001 on March 9th, 2021.

--------------Start of Interview--------------------

Interviewer:

Okay. Perfect. All right, I think we're ready to go. Next. So the purpose of this study, as I mentioned before, is asked about your experiences getting eye exams. As a person with diabetes, we want to know what you think there are no right or wrong answers, and you are the expert of your own experiences.

We really want to know the details of your thinking. So I might ask you to elaborate on your answers more, even though your answers are clear, these interviews are missed, and they will not be shared with your doctor. Please let me know if I can rephrase any questions to make them clear. Okay. So this first theme that we're talking about is resources. In the past patients that we've talked to have talked about issues with insurance issues with payment, even transportation, getting to their eye exam, not having a home or even being incarcerated, that preventing them from getting their eye exam. A quote that we that I put on the screen is I was in a tough place, you know, I was on the streets after squinting for like two years to see everything. I went back to an eye doctor finally.

So with this, I want to ask you, what have been some challenges for you in getting eyes exams? EN9001

None of those.

Resource Availability

You know, so I mean, we know that we are but we pay no attention to what it is. You know, I don't want to keep running on with conversations, because I don't know how many different questions you'll have. You know, about me and 30 years of being a diabetic. You know, I do know, when I started out being a diabetic, I was insulin dependent from day one, I didn't have a pancreas, and I followed all the rules.

a lot of us, especially black folks, but you know, we tend to not accept being a diabetic.

Umm ignoranance, that that would be the best words that I could capture out of all that, because I think

Emotional Context

You know how to eat, you know, I was eating lamb doing very little of this and very little of that. And I grew out of it. I literally grout a bit, you know, I've been started to just be carefree. You know, just doing all the things everybody else is doing, you know, eating ice cream, you know, drinking soda, and all that stuff. And so it was a point that, you know, with me, my sugar ran between anywhere from three to 500 every day. Which is high and but I was able to control and stabilize myself high like that. Yeah. So come on.

Interviewer:

No, thank you. So, you know, you talked about ignorance. And sometimes is I'm curious to know whether you think it was knowledge you knew and were just not acting on it? Or was or do you feel like it was that you were kind of in this blissful like, You didn't know? Does that make sense?

EN9001

In-clinic Experiences

It makes sense. But you have to hear me ignorance is ignorance. I didn't know. When I got diagnosed. I was in the hospital for two weeks. And they were trying to regulate me for two weeks. And when I got out the hospital, I know I'm gonna be honest, which I think my doctor I was in [redacted] at the time. I

question.

ask him a question while we were in the hospital and the next day he will come back and answer the

I would

think my doctor was a bum. I'm going to say that. I don't think he knew much about diabetes.

What Besides that, when I got out the hospital, he was giving me so much insulin that I will

go outside and cut the grass and become so fatigued that would have to come in and go to sleep. You know, so, of course, me being new to this, I don't know really what's going on, you know, um, I would get the insulin I'm called them drops, where, you know, I get the shaking and stuff like that, because my sugar drops so low. Yeah. And that's how I started coming about carrying my sugars, three, four or 500.

Resource Availability

Competing Concerns

Vision Status

In-clinic Experiences

Coding Density

Interviewer:

Right, because you get it?

EN9001

Right, you know, that's when you go through that it's hurting. You know, it's exhausting and stuff. And so, I'm being a new diabetic, I don't really, you know, I didn't like it by that. So I started staying high, you know, I couldn't go outside and play basketball, because I don't have a pancreas. You know, when I drop I drop, you know, so I could be out there playing basketball or just playing tag with you, and it can drop suddenly. And there I go, you know, so I figured out then three to 500 it don't drop on me.

Right? And don't drop it off. So I got comfortable with that. And the reason I still say ignorance is because me myself, I went to several doctors after that, trying to figure out what diabetes was, and why do I have it? And how the heck did I get it?

You know, and you know, so one doctor wind up telling

Knowledge-creating Experiences

It can come out at any time from a sudden fall, you know, you can be on a ladder and fall off of it. And, you know, it can jump out into your system and bam, day you go, you know, so that's kinda I think what happened with me, I was in a traffic accident. And I wasn't the driver or anything, I was fine. You know, I was in the accident. And a week and after that, you know, I just started, it was summertime, I started eating watermelons and drinking constantly. I just couldn't stop, you know. And I went to the doctors, and emergency room, they said, I had too much insulin my system and take a laxative. I did that. Several hours, I'm right back throwing up and stuff, you know, feeling sick.

when they said, hey, you're a diabetic. And that's when they kept me.

And I went back to the same hospital emergency room. And that's

me that it's genetic. It's something inside your system.

Cues to Action

Interviewer:

I see. So that was kind of like your start of that journey in terms of trying to balance diabetes. But you mentioned when your sugars were high, you didn't have the shakes? in that. Yeah, well, much better, which makes sense. You know, you do what feels good.

EN9001

Yeah. Yeah.

Interviewer:

So I'm curious to know, then, from kind of making sure your sugars are high, what was the transition to then getting eye exams? Because you mentioned that you went to doctors, and they told you about diabetes? Were you told that diabetes can affect your vision?

EN9001

So, again, that's where the ignorance comes in. And that was part of the first paragraph that I said to

Emotional Context

Right? The understand. Everybody knows, I can go with you right now into a group. And people can be diabetic or not diabetic. And they all know that. You're gonna lose your sight. You can use your limbs, you know, you can lose your kidneys, etc. Right.
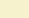


But

you, we hear it. But we don't accept it.

You know, it's like you told us, but we ain't seen no evidence of it, you know, so we still want to do what we normally do. Right? You know, so, basically, that's the same track. That's the same thing with me. You know, like I said, I just got off of

we don't pay attention until things start turning bad.

the diet and stuff from being a diabetic.

taking pills at first, and she wasn't eating anything with sugar in it at all. And you know, I'll be over a

And my sister's a diabetic. I used to go over her house. She was

Resource Availability

Competing Concerns

Knowledge-creating Experiences

Vision Status

Emotional Context

In-clinic Experiences

Coding Density

Yes, she went Taking no sugars at all, nothing, you know, I was, but I'm on the needle, you know. And and so, you know, you're seeing it happening to people and you're talking about it, but I will still good for me. You know, I didn't have any Hold on a second.

house. She's older than me. And she wind up still getting on the needle.

Interviewer:

No worries. Take your time.

EN9001

Yeah, that's the phone and saying I need to charge it. Oh, [redacted], bring me the phone cord charger, please.

Interviewer:

We're back. Okay, so I want to let you know that, you know, in you discussing your experiences, you're already answering some of the questions that I have lined up. So thank you for elaborating so much. I want to know. So you mentioned how, you know you get the information. But there's I'm hearing denial almost. But you don't necessarily apply it to you because you feel fine. Drew in, in that one of our next thing is like a cue, a cue to action. And I think you mentioned that in terms of saying that there was an event or something that made you realize this, it's more serious. Well you mentioned...

EN9001

Ah, I'm trying to plug this in one second. Well, remember I told you I was in [redacted]? Yeah, yep. And [redacted], I was kind of running free. I was hospitalized [redacted], in [redacted] a few times, you know, because I guess my sugar was too high and excetera. You know, so I was hospitalized a few times. And again, I get back out the hospital. And I go back to eating all kinds of crazy foods that everybody else is eating. Because that was just, I guess my state of mind at the time. I moved up here to [redacted] in about 2001. And I started getting my right,

when you put it in that machine. And ah, it's like that vision machine where they take pictures. Know,

I sought out eye doctor. And so they were giving me Give me the help for that. When they do your eyes

I started to twitch a lot. Okay. And that's when

Cues to Action

Interviewer:

where they were zapping the blood out your eyes.

Oh, the laser?

EN9001

Yeah, I was getting laser surgery. You know, so I was doing that for a while. Um,

Interviewer:

that was before your eye was twitching or

EN9001

that's when it first started twitching.

Interviewer: Okay.

EN9001

Interviewer:

started just going there. And, and that's the beginning of it.

And I went to an eye doctor, and that's when, you know, he told me I needed laser surgery. And so I

Resource Availability

Competing Concerns

Knowledge-creating Experiences

Cues to Action

Vision Status

Coding Density

I see. I see. So it's actually a twitching problem that made you go to the eye doctor and realize it was also there was also something related to your diabetes that you needed for your arm. Yes. Okay. Yeah, that makes sense. Okay. Um, and, again, related to this theme, is there anyone or anything that has helped you to kind of stay on top of your eye exam? You mentioned you went to one about two weeks ago in Interviewer:nuary. So it seems like you've been consistent and good with that. I'm curious to know what what has helped you on that path?

EN9001

Let me ask my wife a question. Hey, [redacted]. Do you remember the doctor's name went out? I first started going getting them. The eye surgery. You don't remember his name but oh. Remember we stopped going to him? Yes, [redacted]. Yeah. Well, you don't have to. Doctor he's downtown Los. So he starts with an L. I was going to him and to be honest, which I didn't like you probably didn't you know,

every time I went he was giving me laser surgery. Since like, when I left him my I was worse

In-clinic Experiences

You know, yeah. And and so to be honest with you now, there's some pills that I take. They're called Juice Plus their vitamins. A friend of mine who has cancer, someone talked him into taking them. And it's helped him with his blood cells and stuff. And he's been on me about it. So I, my eyes were like a che with sharks and jumping and stuff, you know. And so I started taking that, and that would stabilize my eyes from jumping. But then, you know, the blurred vision in this set

of shark coming in? And I don't know who suggested it.

had one of the best purchase attitudes I have ever, ever, ever run across, you know, she talked to me,

of the cheeriest. So yeah, his name is [redacted]. Okay, that was his name. When I was first going. She

getting in an uproar. Right. And, and in comes, this lady who's a doctor, and she has one. She had one

just not really nice and kind. They wouldn't explain them to it to me properly and acceptor. And I was

Now I'm just being on Chanel. Okay. Yeah, when I first got there, the doctors at Yale the first day was

I went to [redacted]. And there [redacted].

than it was when I went in

Interviewer:

you know, of to the kindness and the cetera.

eyes looked at, you know, I don't get to see her all the time, you know, but she was in initial impact,

what's really got me faithful. And I'm being switcheroo faithful to going to [redacted], and getting my

on the computer, this is what swollen, and this is what we need to bring down. You know, and so that's

And she talked to me and went through what we need to do how we need to do it. She pulls things up

like I was a little boy. And seriously, and she just she, you know, she, I got relaxed, like calm down.

Emotional Context

That's good to hear. So is that positive experience that also motivates you to keep going?

EN9001

Yes, yeah. Yeah. But you know,

you felt the someone cared.

Interviewer:

Yeah.

EN9001

No, no. And, and, you know, she's given me a needle on my a couple of times, you know, and then there's other colleagues, that's given me a needle in my eye also.

rarely miss an appointment. I rarely miss one.

you follow me? Yeah. You know, that's, that's what bored? at home, and this got me going. And I

But the home base, someone cared,

Interviewer:

Resource Availability

Knowledge-creating Experiences

Cues to Action

Vision Status

Emotional Context

In-clinic Experiences

Coding Density

Mm hmm. And when you do, do you find that it's because you forget, or particular reason? EN9001

out, you know, and so I'm in the car, and he's throwing faces and punches. So I need to go home, you

parent. And, you know, it has been a time that I'm on my way and one of my kids artistic and he act

No, like I said, I, you know, maybe because of the kids or something like that, you know, I'm a foster

Competing Concerns

Interviewer:

know, so yeah,

well, I thank you for that responsibility, especially taking care of foster kids. That's amazing.

EN9001

Yeah, well, we like to do that. We've been doing that for a while now. Wow. When?

Interviewer:

Do you find that that's been harder? Or I guess, has it been harder in the setting the pandemics since kids aren't always in school? And sometimes there's kind of...

EN9001

Well, here you know, I tell my wife, and I just talked to one of their advisors today. I said, if I was in my 30s, or 40s, I wouldn't really be doing this. Because you don't have the mental time. You have the physical? Yeah, for 30 or 40 years. You're still young and stuff, you know, but you don't have the mental time. You know, we're like grandparents, me and my wife. Yeah. So we now have the mental time. You know, I small story quickly.

Interviewer:

Yeah, of course.

EN9001

3040 years ago, I have a dog a pitbull. And crazy about my dog and stuff, you know, and I thought that I did pretty good care for my dog. But I'm now 40 years Later with another pitfall we have to have, right. But these dogs, we give them treats. You know, we spend time hugging them and stuff, you know, they get in the bed and play with us and acceptor these things I didn't do before with the other job. I didn't know it until now, you know, because I think back and saying well, I am never gave my dog a treat what was a treat? You know? Yeah, and nowadays, we spend more money on treats than we do on dog food generously, you know, they, when we leave the house, we give them a treat. When we come back in the house, we give them a treat. Every night before bedtime, we give them a treat, you know, for say all this comes along with just getting that older age. And so that's what I mean about the kids also, because we're this age, you know, the pandemic was rough, but we kind of had no problem with it. We had the kids for a year straight, you know, a year straight without them going to school at all, you know, because we were scared of getting the the virus ourselves because of our age and diabetes and all that stuff. So we kept them home. So you know, lindora they're back in school now.

Interviewer:

And I love it. Oh, that's amazing. Again, you're answering my questions as we go. So I kind of jumped a couple slides. But you mentioned like your positive experiences in the clinic, the ones where you felt like people cared, and that's kept you going.

I want to know, what has, I guess what could have made your experience better? Thinking about some of your past visits?

Resource Availability

Competing Concerns

Knowledge-creating Experiences

Cues to Action

Vision Status

Coding Density

EN9001

know that there is a chance you know, you know, we can get better? Because there's some days she'll

You know, because by her care, and showing she cared, made me honestly start caring about me. You

sitting down and giving me respect. And really showing that she cared was the best that it could be.

unquote, better. I'd say I can't get I can't get any better than what the doctor did. By by sitting down. by

But I don't know if anything could have made it quote

Emotional Context

In-clinic Experiences

I don't really I mean, I know what you're asking.

Interviewer:

know, and you felt she's caring about you. You're damn fool not to care about yourself.

pinch, you know, you know, and so she, you know, she made it just so comfortable and relaxing. You

know, cuz I hate needles. Oh, don't worry about it. You know, it ain't gonna hurt, you know, just a

the needle in your eye. That's a different thing they call the name, you know, I get to looking at it, you

tell you I've been there. And she look at the computer. Well, you know, we need to do laser surgery and

I like how you phrased that to how it almost, you know, makes you care about yourself more that she cared? Yeah, that's nice to hear.

EN9001

And you'll go through your troubles rather than go because, you know, they don't even care. You know,

husband say you got an appointment tomorrow, but I don't even feel like I'm not going you know, yeah.

know, it kind of tends it. You don't even want to go back. Right? You know what I mean? Your wife or

Yeah. Because when you go places and you find people with not nice personality, or attitude, you

Interviewer:

than than what she did.

so that's why I don't think that you know, anything could have they could have done anything better

I thank you for that answer, because that gives a great picture as to what you mean. You already mentioned your vision and kind of changes in your vision that made you go to the eye doctor. I want to know as a now how do you feel that your vision is compared to say 30 years ago when you were diagnosed?

EN9001

Thank you I'm back. I get cold on my chest sometimes. We're trying to figure out why. You know, although I took missing x and all of that stuff. Yeah, I still have phlegm in my chest. And I don't know why it's been going on for more than a year. Oh, no, no, I don't think it's the COVID thing.

Interviewer:

Have you been vaccinated?

EN9001

I just got vaccinated a week ago.

Interviewer:

Oh, yay, congratulations EN9001

Yeah. And I go, we go back in what two weeks now we go back? No, we both gathered together to vent to them. So I give you something that no one ever paid attention to. And I've told people about it.

I told the doctor about it in [redacted], et cetera and of course they paid no attention to it. Once they

that I couldn't see with my glasses that without my glasses I have perfect vision.

weeks, and they had me on saline machine. And I was on that for two weeks. And it got to the point

up but not far away. When I went to the hospital with diabetes, like I told you I stayed in there for two

Ever since my teenage, my younger years I've always worn glasses. Can't see far away. Can see close

Resource Availability

Competing Concerns

Knowledge-creating Experiences

Cues to Action

Vision Status

Emotional Context

In-clinic Experiences

Coding Density

Interviewer:

I've always wear glasses. So my vision is always been pretty bad.

vision.

it's something in that stuff that did something inside my system. But without it I went back to my port

took me off that machine from giving me that, two weeks later my vision went back to poor again. So

And by poor did you mean blurry or you couldn't see as far as you used to?

Participant:

No I used to have something like 20/100 something like that. So i'm wearing glasses and that kind of thick but it's always been like that.

In my younger days with diabetes it didn't change my vision at all right. My vision was the same.

been getting my eyes worked on. I watch TV and stuff with no glasses.

because I wasn't getting enough oxygen or what happened. But now what the strangest thing, since I've

I think I was starting to just lose my vision and that might have been why you know it was jumping

else. So it was all associated with diabetes.

somehow that was all connected because I hadn't hurt myself. I wasn't punched in the eye or nothing

that you know back there was swollen. He was telling me that I had blood in my eye and et cetera. So

I think the eye twitching was all associated with diabetes because when I went there he was telling me

Interviewer:

doesn't make much sense but...

that just to me it

do that so we just going out spending money on classes and my vision is and right yet

of settled. Because every time I go she looks at him and you know we need to do this and we need to

new glasses in about 2 years because I've been waiting for the doctor to say you know my eyes are kind

At night time I will put them on because it just makes me see a little clearer. But I haven't gotten any

glasses or anything. I'm in the house I'm watching TV I don't need glasses. I even drive with no glasses.

worn glasses my whole entire life 24/7. I would only take my glasses off to sleep. Now I can't eat with

If you notice me and you are talking now I don't know if you can see me I don't have glasses. I have

What I'm hearing is that you've been not wearing glasses 'cause you feel that you don't need them or you don't wanna wear them?

Participant:

I don't really need them. I see pretty well.

Interviewer:

Resource Availability

Competing Concerns

Knowledge-creating Experiences

Cues to Action

Vision Status

In-clinic Experiences

Coding Density

OK that's good to hear.

Participant:

It's not because I don't want them. Because like I said you know as far back it sixth grade I've been wearing glasses. I had to, I couldn't see.

Interviewer:

OK then it seems like at this point your vision is pretty stable.

Participant:

Yes. To me, yes.

Interviewer:

Are you retired as of now or do you work part-time?

Partcipiant Retired

Interviewer

So right now you're retired but in the past has your job or work schedule ever prevented you from also getting to your appointments.

Participant:

No. The last job that I had, I was working midnights.

Interviewer:

Another theme is emotional context. And I think you talked about this well in terms of describing your experience in the clinic and just feeling that people cared made you also want to care about keeping up with those appointments. I just wanted to reiterate that 'cause I think you you express that point well.

Do you feel like there were any other emotional experiences you've had in the eye clinic. Whether that was you mentioned denial whether that was fear, worry?

Partipicant:

No, but see you gotta remember one thing.

folks. Around black folks we don't like anybody messing in our eyes.

together. I'm not prejudice or nothing but I'm not in the circles you know of Spanish people or white

When you're talking about people. now I can only a lot of times speak for black folks because we talk

Fear is fear. The biggest fear is actually fear.

Emotional Context

***End of formal interview***

But we're talking about the eyes you know that's that's a real drawback for a lot of us.

to get one they cringe. Because if they're gonna look at your feet or something then it's we cool we OK.

friends and set of church friends and I tell them about I got a needle my eye and I am going next week

That's just the fear itself. That's the main thing. Even today when you know I talked to some of my

# Study Title: Modeling the Decision-Making Process Underlying the Utilization of Screenings for Diabetic Retinopathy

Knowledge-creating Experiences

In-clinic Experiences

Cues to Action

Vision Status

Emotional Context

Coding Density

Interview with participant EN9002 on April 1st, 2021. Interviewer:

So again, I mentioned a lot of this already, but I just want to reiterate, or just to repeat the purpose to ask the purpose of the studies to ask about your experiences getting a exam as a person with diabetes. Your honest thoughts are very valuable. You are the expert of your own experiences. We may ask you To elaborate that doesn't mean I don't think you gave a detailed response. I just want to hear more want to hear more what you're saying there are no right or wrong answers. And as I mentioned, for all your responses will be anonymous. Thank you so much. So the first theme, the so what I'm going to be showing you are themes that through previous conversations with people with diabetes, we found these themes where we found that oftentimes people had these issues when trying to get eye exam. So in going through the slide, I want to ask if you've ever encountered these issues, or if anything similar to this has happened to you. So this first theme is called resource availability. And this is a quote on the right, and it's from a patient who said I was in a tough place, you know, I was on the streets squinting, after squinting for like, two years, I see everything to see everything, I went back to an eye doctor finally. So some examples of this could be issues with insurance or issues with money paying for an eye exam, transportation getting to and from the hospital, homelessness or not having secure housing, or even being incarcerated, and not being able to see your doctor. So I guess given this first theme, is there anything that kind of speaks to you or anything that seems familiar? In regards to getting an exam, okay. Has there? I guess what resources as what has prevented you to get eye exam? If you can remember?

EN9002

Nothing early, I've always had insurance to get an eye exam. Okay.

Resource Availability

Interviewer:

Has there ever been a time where you scheduled an eye exam, but something came up or something that prevented you from being able to make you

EN9002

A couple years ago, I had an eye exam with Dr. [redacted] a will, but I ended up moving to [redacted].

Interviewer:

Okay. Was that a planned move or?

Unknown Speaker Yes.

Interviewer:

Okay, I see. So kind of moving between places. I see. And then you ended up coming back to [redacted]. So you're able to unfortunately, oh, unfortunately. Okay. I'm sorry, that plan didn't work out. I see. Have there ever been resources that have helped you get night exam?

EN9002

As far as what,

Interviewer:

Resource Availability

Knowledge-creating Experiences

In-clinic Experiences

Emotional Context

Coding Density

Um, I guess it could be helped with transportation or maybe a reminder, whether that's been like a phone application, or maybe the office calling you could be a lot of things.

EN9002

Reminders...

Cues to Action

Interviewer:

Reminders. Okay. EN9002

Yeah. You know, they call you a day or so ahead of time and let you know, you have an appointment.

Interviewer:

Mm hmm.

So that's been pretty helpful. Yeah, okay. Sounds good. Yeah. So again, some things may be familiar to you, some things may not. So it's helpful to know that for you, most of these issues have not been a problem. If anything, it's being out of town, if I'm hearing correctly being out of town, or if anything reminders have helped you, too. Yeah. Okay. Sounds good. So we'll move on to the next one. So this theme is called cues to action. And I'll be curious to know a little bit about your experiences being diagnosed with diabetes, and the process after that, but things in this category relate to appointment reminders, which you have mentioned that helped you go to visits, they could also be the schedule of having an annual exam, maybe being prompted or encouraged to go to an eye doctor by your primary care doctor. Or if there was some type of light bulb moment that made you think I need to go to these appointments more often. So and I'll read this quote on the right, but so this theme is kind of related to moments or instances that made you kind of spring up and say, Okay, I need to do this. And so the quote is, I went for an eye exam because a doctor that I had, she examined my eyes. She said, I have to send you to the eye doctor. That's when I started getting these eyes hands. So with this slide, I want to ask what convinced you to start getting eye exams? Or who?

EN9002

I had little blood vessels in my eyes.

Vision Status

Interviewer:

And how did you know you had blood vessels in your eyes? EN9002

from the inside.

They look like hairs hanging on the inside of my eye. That you can't see from the outside but I can see

Interviewer:

Oh, okay.

So were there changes in your vision EN9002

Interviewer:

diabetes.

Wasn't changed, and my vision is just like, I don't know what they were just blood vessels from

Resource Availability

Knowledge-creating Experiences

In-clinic Experiences

Cues to Action

Vision Status

Emotional Context

Coding Density

And how long ago did this start happening?

EN9002

Maybe about four or five years ago?

Interviewer:

Four or five years ago. Okay. And do you have a primary care doctor?

EN9002

Not really. I see. I had a primary doctor at [redacted], but I found out she's dead.

Interviewer:

I'm sorry to hear that.

EN9002

So it's just the process of looking for another doctor.

Interviewer:

So I guess, um, do you remember the doctor who told you you had diabetes?

EN9002

No. That was years ago.

Interviewer:

I see. How do you remember finding out you had diabetes?

EN9002

I don't know. I don't know. I know I was at a doctor's office and something happened. I don't remember.

Interviewer:

Okay. I'm just closing windows. It's not so loud for you. Okay, um, so, I guess I'm curious to know, do you remember when your first eye exam ever was? Or even? Okay, how about

EN9002

I've been wearing glasses all my life. So

Interviewer:

Say more you say you've been wearing glasses all your life?

EN9002

I'm not wearing them now, because I don't use them when I'm this close to my phone. Interviewer:

Mm hmm. That makes sense. So do you remember going to an eye doctor? A lot, since you always wear glasses?

Resource Availability

Knowledge-creating Experiences

In-clinic Experiences

Vision Status

Emotional Context

Coding Density

EN9002

I think maybe once a year, once every two years.

Interviewer:

I see. So you mentioned how you started getting eyes and about four or five years ago? And that seems to be around the time that you were diagnosed with diabetes?

EN9002

No, I've been diagnosed with diabetes for years.

Interviewer:

For years. Okay.

EN9002

Oh, yeah.

Interviewer:

I see. So there seems to be, I guess looking at the timeline, a time in which you were diagnosed with diabetes years ago, but it seems that you started getting eye exams for diabetes about four or five years ago.

EN9002

more often and went to that's when that's when I first started saying Dr. [redacted].

I've always had diabetic eye exams. But when the blood vessels started coming in, that's when I go

Cues to Action

Interviewer:

I see. Okay, that's good to hear. So, since being diagnosed with diabetes, you remember getting your annual eye exams? Yes, I've seen and what, what motivated you to get those annual eye exams, even before you found out about blood vessels?

EN9002

I'm just trying to keep up with new frames.

Interviewer:

Yeah, 'cause you were wearing glasses already. Like, okay, yeah. So did you know that blood vessels could form in your eye?

EN9002

Well you know, I didn't know until that happened.

Interviewer:

Until it happened. Okay. Yeah. And how, how was that experience? I guess, learning for the first time that that could happen. And that it did happen.

EN9002

Interviewer:

whatever. And that's what I like to see Dr. [redacted] and they did a laser surgery on my eye.

went to an eye doctor and they told me that I had some whatever it was blood vessels that popped or

hanging from my head. And nobody else could see it. But me so I figured it was on the inside. So I

I was like seeing I don't know. It was like, seeing something in your eye. I thought something was

Resource Availability

In-clinic Experiences

Cues to Action

Vision Status

Emotional Context

Coding Density

Right. And thank you for sharing that. And when and when you you met you described say seeing something hanging from your eye. Were you surprised when you went to the eye doctor and you got that checked? Were you surprised or did you think it was related to your diabetes?

EN9002

No.

Interviewer:

Okay, no. Okay. And so was that surprising to hear that it was related to diabetes?

EN9002

Yeah, it was.

Interviewer:

I guess, um, before knowing that, what did you think it was?

EN9002

Here? Hmm, that's what it looked like, long strings of hair.

Interviewer:

Mm hmm. I see. Well, that makes sense. So if I'm hearing correctly, it seems that you know, you sound like you've been pretty good with your eye exams going every year, partly because you wear glasses too, when you just need your new frames, which is understandable. And then at some point, there started being this hair or something hanging down in your vision,

EN9002

Every so often they get a blood vessel pops up, and sometimes it goes away by itself before I can even make an appointment.

Interviewer:

I see. So that makes sense. And I guess when you were diagnosed with diabetes, do you? Did anyone ever tell you that it could affect your eyes?

Knowledge-creating Experiences

EN9002

I don't even remember, it's been so long ago. I just do what I have to do I take.

moving?

And so when and keep it

Interviewer:

Yeah, no, that's understandable. Okay. Oh, thank you so much for answering the questions related to this slide. I think we covered most of it. So I appreciate you opening up about.

EN9002

Okay, this is such a traumatic experience in my life.

Interviewer:

Resource Availability

Cues to Action

Vision Status

Coding Density

I'm so sorry. I really appreciate your strength and being able to talk about it.

EN9002

I'm fine. I'm fine with it.

Interviewer:

Now, I really appreciate it. Because it's people like you, that helps us to say, Okay, how can we make this system better? Because, you know, you're doing everything right, you're getting, you're going to your appointments, you're going to your exam. So how can we make sure the system is working at the best for you? So again, I think you just sharing and being honest about this for me. Okay, so our next theme, and we I think we've already talked about this a little bit, but I'd love to hear if there's more related to it. It's called knowledge creating experiences. So a good example, is you talking about how you saw this thing hanging down your eye, and you went to an eye exam and learn that diabetes was the cause of it. Similar to that, I'm curious to know whether you had education or had learning experiences from other things. This could be family or friends with diabetes, or maybe another doctor who told you about it? No. Okay, or online resources, forum support groups. Okay.

EN9002

My experiences all for myself. All my own.

Interviewer:

Wonderful. Okay. I see. And did you ever try to seek kind of more information about like diabetic eye disease once you were diagnosed with it?

Unknown Speaker No.

Interviewer:

Okay. Did you have the desire to or for you kind of let your experiences? EN9002

So the last time I went was seeing Dr. [redacted]. And it had already cleared up on its own.

like I said, Now, sometimes it comes back and it disappears before I can even get to my appointment.

No, I had my experience, I let it go. So if it happened, it happens. You know, whenever it comes back,

Knowledge-creating Experiences

Emotional Context

Interviewer:

Okay, so now we're gonna move on to experiences in the clinic. So, um, this one overall is related to how you felt in the clinic. So, I guess we could start with, how is the clinic experience for you? How's it going to get your eyes in?

EN9002

unless it's absolutely necessary, but I'm fine with the clinic.

Good, fine. I like to clinic, you know, I really won't see anybody else but Dr. [redacted]. You know,

In-clinic Experiences

Interviewer:

Okay, good. And what has made that experience positive?

EN9002

Resource Availability

Knowledge-creating Experiences

Coding Density

Seeing her. Yeah, yeah.

Interviewer:

Guess what parts of that clinic experience had made it good versus bad. EN9002

about any of them. They're good.

the door, we had, you know, me, her, her assistants and the nursing staff. Very good. nothing bad to say

I didn't see any bad thing about it. We hit it off. And the first time I walked through

If you can, I didn't,

Interviewer:

In-clinic Experiences

Emotional Context

And this could be related to experiences, Dr. [redacted], or even other eye doctors you've seen? Are there any things that have made the experience? Not so good?

EN9002

No.

Interviewer:

Okay. Great.

And do you ever feel that? Um, I guess in terms of communication, do you feel that with your doctor or with other eye doctors? Do you feel like you can understand what's going on? Yeah. Okay. Yeah.

Okay, good. All right. So our next slide, and you talked about this a little bit, too. It's called vision status. So you mentioned you've worn glasses all your life. And you also mentioned how it was this kind of hanging or this hair picture that made you go to the eye doctor. So now I'm curious to know, how is your vision today? And how do you feel it has changed in the past, say, four or five years?

EN9002

glasses. Okay. You know, when I go to the eye doctors is pretty much the same, I believe.

I don't think my vision has changed. Not that I know of. I don't haven't seen much of a change in my

Vision Status

Interviewer:

Okay, that's good to hear. And would you agree that it was that kind of hair like, image that made you see that made you kind of see a doctor that that other time in terms of four or five? Oh, yeah,

EN9002

know, I had the insurance that they were paying for. So I went every year. Right? But if it wasn't for

if it wasn't for that, I probably wouldn't have gone when I did. But on a regular I was go because you

Cues to Action

And I went to my my regular doctor, and they told me what to do. They told me to go and see a specialist. That's when I met dear old Dr. [redacted].

it was a fly flying around my head and nobody else work could see it. And it started getting worse.

that, I was as a matter of fact, I was at work and it just popped up all of a sudden I like I said, I thought

Interviewer:

And the rest is history.

EN9002

The rest is history. She's my buddy

Interviewer:

Resource Availability

Knowledge-creating Experiences

In-clinic Experiences

Cues to Action

Vision Status

Coding Density

I love it. So you mentioned that you were at work. Do you have to use computers or use your eyes a lot when you're at work?

EN9002

Well, I'm a chef. So yes.

Interviewer:

Okay. Yeah.

So you were actively kind of using your vision using, you know, working around and then you saw it, so it was very obvious to you. I see. I see. What's your favorite thing to cook?

EN9002

I do Italian food. Oh, wow.

Interviewer:

That's amazing. And we see and you're still working these days to

EN9002

working part time now. Okay, I'm on live in personal care assistant. I see. I have ever cooked since this pandemic. Oh, I see.

Interviewer:

I hope that can change soon.

EN9002

I hope so too.

Interviewer:

So we're on the path with these vaccinations so fingers crossed. Okay. So you mentioned how you were at work. cooking, and you notice that? Do you think that you're more aware to your vision these days? Or you kind of just kind of go about your days?

EN9002

every day. Except for when I'm close to the phone.

I'm aware, and I go about whatever I have to do, but you know, I keep an eye on it. I wear my glasses

Emotional Context

Interviewer:

Okay, good. So you wouldn't say that you're so I guess you're aware. But would you say that you're more aware than you were say, like, 10 years ago?

EN9002

Yeah.

Interviewer:

Okay, so a little bit more kind of watching for your vision, but not in the way that it interferes with your life or interferes with what you have to do. Make sense? Okay, thank you for answering that

question. So now, our next one is called competing concerns. And it's a little bit related to the resources that I mentioned up above. But, um, examples of, I guess, if you have an appointment, but something else is in the way, with other with other people I've talked to sometimes this could be employment, for example, if you have a job that, you know, spans a whole day, and that's the only time you can get appointments. It could be childcare, especially during the pandemic, when sometimes school has been out or daycare has been out. It could be other health problems, perhaps you were in the hospital and you couldn't go to your appointment, or other issues such as substance use or other complex that have prevented you from getting exams. So I guess to your comfortability, has anything similar to this prevented you from getting eye exams?

Resource Availability

Knowledge-creating Experiences

Cues to Action

Vision Status

Coding Density

EN9002

No. Interviewer:

Okay. No, that's good to hear. And I guess, I mean, based on our, what we talked about before, it seems that you've been pretty on top of it with your eye exams. Wonderful. Has, I guess, in the context of the pandemic, did you switch over to virtual visits? Or I guess, talk to me a little bit about how that is?

Actually,

EN9002

I went to see I did see an eye doctor last year. Okay. Um, I don't remember when I know it was sometime after I got out of the hospital.

Interviewer:

Okay.

EN9002

So I did go and see an eye doctor and had my eyes examined. And then I see a doctor one day before the year was over with also.

Interviewer:

Okay, last year. Yeah. Okay. So, for you the pandemic, or I know, there was a large cancellation of visits, but that didn't. did that affect you at all? Sounds like it did. Okay. Oh, great.

EN9002

So I had to go. Like I said, I had to go, I think she was in [redacted]. And she wasn't in [redacted] she was covering in [redacted], I had to go to [redacted] to see her because I said, I won't see anybody else.

was out there, that's where I went.

Don't let anybody play with my eyes. trust her. Yes. to the fullest. You know, so when they told me she

In-clinic Experiences

Emotional Context

Interviewer: So you went?

EN9002

I won't let anybody else touch my eyes.

Interviewer:

I see. And your

EN9002

Resource Availability

Knowledge-creating Experiences

In-clinic Experiences

Cues to Action

Vision Status

Emotional Context

Coding Density

regular regular exam? Yeah. Okay, comes with doing laser surgery or anything else.

Interviewer:

Wonderful. And

EN9002

she's off in another country somewhere. And I have to fly over there just to see her while traveling. I'm not going to be thrilled to be with her now. That the stuff you'll go where she goes, but only so far, only to a certain extent. 10 miles and that's it.

Interviewer:

Oh, wow. Well, that's wonderful to hear. because well, so I'm assuming you're probably based closer to [redacted], or

EN9002

I'm in [redacted].

Interviewer:

Okay, so you're in [redacted]. So [redacted] isn't too far away, but you're willing to go there. Okay. Yeah, I see. And has there ever been a doctor who similar to Dr. [redacted] you're willing to go to other locations to see?

EN9002

No, I mean, if she, if she can't do it, and she recommends somebody, then I'll do it. If If not, then I'll wait.

Interviewer:

Okay.

EN9002

But if I get a recommendation from her and she tells me that they're okay. I think when when she first did my eyes surgery, there was a doctor that was with her. That did it. They did like a tag team type of thing where they did [redacted] did my eye surgery. Right? So and I don't know who he was...

Interviewer:

but it went well?

EN9002

Oh, yeah. Yeah, they had a big ol patch on my eye look like the size of a tampon. Oh, yeah, like that for three days. I mean, like, Wow, really?

Interviewer:

To keep everything safe.

EN9002

Whatever.

I'm glad you had that positive experience. Wow. And I guess going back because, again, one of the themes was talking about trust with your doctor. And it seems that for you and Dr. [redacted], that trust, did that take a long time to build? Or I guess you mentioned that you guys hit it off quickly.

Resource Availability

Knowledge-creating Experiences

Cues to Action

Vision Status

Coding Density

EN9002

I'm the type of person that if I really don't trust you, you're gonna forget about it. So what do you think we so we hit it off the very first day.

Interviewer:

So and what do you think, helped that? EN9002

reacts with other patients and how they react with her. Because you know, she is kind of loud. So her

time she made the time for patients like you. And yeah, I can hear her when I go and visit how she

she takes the time out to enjoy her patients like me. Wonderful. So you found that she was making the

I think she's an all around good person. She's good spirit is very happy. He enjoys what she does. And

In-clinic Experiences

Emotional Context

Interviewer:

voice does carry. She's She's just a complete sweetheart.

Glad to hear that positive experience. I think this might relate to actually our next slide, too. But again, we could talk about other things related to this. It's called emotional context. So it seems like for you, you mentioned how unfortunately, that experience with that first blood vessel was a traumatic experience. And, you know, that's not something to ignore. But for you, it seems that, you know, you also grew from it. And you know, to be aware of what when something's happening with your vision, but not letting it control your life. So, um, but you also mentioned how you didn't necessarily, like, look up more information about it or want to know more. And so I'm curious. So how, yeah, so I'm curious to know, how does that I guess emotionally, how do you feel about overall, your diabetic retinopathy and kind of the unknown? But also what, you know, does that make sense?

EN9002

about it. Don't worry about it. I know I'm in good hands with Dr. [redacted]. And I've got God on my

of it. It was rectified. And I know what to do. Now, if it ever happens again. I'm really not worried

Well, it really didn't bother me. I was it takes a lot to really get me riled up about anything. I took care

Interviewer:

side, so I'm good.

And you mentioned how it seems that you know through Dr. [redacted], she's going to have a form of support. And you also mentioned God so I'm curious to know if there are other things that have helped supported you through this experience, whether it's friends, family, religion,

EN9002

ones my family that knows people who are on my list of emergency, which would be my sisters and my

know. Except for you know, maybe if I post something on Facebook, about my closest friends and only

A little bit of family. I'm very private person. I don't tell everybody my business. So my friends don't

Interviewer: 39:29

mother. Other than that, I family don't know what I go through.

I see. And are there other family members that have diabetes?

EN9002

Resource Availability

Knowledge-creating Experiences

In-clinic Experiences

Cues to Action

Vision Status

Coding Density

Just about everybody in my family has diabetes. Except for I don't think my brother has it. I know my sisters have it, my mother has it, my father had it.

Interviewer:

I see and do you know they had gone through any diabetic eye disease?

EN9002

As far as I know of no. I think mine is the only one...well my sister, my baby sister, has what do you call it ketoacidosis? I think hers is worse than mine. I don't know. I've never had and I've never had any she's always been in the hospital for it. She would get sick and all that stuff but as far as anything else I think they're like borderline diabetics my sister and my mother. I think I'm the only, well besides my baby sister, the only one that takes insulin.

Interviewer:

ever shared their experiences? Have you ever shared your experiences with your eyes?

You mentioned how you consider yourself to be a private person. Have you and your family members

Emotional Context

EN9002

No. They knew about my eyes 'cause at the time we were all here in [redacted] when it happened.

Interviewer:

I see. Other forms of support you can think about? In terms of going through this experience or healing from his experience?

EN9002

No that's about it.

***End of formal interview***

------- START OF INTERVIEW -------

Competing Concerns

In-clinic Experiences

Resource Availability

Vision Status

Cues to Action

Knowledge-creating Experiences

Emotional Context

Coding Density

Interviewer: Okay, perfect. Great, so, now that we’ve finished all the housekeeping things, we can get started on some of these questions. So, again, like I mentioned, we appreciate your honest thoughts.

Competing Concerns

In-clinic Experiences

Cues to Action

Knowledge-creating Experiences

Coding Density

You are the expert of your own experiences. I may ask you to say more or give more details, not because your answers aren’t good, it's just because I want to hear what you mean by some of your answers. There are no right or wrong answers, and like I mentioned, all of your responses will be anonymous.

EN9003: Yes. I'm going to help as much as I can because I understand every single person is different, you know.

Interviewer: Mm-hmm, exactly. Exactly, EN9003. Okay, so, our first topic is about resources. In the past, a lot of patients have talked about what has stopped them from getting eye exams. It could be lack of insurance, not having the money, not having a bus or a car to get to the doctor’s office, being homeless, or even being in prison or jail. One quote from a patient was, “I was in a tough place, you know, I was on the streets after squinting for two years, I finally went back to see an eye doctor.”

So, with this information, I wanted to know, have there ever been resources…or have you ever been limited in resources and not been able to go to an eye doctor?

Yes. Yes, it happened to me back around 2016, when they stopped it because I had no

EN9003:

Interviewer: I see. So, you mentioned in 2016, you stopped having insurance. Was this the HUSKY Insurance, or…?

that’s when the problems occurred in my eye.

insurance, and then that stopped me to having an eye exam back then. And then from there to ’18,

Resource Availability

EN9003: No, I used to work for the company, and then I quit back then from that company.

over here. I forget the name. From that to ‘18, when the problems occurred for me, in November ‘18, I

then I used to have a treatment on my doctor, private doctor, through that insurance, back then, locally

And

I couldn’t afford to

was not having any treatment in between it, because it was not affordable for me.

go and see a million doctors. And then, I did not go…on top of that… even I was realizing I was having some issues on

my eye, on my vision. It was getting blurry

important,

I did not think it was so

Vision Status

Emotional Context

But I did not understand that it was so important for me to go to see the doctor and continue the treatment.

vision.

Interviewer: Thank you for sharing that. It sounds like when you were working at your previous job and you were having insurance, it seems like you were going to eye appointments more regularly.

Would you say that?

EN9003: Mm-hmm, I guess so, yes.

Interviewer: I see. So, then when you lost your employment, or you ended up leaving your job, there was that gap in time.

EN9003: Yes.

Interviewer: I'm hearing you say that you didn’t think it was important. What made you think that?

EN9003:

Interviewer: Mm-hmm. EN9003: Hello?

this real quick.

couldn’t understand the hardness, I would say, that I was running. …oh, hold on, let me just answer

want you to lose your vision. Okay. I understand that, but for me at that time, having 20/20 vision, I

because all I’ve seen, they’d given me a treatment, and they tell me, we’re doing this because we don’t

explanation from the doctor to the patient and let me understand the situation that I was going through,

Because of the way the doctor…I mean, I don’t blame at all, but I think there was a short

Competing Concerns

In-clinic Experiences

Knowledge-creating Experiences

Emotional Context

Coding Density

Interviewer: Yes.

EN9003: Okay, yes, I'm here. Interviewer: Yeah, no worries.

EN9003: Okay, Miss Interviewer. I was trying…I was explaining to you what the doctor told me, I don't think they shared enough information for me to understand it, because she tells me, you’re going to lose your vision eventually, but she’s not telling me how that can, you know, make so much damage too soon.

You know, so, that’s why, I mean, I was waiting, hoping, to get a new job and then go back

Resource Availability

So, I did not pay so much attention to that because I have 20/20 vision.

all of a sudden, I got to see some blurry sometimes, and it was back and forth. But I did not understand

And then

to those treatments.

Vision Status

I was not educated, like I understand now why

what it was about, and the increased pressure in the eye.

all the things that happened.

Interviewer: Yeah. Thank you for sharing that. It sounds that you were consistent with your appointments, but it seems that in those doctor appointments you weren’t given enough of an explanation as to how fast and how serious it could be.

EN9003: Exactly. You have the right words.

Interviewer: Yeah, you explained it very clearly. And so, describe to me between when you found employment and found insurance, describe to me that experience of being able to go back to eye appointments.

thought it was something like, okay…it isn’t getting lost. So, I mean, it was like I was in that stage,

Like I said, I mean, I didn’t feel, you know, they needed to have that treatment so bad. I

EN9003:

Interviewer: Right. You mentioned your vision getting blurry. What else got you to start going back?

day, I had to wake up early in the morning. So, I took a day in the middle.

related to my vision. It was hurting my vision. So, I worked the shift straight, nightshift, and the next

went to work with this company that I worked overnights. I mean, understand, my diabetes, it was

Oh, when that situation, when it happened that day. It was a situation that I worked…I

how bad my eye was.

flow. I was not rushed or worried to go back to the treatment, because like I said, I did not understand

okay, I’m going to need glasses pretty soon. It was like kind of, like, okay, I mean, I’ll go with the

Cues to Action

EN9003:

to blow out. I had a 69 pressure on that eye, on the left eye.

my left eye clouded up. And they told me, if you don’t do anything, your eye is going to pop out, going

was like…I started crying. I went in tears. And then at this moment, my right eye…starts seeing, but

his name. I know it was Ian. So, when he reads the chart, he tells me, “You’re going to be blind,” and I

teachers…and I don't know what happened. So, just one doctor…I don’t remember his name…I forget

but I don't think they understand what I say, or they don’t want to listen. So, they are students and

So, I just went there, and I told them…this is the department for students. So, they start working on it,

and tell them that you need to have lasers done before your eyes get worse.”

listen, he’s got a clot at nine o'clock here and then he told me, “Well, you need to go to that department

giving me a treatment. And then one of the doctors, I heard him, he told one of the students, he said,

That’s when they told me and sent me where they have a Department out there, where they treat me,

So, in the days after, that increased again. So, I just ran out. I went back to the ER again in [redacted].

nothing. They couldn’t handle seeing me so soon.

arrangement to go back and see the regular local doctor, but they were so booked up, there was

But I was like, okay, this incident happened. I mean, so, I took a day off after that to start to make the

eye, I could drive myself back home, that day.

went back to normal. I just walked out there, driving my car myself. I mean, even with drops on my

with your eyes. If you don’t treat this, you’re going to have a lot of problems. And that day, my vision

vision went back. So, he told me, you need to see your doctor immediately because you have a problem

the doctors…I don't know if it was a practitioner or the doctor…they put eyedrops on me and my

going to take care of you. You need to go there. So, I just went to the ER in [redacted], and then one of

Somebody tells me, listen, if you go up there, then tell them that you have no insurance at all. They’re

So, long story, short story, I just end up going to, I mean, a day after, [redacted] University.

said they tell me they cannot do anything for me.

did is they just checked on me, and then because I didn’t have any insurance at that moment, so they

then they drove me back to the hospital, emergency room, ER, right here in, where I live. What they

So, all that, I mean, rushed back. I mean, I called my father-in-law. They went and picked me up, and

to see, and then all I was seeing, it was a big cloud in front of me, not even seeing the hood of my car.

over and then rest, you know, for about an hour. So, when I wake up for the noises around me and try

my head, I was thinking that I was tired from that shiftwork the couple days before. So, I just pulled

All of a sudden, I just started seeing more cloudiness, and then I just pulled over, and then I was like, in

I didn’t have education for that.

sudden, I didn’t understand that, nothing about high pressure inside the eye, nothing, like I said before.

down, I just saw that in my both eyes, not on the left eye only. Actually, on both eyes. And then all of a

was…when you put water, right, on a window, and then you see that water the first drops coming

And all of a sudden, I saw like…I call it water, but I think it was tears. I mean, that’s what I thought it

of the traffic…usually regularly seeing it far away.

that my distance of seeing, and I was like, okay, something is wrong on my eye because I don’t see any

not normal. This is something happening, and then all of a sudden, I noticed that I was not reaching far,

me, oncoming traffic. I was seeing it was a really huge bright light, and like realizing, I was like, this is

sudden my eyes started getting really blurry, and then all of a sudden I felt the light that was coming at

So, on my way to go to work, like 5:00 o’clock in the morning, I wake up, I was driving it, and all of a

Competing Concerns

In-clinic Experiences

Resource Availability

Vision Status

Cues to Action

Knowledge-creating Experiences

Emotional Context

Coding Density

Interviewer: You had 69 lasers?

Competing Concerns

Vision Status

Knowledge-creating Experiences

Emotional Context

Coding Density

EN9003: No, 69 pressure. The pressure. The eye pressure inside the eye. Interviewer: Oh, the eye pressure. Okay, yes, that’s pretty high.

guess it was too late when they brought me there, because when they did the laser, and they started

there, and then he helped me out to sign all the paperwork to get insurance and everything…but then I

department out there, and that’s when they took me down here to [redacted]. He brought me down

a needle and take the fluid off. So, in that time, so he has the permission for whoever’s running that

It was so high, it was 69. So, and then, he just asked me permission to poke the eye with

In-clinic Experiences

EN9003:

Interviewer: I see. Wow! Thank you. EN9003: Yeah, it was a lot.

again through you guys out there, at [redacted].

stuff, and then that’s when I started to see that I had all these problems, and then I started treatment

twice a week, so he was poking the fluid of the eye with the needle until they did the lasers and the

my eye. So, all that time, the eye was clouded. So, he was asking me to go there to see him, I mean,

from that time, it took me to [redacted], it went on three more weeks before they did the first surgery on

Interviewer: Yeah, thank you, EN9003, for outlining that story. It seems like your way of getting insurance back was through that emergency.

EN9003: Mm-hmm, yes.

Interviewer: It sounds like you did go through a lot, and it sounds like you’ve also grown in being able to explain it in the way of how it happened bit by bit. And I think that experience with driving and seeing those changes in your vision seemed to be the starting point for you getting back into seeing an eye doctor. Would you say that’s close to it?

EN9003: Yes.

Interviewer: Again, thank you so much for answering those questions. As you are telling your story, you’re already answering other questions I was planning to ask. So, you’re doing a perfect job. I wanted to know, in terms of today, how do you keep up with your appointments? Do you have a system that reminds you? Do you write it down? What keeps you on top of your appointments?

with the appointments next to it and the planning. Between my sisters…and then one time I used Uber

I mean, they’ve got all the papers when you walk out of the office. You have the sheet

Cues to Action

EN9003:

Interviewer: I see. So, they also are aware of when your appointments are, or will you just ask them, oh, can you drive me to this appointment?

alternate, and then they drive me down there.

different district. So, it's like 45 minutes distance. Between my sisters and my mother-in-law, we

when I was driving from here because I live 40 minutes away, because I live in [redacted]. That’s a

Resource Availability

EN9003: What's the question again, I'm sorry?

and diet control to improve the eye health and then the diabetes itself.

another hospital in [redacted], where they work on all this, and they help the people on diabetic control

then they have another university in [redacted]. I forget the name of it right now. And they have

cells and stuff like that and education about macular degeneration disease, diabetic retinopathy. And

they have some other ones, like [redacted] University. They really work really good on that, on stem

some of them are really fake, and they are more business and trying to sell products, stuff like that. So,

I said, some other ones, I don’t even understand what they’re talking about, or I don’t care about it, and

education. So, from some of those videos, I’ve been taking what I think is helpful for me. Because like

information about it, like colleges, universities. They’re speaking and they have real facts and

So, YouTube, they have a lot of crap, [laughter] but they have a lot of good, really good

Interviewer: I was asking, do your family members remind you of your appointments as well?

No, no, I can remind myself, and then they remind me too. You know, they pay attention

In-clinic Experiences

Vision Status

Coding Density

EN9003:

Interviewer: Sounds good. It sounds like you keep your papers with the appointments on it and kind of remind yourself, and then you work with your family to figure out transport.

on that, so they’re aware about it, even my wife. So, we all, all the time, we keep an eye on it.

Cues to Action

EN9003: Yeah.

Interviewer: Sounds great. In terms of thinking about times where you have missed an appointment, what do you think was the cause?

EN9003: Missing appointments? Interviewer: Yes.

missed an appointment it was because I had no one to drive me down there, but I don't think I missed

I think I haven’t missed much [laughter] since that happened. But I mean, I think if I

EN9003:

Interviewer: That sounds great. So, now, I want to move into another theme, and this one is called Knowledge Creating Experiences. You mentioned how you learned a lot about diabetic eye disease through your own experiences, and I'm curious to know whether you ended up looking up information or learn from family and friends. How did you get more information about diabetic eye disease?

all the eye diseases are from, and how exercise and how to reverse all this, how that impacted me.

was…and I’d like to understand the study and be aware of where diabetes disease came from, and then

So, being someone that was always willing to help and work…I had to focus on something that

crash on my life.

[voice cracking] I'm sorry. It was a shocking moment, you know, so, crash, my life. I’ll say, this was a

and keep my mind busy and try to focus on something because it was a shocking moment. I’m sorry.

blind by myself…I mean, kind of blind. So, I had to find out something to help me to entertain myself

mean, from being a truckdriver and being on the street, and after this incident happened to me, kind of

Thanks to the Internet, YouTube, might as well, because being…like I told you before, I

something happened, but I don't think we missed any.

any appointments. I think it was either switching schedule for any other reason, through the building or

Competing Concerns

Resource Availability

Knowledge-creating Experiences

Emotional Context

EN9003:

Interviewer: That sounds amazing to hear the journey you’ve been through in terms of learning for yourself, finding those resources, seeing the ones that were good and seeing the ones that were bad. So, it's great to hear that you looked for that knowledge and you found it. Thank you, EN9003, for sharing that.

That’s where I get more of the information. I really try to understand and educate myself.

Competing Concerns

In-clinic Experiences

Resource Availability

Vision Status

Cues to Action

Knowledge-creating Experiences

Emotional Context

Coding Density

EN9003: Oh, any time, I mean, thank you guys for helping.

Interviewer: Yeah, of course. I mean, you are also helping us, so it's a two-way street. I wanted to know too, do you have any family or friends with diabetes, and did you share any common experiences with people you know.

EN9003: I'm the expert in the family right now. [laughter] Interviewer: Oh, you’re the expert.

this happened. It was really way out of control. They never put insulin on me. But after that, learning

diabetes, and right now, just for you to know, I mean, my diabetes was 14 up to 16, my A1C, before

I’ve got four sisters. They all have diabetes. Before I used to be the one with the worst

EN9003:

Interviewer: Wow!

now is 6.3.

now, I'm the expert. My sisters, I'm helping them when their diabetes is out of control. My A1C right

how to control the pancreas, the fluid, how to control myself in food and some other interests. So, right

EN9003: Just to bring that down so much in less than two years. Interviewer: Congratulations!

EN9003: Yeah, thank you. My doctor was so happy, my main doctor. He was like, well, it must’ve happened for you to learn how to control.

Interviewer: But you got there. That is not an easy feat. So, congratulations for that, and I hope you celebrate that in yourself. It's great to hear that you’re also helping your other family members and you’re the expert. [laughter]

and they are too high…I mean, when you understand how your body works, it's so much easier to

Yeah, that’s what I'm becoming now. Now, when they’ve got a problem with their sugar

EN9003:

Interviewer: Yeah, that’s amazing. Thank you, EN9003, for sharing that.

starts working by itself, little by little, and it's getting back.

and how you prepare stuff like that, you almost don’t need medication no more because your body

understand it. I mean, when you’re fasting, after night, and how the vegetables and the natural foods

EN9003: Thank you. [voice cracking] I'm sorry, I mean, I just break up a little bit myself.

Interviewer: No, that’s okay. It takes a lot of strength to be able to share your whole experiences. So, again, I really appreciate this process for you and you sharing this with me.

you think I can put an injection on me and have it making no changes.” So, even with the diet I'm using

told Dr. [redacted], “Listen, I don’t want this anymore because for two years and being through this,

disappointed to have the same treatment monthly…and I just

Because, I mean, I was kind o

I mean, we’ve got to keep it under control.

I'm being honest to you, to have that injection in the eye every month, and she makes me understand it.

me pictures, like right now for the injection. When I was kind of confused to have the injection, I mean,

expert, right. [laughter] She will tell me and then repeat it again and then she goes back, and she shows

[laughter] You already know. Yeah, like, okay, because I want to hear it from the expert. She’s the

through. Sometimes I'm asking questions and she tells me, “You know the answer, EN9003.”

EN9003: Thank you.

Competing Concerns

In-clinic Experiences

Resource Availability

Vision Status

Cues to Action

Coding Density

Interviewer: So, we’re getting to our last few topics. You mentioned some experiences in the clinic and you mentioned earlier how your first experience with an eye doctor was that you didn’t have much explanation, and so you couldn’t understand the harm diabetes can do to your eyes. So, with that, I want to know, what has made experiences good in the clinic, and what has made experiences bad in the clinic.

Dr. [redacted], she takes the time to talk to me and make me understand the problems that I'm going

know.

concern in their careers, and change their careers because they don’t know how to handle patients, you

been helping her, they’re really good, but they have one or two of them, they need to have better

Some of the doctors out there, I mean, I have to tell you the truth, some of those other doctors that’ve

She has the best attitude.

right answers. So, right now, Dr. [redacted], I’ve got to tell you, I love her. I'm not going to change.

some of the clinics where I used to go before, because I was asking questions and then not really many

Before, I felt like I was a number. I was another number, not even a patient. I mean, for

Knowledge-creating Experiences

Emotional Context

EN9003:

f

Interviewer: Thank you, EN9003. You first described, saying that you felt like a number, and I think you described very well what you mean by that in those experiences where you weren’t really being taken care of or you don’t feel like you were being paid attention to. So, it's good to see that you’ve had positive experiences, particularly with Dr. [redacted]. So, you have that comparison of bad and good. So, that’s great to hear.

But right now, it seems like it is a good experience, the way I am right now.

that. I did not understand that.

listen, I need to do what I need to do, and you just come back next month. I mean, I didn’t understand

need to quota with the numbers of patients, and then they just want to wipe off the office, and then

that, I mean, some of the doctors…I don't know if they don’t want to waste their time because they

I had a good experience with the office out there at [redacted] and some other ones, but before

but it's coming. I mean, I see improvement, little by little, but it's coming.

been doing, plus the injections, yes, it's coming down, little by little. It's not happening big in the eye,

now, I have more control. I understand it there. If I have control, my diabetes, and all the stuff I’ve

Again, you’ve answered a lot of the questions on the slide, so I don’t even need to go bit by bit.

EN9003: [Laughter] Okay.

Competing Concerns

In-clinic Experiences

Resource Availability

Cues to Action

Knowledge-creating Experiences

Coding Density

Interviewer: Yeah, you’ve done a great job. I wanted to ask a couple more lingering questions. You mentioned employment affecting your insurance and also affecting you going to appointments. Has there ever been times, whether it was employment, taking care of family, that have prevented you from going to appointments?

EN9003: Say that again. Can you repeat that question again?

Interviewer: Yes, sorry, the question was a little confusing. You mentioned that you’re pretty good with going to your appointments, but has your job ever prevented you from going to an appointment during the day?

there. So, I have to go back.

careful, what I'm doing, the chemical supply and the wash and stuff, and the details, skip here, skip

wax and truck wax. I’ll do all my vision allows me. That’s the kind of job where they need to be really

truckdriver, so I'm not able to drive the big trucks anymore. I'm doing right now, some kind of house

whatever stuff I can handle here or there because of my condition. Like I said, I used to be a

No, not at all, because I'm not employed. I'm kind of self-employed right now doing

Emotional Context

EN9003:

Interviewer: That’s good to hear. In terms of now, how do you feel overall about your vision? How do you feel overall about your relationship with diabetes and your vision?

regulations they have and stuff like that. I don’t feel right now at this moment my vision is stable, that

guys and then I want to have my vision back. The way the system is, I don’t understand the FDA, the

honestly, I was hoping that I can get my vision back. Honest to you. I'm working together with you

Well, that’s a good question. [laughter] Right now, I'm doing the treatment, and

keep my head, and make me feel like I'm not useless. You know, I still can do something.

single one of them. Some of them can allow me to do the work. So, that’s why I try to keep moving and

it's not like I'm running a business with people that don’t know me. I don’t have to explain to every

people that I know. So, they understand what I'm going through, and I can call them up and cancel. So,

So, if I don’t feel that vision is not allowing me that day, most likely, what I'm doing is friends and

EN9003:

Interviewer: No worries.

EN9003: I care about that, you know. I don’t want that to happen, but if it happens, it happens, you know.

blind. [voice cracking] Sorry.

it is controlled. I'm not afraid to be blind at this point. Honest to you. Like before, I was afraid to be

Vision Status

Interviewer: Again, thank you, EN9003, because it takes a lot of strength to be able to say that. It's very powerful for you to say that you’re not afraid of loosing your vision and the way you were before. I think that takes a lot of growth and a lot of personal understanding of what vision means to you.

Thank you for that.

EN9003: Yeah, that makes me understand how valuable is life.

Interviewer: Yes. This is probably my last question. In the midst of the COVID pandemic, was there a time where you weren’t seeing your doctor?

Yes, I guess this is one of the times, with this pandemic, that they had to cancel one of

In-clinic Experiences

Resource Availability

Vision Status

Cues to Action

Knowledge-creating Experiences

Emotional Context

Coding Density

EN9003:

Interviewer: Okay, good. Did you ever do any telehealth or virtual visits? EN9003: No.

that, I think I went to all my appointments.

my appointments. I guess it was twice they canceled appointments because of the pandemic. Besides

Competing Concerns

Interviewer: Okay, then, I think we’ve reached the end of our conversation. First, I want to say, thank you again. You have done such an amazing job, first keeping up with your diabetes, understanding your situation, and then being able to speak so clearly about it. I want to open the floor to see if you have any questions for me.

EN9003: Right now? [laughter] Interviewer: Yes. It could be later too.

EN9003: Do you have a wonder stick that can help me get my vision back? [laughter]

Interviewer: I know you have the best doctors taking care of you, so your chance of getting your vision back, I think, would be in the hands of your doctors, but you have the best chance with them in your care.

EN9003: I was just kidding on that. [laughter] But I thank all you guys for all the hard work that you guys are doing for all of us. I mean, some people sometimes, they don’t even understand what you guys are going through to do all this stuff and have all this done.

Interviewer: Again, thank you so much, EN9003. This has been a great learning process for me just to hear about your experience. I hope it was also a positive experience for you, this interview.

EN9003: All right, thank you so much, and feel free to call me anytime you need to. Interviewer: Yes, no worries, okay, take care, EN9003.

EN9003: All right. Wonderful. Thank you. Interviewer: Take care of yourself. Bye.

EN9003: Bye.

***End of formal interview***

----START OF INTERVIEW----EN9004: Okay.

Competing Concerns

Resource Availability

Vision Status

Knowledge-creating Experiences

Emotional Context

In-clinic Experiences

Coding Density

Interviewer: So, the first theme, EN9004, is resource availability. And this includes issues with insurance, issues with money, transportation, not having a home, or even being incarcerated. Patients have mentioned these as issues that have prevented them from getting eye diseases in the past. And just a quote is - a quote from a patient was, “I was in a tough place, you know. I was squinting on the streets for like two years to see everything. That is when I finally went back to an eye doctor.”

So, I want to pose this first question for you; does this theme resonate with you at all in terms of not being able to get eye exams?

EN9004: I don’t understand your question because you said one thing and you actually - no, so, I’m not understanding the question. What resonates?

Interviewer: Yeah, no worries, yeah. It was a long question. So, have you ever had issues with resources; either not having insurance or money, anything like that? [interruption]

EN9004: Not as - I’m sorry, I apologize [interruption] - not at the moment. I haven’t right now, thank God.

Interviewer: Okay, yeah, that’s wonderful to hear. How about in the past? EN9004: No, I haven’t.

Interviewer: Okay. And has there ever been anything that’s prevented you from getting an eye exam? EN9004: No.

Interviewer: Okay. Okay, then. And I guess this is going into the next theme but what helps you to stay on track with your eye exams?

EN9004: Because of the experiences I’ve had with myself and I’m really concerned about myself. So, I make sure I get to the eye doctor.

Interviewer: And was there - I mean, do you use any reminder systems? EN9004: No.

Interviewer: Or do you have a person in your life that [interruption]?

No, no, no. I just remember my eye doctors. I have several but I always remember my

EN9004:

Interviewer: Okay. And has it always been that way? EN9004: For me to remember?

one with Dr. [redacted] on the 30th. So, I just remember the eye appointment. Yes.

eye doctor appointments. Hers is next week or the week after; it’s the Friday next week. And I have

Cues to Action

Interviewer: Mm-hmm.

week to three days a week to two days a week - made you remember that you have to go.

Yes, yes. Ever since the day I had my operation; ever since then, to one day - four days a

Competing Concerns

Resource Availability

Knowledge-creating Experiences

Emotional Context

In-clinic Experiences

Coding Density

EN9004:

Interviewer: And you mentioned this operation. Was this a long while ago? EN9004: It was in 2015. May in 2015.

Interviewer: Your memory is sharp. EN9004: Yeah.

Cues to Action

Interviewer: You always have [interruption] everything to the year.

first found out - was 50 in my eye. It was pain in my eye and I had to go to the emergency room. And it

That’s when I thought I had an eye problem. That’s why I know. My eye was - when I

Vision Status

EN9004:

Interviewer: I see. EN9004: Yeah.

turned to be my - whatchacallit - surgeon. Yes, and then, to be my doctor after.

just so happened I didn’t know him them; Dr. [redacted] was one of the doctors. He was also at that -

Interviewer: And so, you said how you went to the emergency department but there was pain in your eye.

EN9004: Yes.

Interviewer: Was this pain sudden or had it been…?

even know the day - on a Wednesday. And then, all the sudden, my eye kept blinking out and coming

Well, it’s weird to say that I was not - I was out a way and my eye got a pain on a - I

EN9004:

Interviewer: Wow. EN9004: Yes.

sure I had my eye exam.

which was - my eye pressure was 50. So, it was like having a baby in the eye. So, that’s why I made

back, going out and coming back. And then, it totally went out and I started getting a pain in my eye,

Interviewer: And so, you had this experience in 2015 but you had been diagnosed with diabetes back in ’89.

EN9004: Right.

Interviewer: So, how was your experience with getting eye exams between when you were first diagnosed and when that experience happened at 50?

EN9004: No, no, no, no, I got diabetes. I didn’t have the eye problem until - I don’t understand your question. I’m a little confused with that.

Cues to Action

Resource Availability

Vision Status

Emotional Context

Coding Density

Interviewer: Yeah, no worries. So, usually, when patients are diagnosed with diabetes, they can be told that diabetes can affect your eye.

EN9004:

They didn’t, they didn’t.

Knowledge-creating Experiences

In-clinic Experiences

Interviewer: Okay. Do you remember being told what diabetes can do to your body? EN9004: To my what?

Interviewer: To your body? Such as your eyes? EN9004: No.

Interviewer: Okay.

taking pills,” at that time. And I believe - I wasn’t even told that you don’t have to take the medication.

told me I had diabetes. She said nothing about it except, “You’ve got diabetes and you have to start

No, I was at - where was I? I was at - was I at [redacted] ? I was at a doctor’s office. She

EN9004:

Interviewer: I see. And at that time, were you getting annual eye exams? EN9004: No.

would’ve told me to eat better or whatever or whatever but I wasn’t told.

nothing about my eyes or anything. And diabetes runs in my family. And it seems like someone

on metformin, whatever it was back then. Glucophage - yeah, Glucophage back then, yeah. She told me

You could even try to like start eating correctly and not get on anything. No, first, right away, I was put

Interviewer: Okay. So, when did you start getting exams as a person with diabetes?

EN9004: Well, no consideration of it at all. I would just go to the doctor - I used to go to the doctor when we ended up being in that plaza. And then, I stopped going and I hadn’t been to the eye doctor in over like seven years or more. And it was my own fault, I believe, because I should’ve been - but I was told that I may get glaucoma. I was told that. So, I should’ve paid more attention to myself. I didn’t start going again until that visit when I had the eye problem.

Interviewer: I see.

EN9004: Which I blame me.

Interviewer: Well, you know [interruption]… EN9004: I don’t blame anybody else.

think you were busy - that was a busy time in your life or there were other…

Okay, yeah. Because there’s always a lot of competing things that can go on. Do you

Competing Concerns

Interviewer:

Interviewer: … things…?

know what was going on with my eye.

No, I was just scared to go, I don’t know. I don’t know why. I guess I didn’t want to

No, no.

EN9004:

Competing Concerns

Cues to Action

Resource Availability

Knowledge-creating Experiences

In-clinic Experiences

Coding Density

EN9004:

Interviewer: Okay.

EN9004: Which was crazy, you know?

Emotional Context

Interviewer: Yeah, I mean, you say, “scared.” So, did you have stories? Or did you have, I guess, fears as to what you would find out?

EN9004:

That’s what I’m saying. I was afraid of what I might find out.

Interviewer: I see.

somebody says that they’re seeing white, everything looks kind of white to them and foggish, that

And you know what? Whatever you - whoever you’re talking to, whatever you do; if

Vision Status

EN9004:

Interviewer: So, you started seeing white before you were having that eye pain and found out.

did. You start seeing white.

hardly see stuff. And so, that’s what made me afraid to go, which was crazy. So, it ended up the way it

happened to my eye, did not even realize it, that it was going on. So, I started seeing white and could

EN9004: Right. You know, out of my eye, I saw things as whitish. Not my eye white but things white.

Interviewer: Right, right. EN9004: Yes.

Interviewer: Okay. So, it seemed like there had been a gap - you’re saying about seven years and then, you had that incident in the emergency department. But then, after that, you got right back on…

EN9004: Yes.

Interviewer: … going to your eye exams, yeah.

EN9004: Yes, this is all the reasons why I make sure I go. Because right now, maybe you’ll get to it later. I’ll tell you later, probably get to it later. I won’t talk about that right now.

Interviewer: Yeah, I mean, again, [interruption]… EN9004: If you don’t talk about it, I will, huh?

Interviewer: Yeah. No, please. Again, feel free to share anything you’d like, or not share anything you’d like. So, again, I appreciate your perspective either way. So, thank you for sharing that experience.

Competing Concerns

Cues to Action

Resource Availability

Vision Status

In-clinic Experiences

Coding Density

I wanted to go back to - so, this actually goes into the next theme. You were mentioning how you had family with diabetes. After having your surgery, did you seek out more knowledge about diabetic eye disease either through your family or through online, through your doctors? Or did you just say, “Okay, I don’t need to know so much about this?”

from diabetes. So, I didn’t seek out any information because that’s what’s scaring me. It’s crazy to say

three of them had lost their sight. And now, my father’s first cousin, also, had lost his sight. They’re all

have three of my family; my father’s mother, sister, my father’s sister, and my father’s first cousin. All

Okay, I have several answers to that question. I didn’t seek out any more information. I

Emotional Context

EN9004:

Interviewer: I see.

So, you know, I thank God I didn’t go that way. What I’m saying is I was like that until my surgery.

ended up that way anyway.

but what happened to them, I was afraid that if they examined my eyes, they would tell me that. But it

Knowledge-creating Experiences

EN9004: So, then, there right now, that’s why I go so often because there’s this thing in my eye they say - that’s why they keep checking. So, I’m just making sure that - yeah, I didn’t seek any new information.

Interviewer: Okay.

If I want to know something, I have Dr. [redacted] at [redacted] or Dr. [redacted] . And I

EN9004:

Interviewer: I see. And you mentioned how you had had family members who went blind. Did you know it was from diabetes?

phone doesn’t do it so, I couldn’t get information. I couldn’t get it anyway.

can’t read to see - I’m reading better now but I couldn’t read to see anything. So, I couldn’t - and my

EN9004: Well, yes. Interviewer: Okay.

EN9004: Well, yes. My cousin was, yes - all of them were from diabetes. Interviewer: I see.

EN9004: Yes.

Interviewer: So, you’re saying that [interruption]…

EN9004: They didn’t tell me but the one that - the only one that’s left alive now has it from diabetes.

Interviewer: I see.

EN9004:

Interviewer: I see. So, if anything, that added to the fear that you were mentioning.

And they didn’t tell me but I’m assuming that it was diabetes.

Competing Concerns

Cues to Action

Resource Availability

Vision Status

Knowledge-creating Experiences

Emotional Context

Coding Density

EN9004: Right, right. Interviewer: Or why you - mm-hmm. EN9004: Yes.

Interviewer: Thank you for sharing that. And onto the next theme is about in-clinic experiences. So, I want you to walk me through your experiences when you’ve gone to the eye clinic. Have they been positive? Have they been negative? What’s made them positive? What’s made them negative?

EN9004: Are you asking me the doctor’s visit when I go in? Interviewer: Yes.

EN9004: Or when I see the doctor?

Interviewer: Both; inside the clinic, when you see the doctor, yes.

Everybody’s getting called in except me. So, I said, “What’s wrong? Why am I not called?” “Well,

another room to be seen.” I said, “Fine.” So, I’m sitting out there waiting, waiting, waiting.

and the time was going, going, going. And they brought me in and said, “You’ve got to be waiting in

day, I was there and I was waiting and I have [00:18:01]. So, I only had a limited amount of time

[redacted], because I don’t know them, sometimes they can be a little rude-ish. Not rude. Like the other

Dr. [redacted] were always great. Always, always great. But it seems like the nurses I get with Dr.

want special attention. I just want to be treated like I’m a person. Okay, at first, the nurses I had with

Okay. That’s, you know, at first, and then, second. When I went there - well, I don’t

In-clinic Experiences

EN9004:

Interviewer: Yeah, I’m sorry.

somebody else can come in the room.” And I thought that rather rude.

bring me in when Dr. [redacted] was done.” And she said, “Oh, if you don’t want to be in the room,

the room. And I said, “Sweetie, why do you bring me in? Because they told me that they’re going to

So, another nurse came - the one was gone, that one, and another one came back and she brought me in

I can’t be seen, I’ve got to come another day and I don’t really want to do that.”

to see me.” You know, he was going to do an injection in my eye that day but he didn’t. So, I said, “But

there’s a lot of people ahead of you but I see where you are.” And I said, “I’m sure Dr. [redacted] wants

EN9004: So, I don’t even know what else happened but I ended up being - then, I talked - I said something, I have no idea, it wasn’t - just let her know I wasn’t saying that. So, then, she said - I said, “I don’t know,” she said, “Somebody be in soon.” So, the doctor came in who works with - I don’t know whatever the…

Interviewer: Residents? The residents or the [interruption]?

EN9004:

Interviewer: Yeah, yeah.

the way they talk to you sometimes.

with Dr. [redacted] were great. But on the other side, they’re not. You know, they’re a little iffy. It’s

in Dr. [redacted]’s side, you be like, “Huh?” Because it was just most of them are - all the nurses I had

But my point was why Dr. [redacted]’s nurses always seem - or that side are always nice. And you go

and said what he said.

forget. The older lady, she was really nice. And she looked in my eye and then, Dr. [redacted] came in

Residents, right, came in and she did my eye. And she looked - or he, whatever it was, I

Competing Concerns

Cues to Action

Vision Status

Knowledge-creating Experiences

In-clinic Experiences

Coding Density

EN9004: And you don’t expect to be [00:20:12] like you’re the only one there but you wanted to be treated as a person. So, that’s the only problem I had.

Otherwise, I have always - normally, I just go with Dr. [redacted]’s office, come right out, not too long. But that day, I got - what time was my appointment? My appointment was at 2:30. I got there around ten after two and I think I got in the room about 3:00, [redacted] brought me back, because I had to

leave by 4:30.

you don’t get picked up, they won’t come out there and get you. They won’t come back. If they leave

So, I think they - I’m not sure what time but I was able to leave by 4:30 and I was - if

Interviewer: Right, you’re on a schedule.

room, I’m like, “What’s going on?” You know. So, I said, “Well, if I can’t be seeing him, I have to tell

whatever, “A lot of people ahead of you,” that was one person. And there’s the other person in the

Yes, for the ride home, right. So, that’s all I explained to them when she said, “Well,”

going by.

you, I’m saying. So, I was just a little afraid I was going to get left. Because the time was ticking, it’s

Resource Availability

| EN9004: |  |
| --- | --- |
|  |  |
|  | |
|  | |

Interviewer: Yeah. Thank you for sharing that experience. And I want to go to what you said about not feeling like a person. And when you have those types of experiences, you know, it sounds like you always go to your eye appointments anyway. But does that make you not want to go to the next appointment?

calm talking to her in the office, in the room.

sometimes I can be a little curt; sometimes be a little out of the way. So, that’s why I was being really

Oh, no. Oh, no. Because you know what? I try to be civil with my mouth because

me, he saw me. And I’m going to see him again the 30th, maybe get a needle in my eye, who knows?

the doctor that I’m going to have to go.” They didn’t tell him, they didn’t tell him, no. Yeah, but he saw

Emotional Context

EN9004:

Interviewer: I see. So, at this point for you, you have that priority and even if the experience isn’t so positive, you’re still going to make it to that point.

going to never stop me from going to the eye doctor, no.

have to go somebody’s - or whoever and explain to them that they’re doing that to me. No, it’s not

So, no, I’m going to never not go but I’m going to have to go pretty - if they do it again, I’m going to

EN9004: Oh, yes.

Interviewer: Okay. And why is that?

EN9004:

Interviewer:

Very much.

EN9004:

And do you trust them as your doctors?

If I can’t go to them, I’ll pay for it myself,” you know? I just like Dr. [redacted] and Dr. [redacted].

were, “I want them. I do not want to change where I go for my eye doctor. I don’t want to do nothing.

doctors? I love Dr. [redacted] and Dr. [redacted], I really do. And I told whoever my insurance people

happens to my eye. I do. So, why would I not go for an eye doctor’s appointment where I love the

My eyes, my eyes. That’s my eyes, that’s not their eye. They don’t care about what

Competing Concerns

Cues to Action

Resource Availability

Vision Status

Knowledge-creating Experiences

Emotional Context

In-clinic Experiences

Coding Density

Interviewer: Okay. EN9004: Very much.

Interviewer: And did you have to - did that trust build over many years?

EN9004: Well, with Dr. [redacted], I found her right away. And I had to build it with Dr. [redacted] because at first, I thought he was a chauvinist but I learned to love him.

Interviewer: [Laugh] Yeah. No, they’re both great. Like I said, I work with both of them. So, they both have their own different style but they’re great.

EN9004: Right. Dr. [redacted] usually is. He’s down to earth with me now, you know? He’s okay, he’s alright.

Interviewer: I see.

EN9004: But at first? Uh-uh. Interviewer:

Never.

EN9004:

But at this point, you want to see them. Like you don’t want to change.

Interviewer: Okay, okay. So, it’s good to hear about those - again, I’m sorry [interruption]…

Friday and someone came and took her place. I’m like, “Who is this? I don’t want to see them. Why

I’m going to say this - excuse me - I’ll say this. One day, Dr. [redacted] was gone on a

EN9004:

Interviewer: I see, I see. EN9004: Don’t have it. Interviewer: Yes.

was. But because if it’s not Dr. [redacted], don’t have that appointment for me.

am I here if she’s not here?” [laughter] I think - I don’t know what I did. I was really snotty; I know I

EN9004: But she was not there; it was on a Friday. She just started doing her stuff in on [redacted] and they were - she said, “I won’t be in no more Fridays.” So, they came - I didn’t know - excuse me.

Interviewer: No worries.

because I didn’t want nobody else. I didn’t want nobody else. No, no, I’m not going to see them, no.

I did know ahead of time, she did tell me. But I said, “I’ll come on Thursday, then,”

Competing Concerns

Cues to Action

Vision Status

Knowledge-creating Experiences

Coding Density

EN9004:

Interviewer: Okay, okay. Thank you, EN9004, for sharing that experience. We’re getting to the last two themes. Again, you’re covering ground beautifully. We’re kind of ticking off all the boxes. And if you want to share anything else, the floor is always open for you.

EN9004: Okay, like I told you, I’m good to talk.

Emotional Context

Interviewer: [Laugh] Yes. So, I mean, you kind of mentioned this before, especially during that period of seven/eight years where you weren’t seeing an eye doctor. You didn’t say that you were busy during that time or things were kind of…

EN9004: No, no, no, no.

Interviewer: … pulling at your attention. Okay. EN9004: No different than now.

Interviewer: Yeah.

appointment. I had no car; couldn’t drive because I couldn’t see - an eye doctor appointment to see Dr.

You know, I even used to take the bus - okay, I was at work and had an eye doctor

EN9004:

Interviewer: Oh, wow.

going in the other [00:26:21].” They all said, “Oh, yes, you are.” So, I went and I had a stroke.

look right, and so, she called for the ambulance, told me, “Go to the hospital.” And I said, “I’m not

[redacted] that day and that particular day in 2018, I saw her. She told me I didn’t sound right, I didn’t

Resource Availability

In-clinic Experiences

EN9004: I had a stroke at working. So, I took the bus to the eye doctor, I had a stroke, didn’t know it. She knew it; she knew something was wrong. And that’s why, you know, [00:26:40] asked me, I have no idea.

Interviewer: Yeah. So, I mean, but that will - I guess it says something about how you’ll be focused on things, you know, how you have focus on places you need to go, appointments you need to go to.

EN9004: Right.

Interviewer: And sometimes things happen out of your control.

knows me and, you know, she knows me; how I talk, how I act, you know. People at work didn’t even

Yeah. I’m just saying she’s so good. She knows me, I’m saying from 2015 to 2018, she

EN9004:

Interviewer: Wow. So, it’s good you went to your appointment that day because otherwise…

downtown. It was weird, too. It was so weird. How can I have a stroke and not even know it?

notice it. I’m at work and I was talking. Ain’t one of them say nothing. And I took the bus to the clinic

EN9004: Yeah, that’s what I’m saying.

Cues to Action

Knowledge-creating Experiences

Emotional Context

In-clinic Experiences

Coding Density

Interviewer: … you wouldn’t - no one would’ve noticed anything. EN9004: No, and it might not have been good for me.

Interviewer: Yeah, yeah.

EN9004: I couldn’t even walk, sweetie, after - after I got to the emergency room, I couldn’t walk anymore. I didn’t walk for - I went to the hospital, I went to rehab, and I had to learn to walk all over again. And I was good because I got to the clinic, was able to get there, you know what I mean?

Interviewer: Yeah. I mean, it’s a miracle. It’s a miracle you were even able to get to the clinic from work.

EN9004: Yes.

Interviewer: Wow. Well, I’m glad that things turned out the way it did. You know, no one wants to have a stroke but at least that you were able to get to the ED when you did go.

EN9004: Right.

Interviewer: Yeah, yeah. And I also wanted to - again, I think you pretty much covered these last two themes just while we were talking. But I had a question about - so, you mentioned you took the bus to go to your appointment. Are you currently working now?

EN9004: No.

Interviewer: Okay, and so, you’re [interruption]…

asked for another extension; they didn’t give it to me and I didn’t know it. They said they reached me

problem, I couldn’t see. I had to get time out on work. And then, they gave me FMLA. And then, I

Because my eye problem, I had an eye

Resource Availability

Vision Status

EN9004: They put me [00:28:48] really quick.

Interviewer: Wow, was time from [overtalking] work.

again, on Saturday, knocking on my door, saying resign by so-and-so date and the day had passed.

what I’m doing yet. We’ll let you know. Monday, they make a letter that I don’t know about. Then

and see them. Thursday, they gave me a letter; Friday, you’ve got to come in. Friday, I don’t know

but they never did. And they called me in one day with a letter; the next day, they wanted me to come

EN9004: Then, they sent that letter on Saturday; they put in for my resignation because I didn’t - I ignored my job. I didn’t ignore anything so, why did they put me out? They put me out. I got put out in 2019.

Competing Concerns

Interviewer: Wow. And this was after - so, before that, had work ever prevented you from going to your eye exam?

Did you find it hard to get time off?

Interviewer: I see, I see. And did your job require you to do a lot of like looking or seeing? EN9004: It was school; it was teaching.

appointment, I cancel whatever if I have to. But I will not cancel eye appointments.

up or somebody work in the store, whatever. No, never. I always made my eye - I came to my toe

No, never, never. If there was snow on the ground, whatever, I’ll get my ride to pick me

EN9004:

Competing Concerns

Cues to Action

Resource Availability

Vision Status

Knowledge-creating Experiences

Emotional Context

In-clinic Experiences

Coding Density

Interviewer: Oh, okay. What grade did you teach? EN9004: Elementary school.

Interviewer: Wow, wow. That’s amazing. I see. Well, again [interruption]…

EN9004: Well, no, it’s not elementary school; it was middle school. You know, I keep forgetting. Interviewer: Yeah, they kind of [overtalking] blend.

EN9004: You know, all of it now to eighth grade.

Interviewer: Yeah. Well, they blend into each other. I always say my elementary school/middle school teachers; I owe them everything for my foundation. It’s an underappreciated job, I’ll say that.

EN9004: Thank you. Interviewer: Yeah, yes.

EN9004: People don’t know what the kids are like. They may have them at home; they have no idea. So, a mother actually came in school one day. This boy is bad as a demon who lives in H-E-L-L. But his mother came in and said, “I know my son didn’t do that,” I just walked away. I walked away. “I know my son didn’t do that.” She don’t ever see him do it. He’s good at home.

Interviewer: At home, though. EN9004: In school, he was terrible.

Interviewer: Wow. Yeah, it’s a lot of patience.

EN9004: No, I didn’t have patience but I made myself have patience for myself. Because I didn’t let them kids get away with nothing - nothing.

Interviewer: Wow.

EN9004: And I went to the school to see them graduate and the janitor even said it’s not the same since I left. And he said whenever the kids saw me, they were, “Here go EN9004 again, here she comes.” I started cracking up. I ain’t known nobody noticed that.

Interviewer: Wow. Oh, my goodness.

EN9004: I was a good teacher but I was kind of too strict. But I had to be.

Competing Concerns

Cues to Action

Resource Availability

Vision Status

Knowledge-creating Experiences

Emotional Context

In-clinic Experiences

Coding Density

Interviewer: You had to, yeah. When I look back, it’s the strict teachers who I appreciate, too, because they knew what had to get done and, you know, the agenda.

EN9004: Yes. The ones who do something like [00:32:29], they run all over. They run all over them.

Interviewer: Yes, and the kids can be vicious. EN9004: Yes.

Interviewer: They know; they’ll see the weakness and they’ll… EN9004: Yes.

Interviewer: … take it.

EN9004: You know, you went to school. I went to school, too. Interviewer: So, you know, wow.

EN9004: You know, can I ask a personal question? Interviewer: Of course.

EN9004: How old are you? Interviewer: I am 26.

EN9004: You’re a baby. Interviewer: [Laugh]

EN9004: Yeah, I know you’re not - you know, at this time in life, 26 is old and it used to be young. But I’m saying to me, you’re young. But I know now that 30 is ancient to all of you.

Interviewer: No, 30’s the new 20. Everything [interruption]…

EN9004: You know, I heard a lady say, “I’m 30; I’m old.” She was serious.

Interviewer: No, it’s true. We all get that perspective and then, we reach later life and then, we realize how young we were.

EN9004: Right.

Interviewer: Yes, yes. Well, EN9004, luckily, you covered all the themes. I just wanted to, again, open the floor to ask if you had anything else you wanted to share or questions about the study.

EN9004: Well, you know, I just want to know where you’re going to be when you start opening up. You’re going to be at the [redacted]?

Competing Concerns

Cues to Action

Resource Availability

Vision Status

Knowledge-creating Experiences

Emotional Context

In-clinic Experiences

Coding Density

Interviewer: When I start - oh, when I start being a resident?

EN9004: When you start doing what you’re doing now. When Dr. [redacted] starts the research she’s doing, where’s it going to be held?

Interviewer: So, for the research that we’re doing, we’re still in the interview stage but we’re going to end up writing a paper. And then, once we write that paper, hopefully, it’ll get published. So, if you’re interested in seeing that published paper, we can send it along to you.

EN9004: Oh.

Interviewer: Again, it won’t have your name or anything. It’ll just talk about the themes and just the conversations. So, everything will be…

EN9004: I’m good to be by myself.

Interviewer: So, maybe you might see something like, “Hey, that’s what I said,” or, “That’s what - I said something to that effect.” But we won’t like do things verbatim or something.

EN9004: Right, right.

Interviewer: Yeah, we will protect your own identity. EN9004: I understand.

Interviewer: Yeah. So, we’ll have that and then, we’ll try to present it at conferences, too. So, again, if you’re interested in participating in more studies or learning about like where the project goes from here, I can keep you in the loop.

EN9004: Okay.

Interviewer: Yeah. And I wanted to talk to you about this other diabetes study. So, I have to ask you this question to see if you’re eligible because you’re over the age of 18, you have type 2 diabetes. Are you on the waiting list for rental assistance?

EN9004: No.

Interviewer: Okay. So, that’s one of the criteria. So, this is just, again, one study that’s going on. But when we have that [interruption]…

EN9004: And that’s for the people in that category?

Interviewer: Yeah, it’s people in that category. So, you’re not eligible for this one but we’ll have more studies coming around. So, we have your information; we can always keep you in the loop. I have your address so, we’ll try and get that gift card mailed to you this week, too, yeah.

But again, EN9004, I want to thank you for your morning and thank you for being so candid about your experience.

Competing Concerns

Cues to Action

Resource Availability

Knowledge-creating Experiences

In-clinic Experiences

Coding Density

EN9004: You’re so welcome.

Interviewer: Yeah, I really appreciate it. I know this is going to help us just improve eye care for all people with diabetes because that’s our goal.

EN9004: Right. I figured, you know, that - that’s why I said you need to tell somebody about what I - you know, how my eye started seeing things and you know.

Interviewer: Yes, yes.

EN9004: And people are out there driving their cars knowing they cannot see. Interviewer: Yes, yes.

EN9004: I stopped driving my car in 2019 because I know that why you out here?

You know you

Vision Status

. You’re going to hit somebody, you’re going to whatever. They’re out there and they are

can’t see

driving.

Interviewer: Yeah, it’s hard, especially people - say, with truck drivers and stuff like this happened. I think, again, they won’t be able to drive after it but it’s hard to see how people balance their vision with something as daily as driving.

EN9004: Yes.

Interviewer: Yeah. No, I agree.

got to depend on a bus or whatever to get where you want to go. I usually go to [redacted] - [redacted],

I hated it when I had to stop driving. You drive here, you drive there. Well, now you’ve

EN9004:

Interviewer: Yeah. It’s your independence and you have to… EN9004: My phone is talking; I can’t hear you.

to [redacted]. I want to go to [redacted] now.”

[redacted], you know, drive in and wherever. But now, you can’t get on the bus and say, “I want to go

Emotional Context

Interviewer: Oh, sorry. I was saying it’s your independence so, it kind of messes with it a lot, yeah. EN9004: Right, right.

Interviewer: Again, thank you so much, EN9004, and hopefully… EN9004: You’re so, so welcome.

Interviewer: … hopefully, we’ll be in touch. But I hope you enjoy the rest of your holiday weekend - long weekend.

EN9004:

Interviewer:

No, again, it was wonderful to talk to you so, take care.

EN9004:

Okay, thank you so much.

Interviewer:

Bye, EN9004.

***End of formal interview***

Competing Concerns

Cues to Action Resource Availability

Vision Status

Knowledge-creating Experiences

Emotional Context

In-clinic Experiences

Coding Density

You too, you too. [00:37:14] talking to me but enjoy the rest of the day.

Interviewer: I wanted to make sure I had your consent to record and participate in this study?

Vision Status

In-clinic Experiences

Cues to Action

Coding Density

EN9005: Yes, you do.

Interviewer: Okay, perfect. Now, we’ll move on. The purpose of this study is to ask about your experiences getting an eye exam as a person with diabetes. I know it’s been a couple years since you’ve gotten your eye exam, but we value your input because we want your honest thoughts and we think they’re invaluable. We know that you’re the expert of your own experiences. At times, I may ask you to elaborate or explain more about your answers. That doesn’t mean I don’t think they’re clear; I just want to hear more about them. There are no right or wrong answers. All your responses will be kept anonymous. Do you have any questions for me?

EN9005: Not yet.

Interviewer: Great. Again, as you have questions, we’ll continue to answer them. I’m going to go through themes. With each slide I’ll talk about themes that we’ve created after talking with other patients or other participants, and I want to hear your thoughts as to whether that speaks to you or you feel that resonates with you. It’s okay if it doesn’t. So we’ll go through it line by line. The first theme is called resource availability. Participants in the past have talked about having limited resources and not being able to go to their eye exams. I can you examples, but I wanted to ask openly have the lack of resources ever kept you from being able to go to an eye exam?

EN9005: Yeah.

Interviewer: Okay. For some examples, this can include insurance, not having money, not having transportation, a car or a bus to get to the eye exam, being incarcerated or not even having a home? Do any of those seem to be familiar to you?

EN9005: Nope. We’re good.

Interviewer: So what has stopped you from going to eye exams say in the past couple years?

EN9005: It’s not like something where I

Appointments, really. Mainly, it’s been me.

Emotional Context

Resource Availability

had reasons not to go.

Interviewer: Okay.

EN9005: Just me, just appointments.

Interviewer: Disappointments. Okay. Can you speak a little bit more about that. EN9005: No. No. Appointments. Not disappointments. Appointments.

Interviewer: Oh, appointments. I see. You mean you didn’t have any appointments to go to?

EN9005: Right.

I didn’t set an appointment up. I forgot.

Emotional Context

Vision Status

In-clinic Experiences

Cues to Action

Coding Density

Interviewer: You say that you think the reasons were more because of yourself. Why do you say that?

EN9005: Because I didn’t do the appointment. I just [00:03:43] the appointment. I didn’t set the appointment.

Interviewer: Yeah. Why do you think that is? EN9005: I don’t know. I forgot.

Interviewer: Yeah. That makes sense. I guess in the past when you had been able to go to eye appointments or any doctors appointments, has anything helped you to remember?

EN9005:

Yeah, what has helped me was it probably set for me and someone reminded

me.

Interviewer: I see.

EN9005: The question was has I, then it went on from there. Interviewer: Okay. Were those appointment reminders by phone or text? EN9005: Usually at my doctor’s appointments.

Interviewer: Okay. Do you have an eye doctor that you saw regularly? EN9005: Not necessarily regularly. I have a couple I’ve been to. Interviewer: Okay. What do you think got you to start going to an eye doctor? EN9005: Convenience.

Interviewer: Okay. Say more. EN9005:

real easy for me to get to.

That’s it. Convenience. [00:05:07] passing by or it’s been some place

Resource Availability

Interviewer: I see. Okay. So the eye doctor that you used to go to, were they close by? EN9005: Yeah.

Interviewer: How did you usually get to that eye doctor? EN9005:

Walk or bus.

Interviewer: Okay. I see. Has anyone in your life, whether that’s family members or friends, have they ever helped you to get to an eye appointment?

EN9005: No, not necessarily.

Emotional Context

Resource Availability

In-clinic Experiences

Coding Density

Interviewer: Okay. You mentioned convenience in terms of helping you get to eye appointments. What do you think motivated you to go to your eye appointments when you were diagnosed with diabetes?

EN9005: Convenience means I walk by and there was an eye place. That’s what convenience means. It just happened to be where I was.

Interviewer: I see. Okay. When you were diagnosed with diabetes, were you told that diabetes could affect your eyes?

EN9005: [00:06:24].

Interviewer: Sorry?

EN9005: [00:06:25].

Interviewer: Oh, [00:06:26]. Okay. What did you think of that? EN9005: I didn’t know it but [00:06:35].

Interviewer: Oh, sorry. Hello? Hello? EN9005? Hello, EN9005? Hello? EN9005: Can you hear me?

Interviewer: Yes, I can hear you. Sorry, I think I lost you for a little bit. EN9005: Yeah, I told you to hold on a second.

Interviewer: Oh, okay. Sorry about that.

EN9005: [00:07:07].

Interviewer: I wanted to know -- so you were told that diabetes could affect your eyes. Did you try to look for more information about that?

EN9005: [00:07:38].

Interviewer: Hello? Oh, no worries. No worries at all. I was saying that when you were told diabetes could affect your eyes, did you have an interest in looking up more about that or asking more questions?

EN9005: Well, it must have affected my sight [00:08:01] for me to want to act upon it [00:08:03].

Interviewer: Okay. Sorry, could you repeat that first part? EN9005:

checked _____

I said it must have affected my eyes at one point in order to have my eyes

[00:08:17].

Vision Status

Cues to Action

Interviewer: I see. Okay. Does anyone in your family or any of your friends have diabetes?

Emotional Context

Resource Availability

Coding Density

EN9005: Yeah. My family, yes.

Interviewer: Okay. Have any of them had experiences with their eyes or their vision? EN9005: I don’t know.

Interviewer: Okay. No, that makes sense. So it sounds like you were told that diabetes could affect your eyes but I guess from the time you’ve been diagnosed the diabetes from now, how has your vision been?

EN9005: All right. I just wear readers. Interviewer:

Okay. Have you had any big changes to your vision?

Vision Status

EN9005: No.

Interviewer: Okay. Any blurry vision?

EN9005: No.

Interviewer: Okay. So it sounds like your vision’s been pretty stable since you were diagnosed with diabetes.

EN9005: Yep.

Interviewer: Okay. That’s good to hear. I guess going onto another theme, and again, we’re kind of going through the themes as I talk to you. So thank you for answering these questions. I wanted to know how has your experience been when you’ve been in the clinic? When you’ve gone to an eye doctor appointment. Tell me about that experience.

EN9005:

recommendations and that was it. There’s nothing more than that.

convenient. I got my eyes checked. They checked my eyes. They did the

I just haven’t been to one in about two years and it had to be really

In-clinic Experiences

Interviewer: Okay. And do you remember if your eyes were dilated? EN9005: I don’t know.

Interviewer: Okay. That’s okay. So it seems like convenience is important to you. Has that stopped you from going to, say, other doctors appointments?

EN9005: No, not right now. I just haven’t remembered.

Interviewer: Okay. I see. Do you find that you need to trust [00:10:21] an eye appointment? What would motivate you to go to an eye appointment, say, in the next month?

Cues to Action

EN9005:

Convenience.

Interviewer: Hm-mmm. Anything else?

Emotional Context

Resource Availability

In-clinic Experiences

Cues to Action

Coding Density

EN9005: Nope.

Interviewer: Okay. I know you described convenience before as it being right next to you, being able to go. What else defines convenience for you?

EN9005: There’s nothing else that can define convenience for me than what I already told you.

Interviewer: Okay. No, that makes sense. Again, I thank you for sharing your experience and sharing, again, how it’s been since you’ve been diagnosed with diabetes. I wanted to know if you had questions about this study or questions about getting eye appointments in general?

EN9005: Not really. If I got to get eye appointments, then usually it’ll be something that motivates me too.

Just like we talked about, blurry vision or something

Vision Status

But otherwise, there’s nothing else that other than my doctor tells me it’s about that time to get my eyes checked.

like that.

Interviewer: I see. So you’re mentioning kind of changes in your own vision or if your doctor would tell you, oh, it’s time to get your eyes checked?

EN9005: Right.

Interviewer: Okay then. You mentioned that your last eye appointment was two years ago. When you were diagnosed with diabetes, had you seen the same eye doctor, or did you see different eye doctors?

EN9005: I hadn’t seen any eye doctors.

Interviewer: Oh, okay. So two years ago was your first and only eye appointment?

EN9005: No. No. Say like two years prior to that. That was when I couldn’t -- my eyesight was blurry. Something like that.

Interviewer: Okay. So how many eye doctors have you seen then since 2014? EN9005: One.

Interviewer: Okay. I see then. Thank you for clarifying that. I just wanted to make sure I had the timeline correctly. You mentioned that the last time you’d seen any doctor has been two years, so that includes like a general doctor?

EN9005: Yep.

Interviewer: Oh, okay. That makes sense. So I think we’re nearing the end of the questions. Again, you covered everything as we went so I appreciate that. Is there anything that you wanted to -- any experiences that you want to speak more about or talk about?

Emotional Context

Resource Availability

Vision Status

In-clinic Experiences

Cues to Action

Coding Density

| EN9005:  Interviewer: | No. No, not right now I don’t.  Okay. Okay then, EN9005. Thank you so much for giving me your morning. |
| --- | --- |
| EN9005: | Okay. |
| Interviewer: | Okay then. I have your address, so you should expect a gift card in the mail about in the next week or so. |
| EN9005: | Okay. |
| Interviewer: | Okay. Take care. |
| EN9005: | Thank you. |
| Interviewer: | All right. Bye. |
| EN9005: | Bye. |

***End of formal interview***

Interviewer: --asked them with me. And so, before we get started, I just wanted to get your verbal consent to participating in this study.

Vision Status

Cues to Action

In-clinic Experiences

Emotional Context

Coding Density

EN9006: Yes, ma’am.

Interviewer: Okay, great. So, the first thing that we have is called resources. And so, I can give you examples. But I wanted to hear from you as to if there have ever been any obstacles in getting eye exams.

EN9006: I’m going to say no. Interviewer: Okay.

EN9006:

have to be seeking it.

All we have to do was basically seek the information. It’s readily available. You just

Resource Availability

Knowledge-creating Experiences

Interviewer: I see. And has there ever been issues of say insurance, transportation? EN9006: No, ma’am.

Interviewer: Okay. So, the eye exams that you have been able to go to, have you ever cancelled an eye exam?

EN9006: No, ma’am.

Interviewer: Okay. Have you ever missed an eye doctor appointment or realized that you couldn’t go last minute?

EN9006: No, ma’am.

Interviewer: Okay. So, usually you’ve been able to get to your appointment fine? EN9006: Yes, ma’am.

Interviewer: Okay. And how do you usually travel to your doctor’s appointments? EN9006:
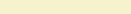


I have a vehicle.

Interviewer: Okay. So, have you ever had to rely on public transportation or--EN9006: Yes, ma’am.

Interviewer: And how was that experience versus say driving? EN9006:

A lot of to and from, and way overcrowded, obnoxious people on the city bus.

Interviewer: Okay. But it looks like that didn’t stop you from still going.

EN9006: No, because at that time I didn’t have an eye exam appointment. I didn’t do all those things with transportation.

Interviewer: I see. EN9006:

So, I keep them as current issues. They were priorities, so.

Cues to Action

In-clinic Experiences

Knowledge-creating Experiences

Emotional Context

Coding Density

Interviewer: I see. And I’m hearing you say that they were a priority.

EN9006: Yeah, to go to my eye appointments. All appointments were priorities. Interviewer: Yeah. And why are eye appointments a priority for you?

EN9006:

visit.

You need to because it’s bad enough I lose my mobility. I’m a diabetic. You need to

Resource Availability

Interviewer: Yeah. Yeah, no, it makes sense. And what got you to your first diabetic eye screen? EN9006:

My vision changed.

Vision Status

Interviewer: How did it change? EN9006:

I noticed that things that I used to could see were blurry.

Interviewer: And was this gradual or did you think it happened all at once? EN9006: No, at the time I was being diagnosed as diabetic.

Interviewer: And how did that make you feel? EN9006:
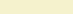


Insecure.

Interviewer: Say more. EN9006:

things you’re not familiar with, you know, lean on [redacted] things.

Just don’t have a good feeling about it, you know. Things that you’re familiar with or

Interviewer: Yeah. And you said lean on things. Do you mean physically? EN9006: Yeah, because of your mobility and your balance, you know.

Interviewer: Okay. So, it seems that those were some of your worries while your vision was changing.

EN9006: Correct.

Interviewer: Okay. Was there anything else going on at the time that made you say in your mind, “Okay. I have to go to my eye exam”?

EN9006: No.

Interviewer: And you mentioned 2017, that’s when you were told you had pre-diabetes or possibly diabetes. Do you remember having a conversation with your primary care doctor about how diabetes could affect you or your eyes?

EN9006:

become dependent on medication. You need to do something to change your

you eat. You have to watch what you do. Exercise is required. You don’t want to

then. It affects what you see. Diabetes is not good period. You have to watch what

Of course, that A1C level. We had to get it down below 6.5 and it was over 7 by

Vision Status

Resource Availability

In-clinic Experiences

Knowledge-creating Experiences

Emotional Context

Coding Density

Interviewer: Yeah, no, definitely. And or I guess tell me more about that conversation that you had with your primary care provider.

situation. So, you have to use physical exercise—walking, something.

EN9006: The conversation I had with the primary care provider I just told you. Interviewer: Okay.

EN9006: The conversation I had with myself--Interviewer: Oh.

EN9006: --was I told her, “I don’t want to take medications.” I remember when they told me I had to take insulin and I refused to take it. I never took it. And then, all of a sudden I changed my eating habits, and exercised, and she told me. “[redacted], don’t take the insulin.” I said, “I never have.”

Interviewer: Ah, I see. Okay.

EN9006: So, that worked out pretty good because for a whole year I didn’t take it.

Interviewer: Wow. Yeah, no. That sounds great. And so, I’m trying to see how to phrase this question. And so, after you were diagnosed with diabetes and you started changing your lifestyle, how did you keep track of some of the appointments that you had say with your eye exams?

EN9006:

My Chart.

Cues to Action

Interviewer: My Chart, okay. Have you been using My Chart for a while? EN9006:

how it works with all that. I’m not all that tech savvy.

It’s not on my phone now because when I changed phones I can’t get it. I don’t know

Interviewer: Oh okay. EN9006:

in there.

I do have the color note app though, so at any point in time I just immediately put it

Interviewer: Oh okay, the color note app? EN9006: That’s correct.

Interviewer: Where did you hear about this app?

EN9006: I discovered it on my own somehow. I don’t know and I ended up using it. I didn’t think it would be, but it’s a great app.

Interviewer: Okay. That’s great. And so, how many years have you been using it? EN9006: Just one.

Vision Status

Resource Availability

Cues to Action

In-clinic Experiences

Coding Density

Interviewer: Okay. But it works for you, so okay. That sounds great. Are there any other things you use to remind yourself of appointments or making sure you get to them?

EN9006: No, all you need is one thing.

Interviewer: Okay. No, that sounds good. And once you were diagnosed with diabetes or started going to eye exams, did you try to seek out more information about diabetes?

EN9006:

give you. A person with diabetes [00:07:14] as well. And they can read it.

Well, I had spoken to my primary care and then I read all the pamphlets that they

Knowledge-creating Experiences

Interviewer: Yeah. EN9006:

Reading’s fundamental.

Interviewer: Yeah, great. So, you’re a person who likes to read about--EN9006: I like to read period.

Interviewer: Okay. Did you feel any fear or--EN9006: No.

Interviewer:

your organs?

--or not? Okay. So, you wanted to know more about diabetes—how it could affect

Emotional Context

Interviewer: Okay. EN9006:

something more popular--

You’re already enjoying it even prior to being diagnosed. So, you have to consider

Especially if you feel like you can’t eat the things that you think you can, you know.

Absolutely.

EN9006:

Interviewer: Okay.

EN9006: --and stick to it. Interviewer: Okay.

EN9006: I go to the gym. I go to Planet Fitness. I, you know, I do yardwork. I cut the grass, everything. So, yeah. Just definitely hot out there. You sweat. So, I don’t know how you’re supposed to do that without getting a lot warmer.

Interviewer: Yeah, no, absolutely. And did you find a network either of friends or family with diabetes?

EN9006: No.

Interviewer: Okay. Do you have any family members who also have diabetes? EN9006: Not that I know of.

Vision Status

Resource Availability

Cues to Action

Coding Density

Interviewer: Okay. So, did you feel that you were kind of in the dark at first when trying to learn more about this?

EN9006:

Reading is fundamental, no. It’s not hard to find information about it.

Knowledge-creating Experiences

Emotional Context

Interviewer: Okay. No, that’s good to hear. And so, now I want to transition to your experiences in the clinic. So, I want you to try and picture say your last eye exam. Walk me through that experience and how you thought it was.

EN9006:

walk in the park.

technology, they’re very informative and I ask questions anyway. So, it was just a

You go in, they dilate your eyes. They look at your eyes through that thing. The new

In-clinic Experiences

Interviewer: You said a walk in the park? Sorry? Hello? EN9006: Yeah, I’m here.

Interviewer: Oh yeah, I just wanted to clarify. Did you say it was a walk in the park? EN9006: That’s correct.

Interviewer: Okay, that’s good. And so, you said that you usually ask questions. When you ask questions do you feel that you get answers that make sense to you?

EN9006: Absolutely.

Interviewer: Okay. Has there anything that has ever made those visits not so great? EN9006: No.

Interviewer: Okay. And for you, what makes say an eye doctor appointment like a positive experience for you?

EN9006: Your vision. Interviewer: Say more. EN9006:

It’s a part of your senses. You need it.

Interviewer: Okay. And so, this is one thing. Again, you mentioned changes in your vision and how that motivated you to go see eye exams. Sometimes other participants have mentioned that there’s this fear of the unknown or fear if they go to the doctor’s appointment they might find out what the reality is. Have you ever felt that way?

EN9006: No.

Interviewer: Okay. And compared to say your vision when you were diagnosed with pre-diabetes, how’s your vision now?

Vision Status

Resource Availability

Cues to Action

Coding Density

EN9006:

have to stay on top of it.

primary care, you know. Your vision changes as you exercise, you know. You just

Oh, well, what’s the A1C now compared to when it was recommended by your

Emotional Context

Interviewer: Yeah. And do you have a particular eye doctor or do you tend to see different people?

EN9006:

No, I go wherever he is.

In-clinic Experiences

Interviewer: Okay. And have you seen the same person--EN9006: Yes.

Interviewer: --multiple times? EN9006: Yes.

Interviewer: Okay. And how was that experience with that particular eye doctor? EN9006:
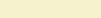


Oh, it’s fine.

Interviewer: Do you feel that you can trust your eye doctor? EN9006:
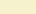


Yes.

Interviewer: Okay. Why is that? EN9006:

walk you through it. So, there isn’t any mystique to it.

They’re very informative. They tell you about whatever you needed prior to and they

Knowledge-creating Experiences

Interviewer: Sorry. You said there isn’t any? EN9006: Mystique to it.

Interviewer: Okay, mystique to it. Right. Okay. And I think I had mentioned this a little bit earlier, but I wanted to ask too if there’ve ever been any competing concerns when trying to get an eye appointment. So, some examples include say you have a job during the day. Say you have to take care of childcare or you were in the hospital and weren’t able to make your appointments. Has there ever been anything that has been conflicting with your ability to go to eye appointments?

EN9006: No.

Interviewer: Okay. So, are you currently working? EN9006: No.

Interviewer: Okay. Did you ever work a full-time job?

EN9006: Actually, I’m an amputee, ma’am. Interviewer: Sir?

Vision Status

Resource Availability

Cues to Action

In-clinic Experiences

Knowledge-creating Experiences

Emotional Context

Coding Density

EN9006: I’m an amputee. Interviewer: Oh okay.

EN9006: I have neuropathy in my left leg.

Interviewer: I see. I see, okay. I'm sorry to hear that. So, in your case, you’ve been able to make appointments without concerns?

EN9006: Absolutely.

Interviewer: Okay. Thank you for clarifying. And have you ever had any emergencies with your vision?

EN9006: No.

Interviewer: Okay. Okay, and again, I think we breached most of the topics. Again, thank you for elaborating on a lot of these topics. I think we’ve been able to go through a lot of the main things. But I wanted to open up the floor to see if there was anything that you wanted to add.

EN9006: No, ma’am.

Interviewer: Okay, yeah. And any questions about again, this study or just eye exams in general. EN9006: No, ma’am.

Interviewer: Okay.

EN9006: I’ve done a couple studies. I like the study. Interviewer: Do you?

EN9006: I like how you learn through the study and the questionnaire as well. Interviewer: No, absolutely.

EN9006: If you have any other studies, put me in. Interviewer: Yes, of course.

***End of formal interview***

---- START OF INTERVIEW---

Cues to Action

Competing Concerns

In-clinic Experiences

Vision Status

Knowledge-creating Experiences

Emotional Context

Coding Density

Interviewer: Yeah. Do you have any questions before we start? EN9007: Not at all.

Interviewer: Okay. And I want to know if you consent to this study. EN9007: Yes, I do.

Interviewer: Okay, great. So I'm going to go through themes, and that's how I'll kind of guide the talk that we're having. Our first theme is about resources. So I just wanted to ask, have there ever been kind of barriers to getting eye exams for you?

EN9007: Meaning? Interviewer: Meaning, so--

EN9007: Is it a hard time getting an appointment?

Interviewer: Yeah. So some examples may be not having insurance, not having money to fund the appointment, not having transportation, like not having a ride to the appointment.

Interviewer: So which ones seem to resonate with you?

Yeah, so I would say yes.

EN9007:

Resource Availability

Interviewer: Yes, say more. Oh, [redacted]? EN9007: Hello?

Not really having transportation.

EN9007:

Interviewer: Hello, can you hear me? EN9007: Yeah, I can hear you.

Interviewer: Oh yeah, I was asking you to say more. What do you mean about transportation? How do you--

I'm in so much pain I can't even ride a bus, young lady.

The bus. I would take the city transportation when I was able to get on a bus. Right now,

EN9007:

appointments now?

Interviewer: So how did you get to appointments before COVID, and how do you get to

up for this appointment. So I couldn't make it there.

insurance. Before, they didn't have that. So my funds wouldn't last me on different days I had to show

EN9007: Well, they got now-- they have, because of COVID, I can get a ride now through my

Interviewer: Oh, I'm sorry to hear that. EN9007: It's okay.

Interviewer: Okay. You said it's Video?

Veyo, it's called, now.

EN9007:

Interviewer: Yes.

Yes.

EN9007:

Interviewer: And so you use a ride share service now?

Cues to Action

Competing Concerns

In-clinic Experiences

Vision Status

Knowledge-creating Experiences

Resource Availability

Coding Density

Interviewer: I see. So sometimes it's just the setting it up and making sure people come at the right time.

appointments.

driver don't know where, half the time, that I am right now. And it caused me to miss out on some

And they say-- I call them like I'm talking to you now and set it up with them on the phone. Why the

EN9007: Well, they've been messing up on different dates that I have to be at an appointment.

Interviewer: Okay, Veyo. And how has that experience been?

Veyo. That's the name of the transportation department now.

EN9007:

EN9007: Right, right.

Interviewer: I see. And has anyone ever driven you to doctor's appointments? EN9007: In the past, yes.

Interviewer: And I guess now sometimes you can't get that arranged? EN9007: No, because everybody's working that I can get a ride from.

I'm sorry to hear that. How have you balanced not having housing with going to

Interviewer:

I am definitely homeless.

Yes, yes, yes, yes. I'm homeless as we speak but I have somewhere to be at for now. But

EN9007:

appointments?

EN9007:

incarcerated.

again, and other examples have been not having secure housing, so being homeless or being

Interviewer: Okay. So it seems like transportation definitely resonates with you. I wanted to know--

Emotional Context

It's difficult, but I manage.

Interviewer: I'm sorry about that bad experience. EN9007: Thank you.

they were saying I'm aggressive and all of that. I don't got time for that.

before. All of my belongings was taken away, stolen. And I addressed the problem. If I got too upset

I refuse. I refuse to go in anybody's shelter because I had a bad experience there once

EN9007:

members?

Interviewer: Have there been any tools that have helped you, whether that's shelters or family

Cues to Action

Competing Concerns

In-clinic Experiences

Vision Status

Knowledge-creating Experiences

Emotional Context

Resource Availability

Coding Density

Interviewer: Yes. So now, I guess, being able to find family or friends, does that help? EN9007: Yes.

Interviewer: Okay, okay. So thank you, [redacted], again, sharing that experience. I know it's not easy to share that. And I admire you for still being able to balance your health, even with the situations around you are not great. And then, another example that we've had when we've talked to other patients have been even incarceration. So sometimes in prisons or in jails you can't get routine eye exams or routine doctors appointments.

EN9007: Right, that's right. Interviewer: So I wanted to know--

EN9007: I just thank God I ain't never had to go incarceration and do this.

Interviewer: So that's good to hear. So that doesn't necessarily resonate with you, but you kind of see how that would be difficult.

EN9007: Yes, I can imagine.

Interviewer: Yes, okay. Again, thank you, [redacted], for sharing about that first theme. Is there anything else you want to talk about in terms of lack of resources that have affected you?

EN9007: You said lack of resources?

Interviewer: Yeah, not having resources, and that's why you haven't been able to go to doctor's appointments.

bring me, I would have been there. And then I have to go through stress of asking somebody to bring

EN9007: Well, just I wasn't able to get there, that's all. If I would have had somebody to come

Interviewer: Okay. And that's happened to you before, where it's kind of the burden of asking a favor. EN9007: Exactly.

want to go through that. That's why I didn't make it.

you, have to deal with they attitude and whatever they was supposed to be doing at the time. I don't

Interviewer: I see. No, that makes sense. Again, thank you for talking about that. So the second theme I have, it's called Cues to Action. And basically-- you already mentioned this, actually, with MyChart helping you a lot. So I wanted to--

Oh yes. Yes, it has.

EN9007:

Interviewer: Yes. So it seems like it's been very helpful for you.

Yes, I mentioned MyChart, I know that.

EN9007:

Cues to Action

Competing Concerns

In-clinic Experiences

Vision Status

Emotional Context

Resource Availability

Coding Density

Interviewer: Wow, okay. Yes.

2012. They operated on me. And then I had a kidney removed, just this May past.

I've got all my images on there, because you know I had surgery. I had a brain tumor in

EN9007:

Interviewer: Yes. So I wanted--

EN9007: And I see all that on my phone through MyChart. And now I can't see nothing.

Oh, for a while, while. Over three, four years, if not more.

EN9007:

Interviewer: I see. And how long have you been using MyChart?

EN9007: Thank you.

Interviewer: That's great to hear.

Interviewer: Yeah. I wanted to ask, do you remember when you were diagnosed with diabetes? EN9007: 2012, right after I had my surgery.

No. If I did, I don't remember, young lady.

I think I was having like a lot of sweats at the time, and I was told that I had a lot of

EN9007:

Interviewer: Were you told anything else?

sugar.

EN9007:

Interviewer: Okay. And do you remember what that experience was like?

Knowledge-creating Experiences

Interviewer: Okay. And how did you learn that? EN9007: Through my doctors.

all of that.

Oh yes. Your eyes, your limbs, your hands, your fingers. Amputations. I already know

EN9007:

wanted to ask if you were ever told that diabetes could affect your eyes.

Interviewer: Yeah, no, that's okay. That was a while ago. So that's more than understandable. I

Interviewer: Yeah. And do you remember getting your first eye appointment? EN9007: No, I do not.

It was scary. Very scary. I hope I'm not one of those.

EN9007:

Interviewer: Yes. And what did you think of that information at the time?

Cues to Action

Competing Concerns

Knowledge-creating Experiences

Emotional Context

Coding Density

Interviewer: Okay. Have you ever seen an eye doctor? EN9007: Yes, I have.

they drop these drops in your eyes to look at your pupil, if I'm saying it right?

they were seeing my eyes for. I really don't remember. But I know they are giving me dilation, when

I forget. I think some type of cataracts. They say, I believe-- I'm not even for sure what

Interviewer: Yeah, pupil, yep.

EN9007:

Interviewer: Okay. What did you see the eye doctor for?

In-clinic Experiences

Vision Status

EN9007: Yes.

Interviewer: On [redacted], okay. So you were saying that's the last time you went.

that's the last time I went to the eye doctor, on [redacted].

Yep. But I know I'm in the computer. And I used to go down on [redacted]. Actually,

EN9007:

Interviewer: And you said that you had kind of learned about cataracts too. EN9007: Yes.

Interviewer: Do you remember anything else about that experience? EN9007: No, I do not, young lady, I'm sorry.

Interviewer: What do you think has prevented you from going to eye appointments every year?

Say again?

EN9007:

every year?

Interviewer: Yeah. And so what do you think has prevented you from going to eye appointments

Oh yeah, I think of it as a positive, because they're trying to help keep my eyes strong

and safe.

EN9007:

experience?

Interviewer: No, that's okay. I guess, overall, do you think of it as a positive experience or a negative

Resource Availability

Interviewer: Yeah. Anything else? EN9007: No, just transportation.

Again, I would say transportation.

EN9007:

Cues to Action

Competing Concerns

In-clinic Experiences

Vision Status

Resource Availability

Coding Density

Interviewer: Yeah, transportation. No, perfect. Again, and as you share more, you're answering a lot of the questions that I already had lined up, so you're doing a great job, [redacted], and I appreciate it.

EN9007: Thank you so much.

Interviewer: Yeah. So I wanted to go onto a next theme, because you talked about how you learned that diabetes could affect your kidneys, your limbs, your eyes, and that the information was scary, which, again, it is, when you hear about a disease that can affect so many organs. So when you were given this information, did you do your own research? Or did you have ways of finding out more information?

Interviewer: Thank you, [redacted], for sharing that about your father. I'm sorry to hear that for him--

Now he's in Heaven. I don't want to go through that.

Yeah, I'm just trying not to do what he did, which is go on dialysis and he soon gave up and gave in.

him, he had to do dialysis, at the end of the day. I'm trying not to do that. How could I explain this?

EN9007: Well, I go back-- because my granddad had diabetes. And what ended up happening to

Knowledge-creating Experiences

Emotional Context

EN9007: No.

anyone in your family who went blind from diabetes.

Interviewer: Oh, your granddad, yes, thank you for correcting me. And I wanted to know if you knew

EN9007: My granddad.

Interviewer: Okay.

EN9007: Nope, I don't. Nobody in my family went blind from diabetes. Interviewer: Okay. And has anyone else in your family had diabetes?

an aunt, recently, as far as last week, from complications with diabetes. Yep, all her organs collapsed

Not that I can think of. I know, on my father's side, they have a lot of diabetes. I just lost

EN9007:

Interviewer: I'm sorry, [redacted].

on her and everything. Yes.

EN9007: That's okay. She's in a better place than here, I can tell you that.

Interviewer: Yes, no, absolutely. And I wanted to ask you, have you ever felt-- I guess, going back to experiences with your doctors, have you ever had any negative experiences with either eye doctors or other doctors?

EN9007: No. Only thing I did, when they did my kidney, I asked them to let me see it and they didn't.

Cues to Action

Competing Concerns

Vision Status

Knowledge-creating Experiences

Resource Availability

Coding Density

Interviewer: Oh, okay.

EN9007: And I was heated about that after I got out of surgery. Interviewer: Oh. You wanted to see it?

EN9007: Yes, I did.

Yes, pretty much.

EN9007:

Interviewer: Yeah. I see. And do you feel that you can trust your doctors?

In-clinic Experiences

Emotional Context

Interviewer: Okay. And have they talked to you about getting eye exams?

Yes, I have.

EN9007:

Interviewer: Okay. And I guess have you seen your primary care doctor recently?

EN9007: Yeah, she was asking if I had it and I had one set up and I just don't remember when.

Interviewer: Okay. So has your primary care doctor helped you to keep track of other appointments, or remind you?

EN9007: Say again?

Interviewer: Has your primary care doctor helped you with reminders? EN9007: No, because I haven't talked to her like [00:18:15]--Interviewer: Oh, hello, [redacted]?

EN9007: [00:18:25]

Interviewer: Oh, [redacted], it seems like you're cutting out just a little bit. EN9007: Hold on one minute.

Interviewer: Okay, now you're back. EN9007: Huh?

Interviewer: Now I can hear you okay. EN9007: Oh, okay.

Interviewer: Yeah, you're perfect. You were talking about your primary care doctor.

EN9007: [00:18:51]

Cues to Action

Knowledge-creating Experiences

Emotional Context

Resource Availability

Coding Density

Interviewer: Hello?

EN9007: Since the COVID. [00:19:04] she's concerned about her patients. Interviewer: Oh, hello, [redacted]?

EN9007: Could you hear me?

Interviewer: Oh, now I can hear you. Sorry, beforehand, it cut out again. EN9007: Hold on one second.

Interviewer: No worries. EN9007: Okay, I'm here.

Pretty good. It's been pretty good, I guess.

EN9007:

diagnosed with diabetes.

Interviewer: Okay, that's good to hear. I wanted to ask how your vision has been since being

Yes. Very nice lady. I like her.

EN9007:

Interviewer: Okay, perfect. You were talking about your primary care doctor.

In-clinic Experiences

Vision Status

EN9007: Yes.

_ [00:20:16] just for medications that I'm taking. I've had this blurriness.

I wouldn't say double vision. What I'm saying, there's like two of some.

EN9007:

Interviewer: Okay. Have you had double vision or any loss of vision?

Interviewer: Blurriness?

EN9007:

Interviewer: Have you noticed any changes?

Interviewer: Yes. And do you currently work?

Yes I do, but I don't wear them every day.

No, I'm disabled.

EN9007:

EN9007:

Interviewer: Okay. Do you currently wear glasses?

Competing Concerns

when wearing glasses?

Interviewer: Oh, okay. And are you able to get around okay? Or do you have difficulty seeing, even

Interviewer: Okay, why yes and no?

I would say yes and no.

EN9007:

appointments done?

Interviewer: Has sometimes being in the hospital or having surgery prevented you from getting eye

Yes.

EN9007:

past. You mentioned that you're on disability and that you have had some major surgeries in the past.

Interviewer: Okay. Okay, then. I wanted to know, again, patients have also talked about this in the

No.

EN9007:

Cues to Action

Competing Concerns

In-clinic Experiences

Vision Status

Knowledge-creating Experiences

Emotional Context

Resource Availability

Coding Density

EN9007: Because when I did, they tried to make sure that I'm up to date with all my appointments.

Interviewer: Okay. Okay, than. And in the past, has having a job or having a job to go to, has that prevented you from making your appointments?

EN9007: No, not at all.

Interviewer: Okay. And patients have also mentioned substance use, so whether that's tobacco or alcohol or drugs, has any--

EN9007: Say that again?

Interviewer: I was saying that sometimes patients have mentioned their own struggles with substance use. So this could range from cigarettes--

EN9007: I hope to God [00:22:33] to me. The last time I used was in 2008.

Interviewer: Okay, okay. And so would you say that that has affected your ability to go to doctor's appointments?

EN9007: I got to think about that one. I don't think so. Interviewer: Okay. And what substances have you used in the past? EN9007: Marijuana and crack cocaine.

Interviewer: Okay. And you said the last time you had used was 2008? EN9007: Say that again?

Interviewer: When was the last time you used?

EN9007: [00:23:17]

Cues to Action

Competing Concerns

In-clinic Experiences

Vision Status

Knowledge-creating Experiences

Resource Availability

Coding Density

Interviewer: Oh, sorry, I couldn't hear that last part. EN9007: Hello?

Interviewer: Yeah, hello, can you hear me?

EN9007: Barely. I don't know what's going on with this phone.

Interviewer: Yeah, sorry about that. We can move on to at least the last part, because again, you answered a lot of my questions. I just wanted to know when was the last time you had used, or if that had affected doctor's appointments or getting to them.

EN9007: Say that one more time?

Interviewer: Sorry, I just wanted to know when was the last time you had used. You mentioned using marijuana or crack cocaine.

EN9007: 2008.

Interviewer: 2008, okay. Again, thank you so much, [redacted], for your honesty and being able to share your experiences.

EN9007: No problem.

Interviewer: And now we're onto actually our last theme. So again, you've been doing a great job. This goes back to emotions. So you mentioned about how hearing about diabetes was scary. Do you remember going through any other emotions about diabetes or how it can affect you?

EN9007: No.

Interviewer: Okay.

Interviewer: Yes. And--

EN9007: When I found out it, I just tried to do what I can to live as long as I can.

Emotional Context

Interviewer: Oh, that's great.

And actually, I have reversed it where I didn't have to take the needles no more.

EN9007:

EN9007: So it came back through COVID, because I was in the house, eating up everything. Interviewer: Yep, I see.

EN9007: So now I'm paying the price.

Interviewer: Yeah. Sadly, that happened to a lot of people. We were all on our health goals and then we were told to stay in the house.

Cues to Action

Competing Concerns

In-clinic Experiences

Vision Status

Knowledge-creating Experiences

Emotional Context

Resource Availability

Coding Density

EN9007: Right.

Interviewer: Yeah. So I guess now that the world's opening back up, do you have a motivation to get back on track?

EN9007: Huh?

Interviewer: I said now that the world is opening back up, do you have a motivation to get back on track?

EN9007: Right, right.

Interviewer: Okay. Okay, then. Is there anything else that you want to share about your experiences, diabetes, your vision?

EN9007: Not at this time.

Interviewer: Okay. Well, again, thank you so much, [redacted].

***End of formal interview***

--- START OF INTERVIEW---

Cues to Action

Competing Concerns

Knowledge-creating Experiences

In-clinic Experiences

Vision Status

Emotional Context

Coding Density

Interviewer: Okay, so at least ten years. And do you know how many years it’s been since your last eye exam?

EN9008: At least five.

Interviewer: At least five years, okay. Thank you, [redacted]. And so like I mentioned, the study, we have some themes that we want to talk about. And we want to ask about experiences getting eye exams as a person with diabetes. Again, your honest thoughts are invaluable. You are the expert of your own experiences. I may ask you to say more or elaborate. That doesn’t mean that your answer isn’t good; I just want to hear more. And all your responses will be kept anonymous, so I just wanted to say that before you start.

EN9008: Okay.

Interviewer: Okay. So the first thing we have is resource availability. And this can include insurance, money, transportation, not having a home, or even being incarcerated. And a quote to kind of describe this is: “I was in a tough place, you know. I was on the streets. After squinting for like two years to see everything, I went back to see my eye doctor finally.” So I wanted to know if the theme of resources is familiar to you in terms of making it hard to get eye exams.

a sudden I stopped when my…it was kind of hard for me when my husband was sick and couldn’t get

I think mine were I didn’t really [00:05:22]. Oh, gosh. I just was going, then all of

EN9008:

Interviewer: Right. So just to talk a little more about it, so you mentioned that your family members, particularly your husband, was sick at one point in time, and so in that case, do you feel that it was hard to go to any type of doctor’s appointment?

insurance because I didn’t have full Medicaid. I had the one with the red and blue card, but I didn’t

Yes. And another thing, I didn’t have the insurance at that time too. I didn’t have the

important for me to get my eye exam, especially with diabetes and stuff.

something, I didn’t have a way. So all of a sudden, I just fell back in going. Now I know that it’s really

around. So when I did go…I went when I could, but if I had to take him or let him go to the doctor or

Resource Availability

EN9008:

Interviewer: I see. No, that makes sense. And anything else that kind of makes you think about how difficult it was to get eye exams in terms of resources?

dentist, like over $1,000.

eyes, but even with dentists, I always ended up paying a lot of money out of my pocket to go to the

have the statement insurance. It was kind of hard like even…I know we we’re just talking about the

EN9008:

Yes, like I didn’t have the money at that time. I had to pay for everything out of pocket.

Interviewer: Yeah. No, that makes sense. Thank you for sharing about that experience. That gives me a good picture about resources. And I’ll come back to it in case something else pops up in your mind. But for our second theme, the idea is cues to action. So this could be something that kind of jump-starts you to make an appointment or go to an eye exam. And some examples include appointment reminders, an annual eye exam schedule, maybe prompting by your primary care provider like we’ve talked about, or some type of ah-ha moment. So a quote that’s an example is: “I went for an eye exam because the doctor that I had, she examined my eyes. She said I have to send you to the eye doctor. That’s when I

started getting these eye exams.” So can you think of any cues to action or any kind of jump-starts that make you want to get or have made you want to get an eye exam.

five years. And then I noticed I can see far off, but when I look down, it’s hard. The words like go

Well, when I start seeing like my…I haven’t really had an eye exam in about at least

Cues to Action

Competing Concerns

Knowledge-creating Experiences

In-clinic Experiences

Resource Availability

Emotional Context

Coding Density

EN9008:

Interviewer: Yeah, and have you had any new symptoms lately or any changes in vision that have also made you want to get an eye exam?

it’s okay, but when I look, I could see a shadow coming around my eye.

Well, I don’t know if you remember, I talked to you I think. When I’m looking straight,

years, so [00:09:01].

together. And I need to change my eye glasses, because the ones I have, I’ve had those about eight

EN9008:

Interviewer: Yeah. Yeah.

EN9008: Yeah. It’s not hurting or anything, but it’s just an annoyance.

Vision Status

Interviewer: Right. And you mentioned that this happened recently, right? Because I do remember you telling me.

be…oh gosh…someone would have to refer me to them, you know, the eye doctors in Yale.

It happened about a couple weeks ago. So when I got it, I called for Yale, but I have to

EN9008:

Interviewer: Right.

EN9008:

Interviewer: Yes, absolutely. Thank you for sharing that, because I wanted to kind of bring that back up. So I’m glad that we’re getting that process underway. The referral is in, so now we’re just trying to schedule you an appointment.

EN9008: Yes.

And I just didn’t want to go to any eye doctor. I prefer to go to something at Yale or

Scott or whoever. You know, somebody that I’m familiar with.

Interviewer: And I wanted to ask you too, because sometimes I know you have just regular doctor’s appointments, so how do you remember those appointments?

EN9008: Well, I usually go when I have them. If I can’t make it, I’ll use the, you know,

[00:10:42] or something like that. Or reschedule.

Interviewer: Yeah. Do you write anything down or have a reminder system?

I mostly remember it. Or if I can’t remember, I’ll call and ask when is it so I won’t have

you waiting and I’m not coming in. You know how that is.

EN9008:

Interviewer: Right. Yeah. Okay. So it’s good that you kind of use those resources to at least remind yourself. Perfect. Okay. So again, if anything else pops up, you can always go back to themes. But I wanted to move on to the third one which is knowledge creating experiences. So these can be

experiences in your life that helped you to learn more about diabetes or even eye diseases. And it can include family or friends with diabetes, education by a doctor, or other resources including YouTube or books. And a quote from this is: “I mean, nobody wants to be blind. I watch what my sister went through. When I think of her living in darkness for so long, it’s like I don’t want to do that.” So I wanted to know in your life and experiences, how have you learned more about diabetes?

thing I’m behind in, and I don’t really like do my blood test. I don’t do that, so I know that’s important.

Well, I know that it’s very important to get a checkup and take your medicine. And one

Cues to Action

Competing Concerns

In-clinic Experiences

Vision Status

Resource Availability

Emotional Context

Coding Density

EN9008:

Interviewer: Okay. And you said that you didn’t want to accept that you had it. Say more.

100 pounds, but then I gained it back and it came back again. So I guess if I go and lose, lose, lose,

Yes, because one time they diagnosed me with it. And I went on a diet and I lost like

know in my family it runs in I think on my father’s side. I do believe that’s where it came from.

low or if it’s too high. And I’m basically learning. I really didn’t want to accept that I had it, so. But I

But I do have a family member that takes their blood sugar and see how much it is or, you know, if it’s

Knowledge-creating Experiences

EN9008:

Interviewer: I see. I see. And is there anyone within your family that you tend to go to when you have questions about diabetes?

the whole family in my [00:13:31] has it.

lose, lose, lose weight, hopefully that it will go away again. So it really is my sisters, brother, basically

EN9008: I didn’t understand you.

Interviewer: Oh, yeah. I was going to say is there anyone in your family that you tend to go to when you have questions about diabetes?

Me and my sister and my niece and…you mean go and see where it comes from or

EN9008:

Interviewer: Oh, North Carolina. Okay. Yeah. Again, thank you, [redacted], for sharing that information.

don’t know. So it’s just me and my sisters here, and then I have a baby sister in North Carolina.

where? No, not really. All of my older peoples that could tell us something where it came from, they

EN9008: Okay, excuse me a minute [00:14:33]. Interviewer: No worries.

EN9008: Okay. [00:14:42] to call my sister. Interviewer: Oh, okay.

EN9008: So my nephew…my son…calling for my son to pick her up, and I’m not…excuse me just one moment.

Interviewer: No worries.

EN9008: If I can’t get you back in, you call me back, okay?

Interviewer: Okay, I will. EN9008: All right. Hello? Interviewer: Yes, hello?

Cues to Action

Competing Concerns

Knowledge-creating Experiences

Vision Status

Resource Availability

Emotional Context

Coding Density

EN9008: Are you still here? Interviewer: Yeah, I’m still here. EN9008: Okay, maybe she’ll call back. Interviewer: Okay.

EN9008: Okay, [00:15:20] have to go pick her up. She probably was calling me, but I don’t think she knows [00:15:24].

Interviewer: Oh, okay.

EN9008: Okay. You can go ahead.

Interviewer: All right, so I’ll try and go through the next ones a little bit more quickly, but we’re almost done. The next theme is called in-clinic experiences. So this could include conversations you’ve had with doctors, feelings of trust and respect, customer service, and just your overall experience. So as far back as you can think with your eye doctor experiences, how has the experience been when you were getting your exam?

old days when I used to go, everybody remembered me. Hi, [redacted]! Hi, [redacted]! So I really, you

it’s been so long apart, that maybe every now and then somebody will remember me. But back in the

Good. Every time I go…when I go there, everybody remembers me sometimes. But now

In-clinic Experiences

EN9008:

Interviewer: Oh, thank you. It’s a pleasure being your doctor too. And I guess overall, what has made those experiences bad? If you have had bad experiences, what has made them bad?

I never had a bad experience, not with a nurse or the doctor. Everyone’s been good.

Not really. I really have not. Like I said, everyone always treated me nice and respectful.

including you.

used to going to [redacted]. So everything is nice. Every time I get a doctor, he is she is real nice,

know, takes getting used to. Of course, I’m not used to calling it…well, we called it [redacted]. I’m just

EN9008:

Interviewer: Oh, that’s good to hear. And it sounds like too, you were saying how people would say your name, Oh, hi [redacted]. Hi, [redacted]. So…

EN9008: Yeah, yeah. Well, I used to be [redacted] and they had to change from [redacted] to [redacted].

Interviewer: Oh, I see.

EN9008: But that’s been a lot of years ago. I’m going way back to a long, woo, back in the ’80s.

Interviewer: But that’s still…

Cues to Action

Knowledge-creating Experiences

Coding Density

EN9008: But yeah, everything has been nice. I never had no really bad experience with neither one.

Interviewer: Oh, that’s good to hear. And it sounds like when you found like…you felt like people knew you or people knew your name.

was in something that he was talking about, and he was talking about me, how I went and lost weight

Yes, yes. And I had a doctor, he moved away. He went somewhere else. And someone

EN9008:

Interviewer: I see. No, that’s good to hear. So it seems to be that relationship with your doctor and then also with the whole office.

to know that he thought of me when he was speaking of me. Yes.

and then how he was so proud of me. But he see me now and I’ve gained some back. So that was nice

In-clinic Experiences

Emotional Context

EN9008: Right. Right.

Interviewer: Okay. That makes sense. So the next theme—we’re getting in the last three. So the next one is vision status. And you mentioned this before, but I wanted to know if you could speak more to what about your vision makes you want to see an eye doctor? It could be your—yeah, you can continue.

going anyway to see an eye doctor. I just didn’t want to go to any eye doctor. I wanted to go to ones

Well, especially now that I’m seeing a shadow, I don’t like that. But I was planning on

Vision Status

EN9008:

Interviewer: No worries. EN9008: Hello.

Excuse me.

that I know. Because you know, they have an eye doctor near about on every corner. Excuse me again.

Interviewer: Hi, [redacted], I think I lost you for a second. EN9008: Hi, yeah! Okay.

Interviewer: But I know you mentioned a call back, so I just wanted to finish the last two slides. EN9008: Okay.

Interviewer: Okay, perfect. So you mentioned about changes in your vision, and thank you for sharing that. The next thing we have is called competing concerns. And so this could be anything from your job to childcare or babysitting, health problems, or even substance use. And I’m curious to know as much as you want to share, have you ever had conflicts that have prevented you from getting eye exams?

That stopped me a lot, and I had to pay out of my own pocket.

No. Really, no. Only thing I can think of that I said one time I didn’t have insurance.

Competing Concerns

Resource Availability

EN9008:

Interviewer: Right.

pocket. That was before we had Medicaid or Medicare.

And one time me and my husband both was going, and we had to pay out of our own

Cues to Action

Knowledge-creating Experiences

In-clinic Experiences

Vision Status

Resource Availability

Emotional Context

Coding Density

EN9008:

Interviewer: Right. Right. EN9008: So that stopped me.

Interviewer: Yeah. And I’m curious to know, because I know sometimes you look after your grandchildren as well, do you find that sometimes that affects when you can schedule your doctor’s appointments?

morning, I do the evenings. When I was working, I started doing evenings about 20-some years ago. So

Well, I try to schedule around them, so when they’re in school—well, I don’t do the

Competing Concerns

EN9008:

Interviewer: I see. Okay, yeah. And were there ever times where you were in the hospital and you weren’t able to make your appointments?

until 9 in the morning. So it’s good.

mother was working at night, so they didn’t get here for about 9-something because she go in at 11

I just let it stay that way because I enjoy the evenings. But no, I schedule around them, and when the

EN9008: When I was in the hospital?

Interviewer: Yeah, were there ever times that, say, you couldn’t make your appointment because you were in the hospital?

EN9008: Oh, well you know what? God has been good. Only been in the hospital that one time when I had COVID.

Interviewer: Oh yeah, that’s good.

EN9008: From all the years, that’s the only time that I’ve been and stayed over.

Interviewer: Okay, well that’s good to hear, and let’s keep it that way. I’ll knock on wood for you. EN9008: Yes, thank you Jesus.

Interviewer: Yes, yes. Okay. So that’s good to hear with this one. So now we’re moving on to the last theme before I open up the floor for you to mention anything else. So the last thing we have is emotional context. And it can include wanting to know more information, fear of learning the truth or the severity, denial of the current situation, or a wake-up call. And a quote to describe this is: “I didn’t think I needed it. And a lot of times when I go to the doctor, all of a sudden I need stuff. And I didn’t want that to happen, and I wanted to think that my eyes were going to be okay.” Does this theme feel familiar to you?

EN9008: Yes, ma’am.

Interviewer: How does it feel familiar?

Cues to Action

Competing Concerns

Knowledge-creating Experiences

In-clinic Experiences

Coding Density

EN9008: Pretty much sometimes…wait a minute. Say that again, because my [00:24:45].

Interviewer: No worries. So I know that when we were talking a little bit earlier, you mentioned how you didn’t want to accept you had diabetes.

EN9008: Yes, yes.

Interviewer: And that’s an example of, say, an emotion you may have had. And I’m curious to know with this shadow in your eye, I know you’ve described it as annoying, but are there other emotions that you feel when thinking about diabetes and how your eyes are right now?

and stuff like run together and I really can’t see it. And sometimes when I’ll be reading, it’s really

say I’m sitting on my couch and I can look at my TV pretty good. But when I look down, the words

Well, because like I said, like I can see far away—not far, far, by you know. Okay, like

Vision Status

Emotional Context

EN9008:

Interviewer: Yeah. So you’re saying this feels like a wake-up call to you.

And I’m concerned, like yeah, that’s the wake-up for me too.

leading me around, you know. You want to go one place and you came over here, but I want to see too.

me a little bit because, [00:25:53]. I don’t want to go blind. I want to see. I don’t want nobody

annoying me, and just like the shadow that I have, it annoys me. So I’m really…well, it kind of scares

EN9008: Yeah, for me to go on and get my eyes and stop putting it off. Like I said, putting it off because I was going to the eye doctor regularly. And then, like I said, at the time, me and my husband, we wasn’t getting no kind of help from the…I guess we was too young for that little blue card. I forget what you call it.

Interviewer: Yeah, Medicare?

EN9008: Yeah. And then we couldn’t get Medicaid or Medicare. Interviewer: I see.

he had to pay…like his medicine sometimes would be over $300.

So we had to pay for our own, and he was back and forth to the doctor a lot. Sometimes

EN9008:

Interviewer: Wow.

Resource Availability

EN9008: And mine was right much too. So that cut out a lot. And he had to go get surgery and all on his eyes. And sometimes when that bill comes out, you know…thank God that they can’t refuse you. They had to pay when you go to [redacted], you know.

Interviewer: Yeah.

doing good.

thank God we made it. I got my Blue Cross Blue Shield right now until they switch me over. So I’m

They got their fund that they’re not supposed to refuse nobody if they need help. But

EN9008:

Interviewer: Okay. Well, that’s good to hear, [redacted], because we definitely want to... EN9008: Thank you.

Cues to Action

Competing Concerns

Knowledge-creating Experiences

In-clinic Experiences

Vision Status

Resource Availability

Emotional Context

Coding Density

Interviewer: Yeah, and I’m glad that we’ve been able to kind of get the process started again for you to see an eye doctor. So that’s going to be on the top of our list for you in addition to doing the diabetes medications.

EN9008: Okay.

Interviewer: So before I end, I just wanted to open the floor to see if there’s anything else you wanted to talk about.

EN9008: No, that’s it, really. Just really two things that are on my way—or actually, three—but the diabetes and the eye exam and my dentist. I went to one dentist, but I didn’t feel like I didn’t like them, and I’m ready to go to someone that I’m familiar with. But it’s been a long time since I’ve been to [redacted], you know, [00:28:42] or Scott or wherever. But I really prefer to just go to [redacted] and let them look and see...[Rest of conversation redacted]

***End of formal interview***
