# Supplementary material for: Interrogating a framework for diabetic retinopathy screening adherence: Qualitative insights from a severely affected and under-adherent population
Source: PLOS Glob Public Health. 2026 Apr 2;6(4):e0006230. doi: 10.1371/journal.pgph.0006230 (PMC13046135; doi:10.1371/journal.pgph.0006230)
Supplement: S3 Text — (DOCX) [file pgph.0006230.s003.docx]

**S3 Appendix.** Node summaries with coded transcript excerpts by theme

**Theme: Vision Status**

Files\\EN9001_Transcript - § 2 references coded [ 9.22% Coverage]

Reference 1 - 3.33% Coverage

Ever since my teenage, my younger years I’ve always worn glasses. Can’t see far away. Can see close up but not far away. When I went to the hospital with diabetes, like I told you I stayed in there for two weeks, and they had me on saline machine. And I was on that for two weeks. And it got to the point that I couldn’t see with my glasses that without my glasses I have perfect vision.

I told the doctor about it in [redacted], et cetera and of course they paid no attention to it. Once they took me off that machine from giving me that, two weeks later my vision went back to poor again. So it’s something in that stuff that did something inside my system. But without it I went back to my port vision.

I’ve always wear glasses. So my vision is always been pretty bad.

Reference 2 - 5.89% Coverage

I think the eye twitching was all associated with diabetes because when I went there he was telling me that you know back there was swollen. He was telling me that I had blood in my eye and et cetera. So somehow that was all connected because I hadn’t hurt myself. I wasn’t punched in the eye or nothing else. So it was all associated with diabetes.

I think I was starting to just lose my vision and that might have been why you know it was jumping because I wasn’t getting enough oxygen or what happened. But now what the strangest thing, since I’ve been getting my eyes worked on. I watch TV and stuff with no glasses.

If you notice me and you are talking now I don’t know if you can see me I don’t have glasses. I have worn glasses my whole entire life 24/7. I would only take my glasses off to sleep. Now I can’t eat with glasses or anything. I’m in the house I’m watching TV I don’t need glasses. I even drive with no glasses. At night time I will put them on because it just makes me see a little clearer. But I haven’t gotten any new glasses in about 2 years because I’ve been waiting for the doctor to say you know my eyes are kind of settled. Because every time I go she looks at him and you know we need to do this and we need to do that so we just going out spending money on classes and my vision is and right yet that just to me it doesn’t make much sense but...

Files\\EN9002_Transcript - § 6 references coded [ 5.30% Coverage]

Reference 1 - 0.16% Coverage

I had little blood vessels in my eyes.

Reference 2 - 0.51% Coverage

They look like hairs hanging on the inside of my eye. That you can’t see from the outside but I can see from the inside.

Reference 3 - 0.46% Coverage

Wasn’t changed, and my vision is just like, I don’t know what they were just blood vessels from diabetes.

Reference 4 - 1.65% Coverage

I was like seeing I don’t know. It was like, seeing something in your eye. I thought something was hanging from my head. And nobody else could see it. But me so I figured it was on the inside. So I went to an eye doctor and they told me that I had some whatever it was blood vessels that popped or whatever. And that’s what I like to see Dr. [redacted] and they did a laser surgery on my eye.

Reference 5 - 0.80% Coverage

I don’t think my vision has changed. Not that I know of. I don’t haven’t seen much of a change in my glasses. Okay. You know, when I go to the eye doctors is pretty much the same, I believe.

Reference 6 - 1.72% Coverage

if it wasn’t for that, I probably wouldn’t have gone when I did. But on a regular I was go because you know, I had the insurance that they were paying for. So I went every year. Right? But if it wasn’t for that, I was as a matter of fact, I was at work and it just popped up all of a sudden I like I said, I thought it was a fly flying around my head and nobody else work could see it. And it started getting worse.

Files\\EN9003_Transcript - § 4 references coded [ 17.58% Coverage]

Reference 1 - 0.20% Coverage

my eye, on my vision. It was getting blurry vision.

Reference 2 - 0.64% Coverage

And then all of a sudden, I got to see some blurry sometimes, and it was back and forth. But I did not understand what it was about, and the increased pressure in the eye.

Reference 3 - 16.29% Coverage

Oh, when that situation, when it happened that day. It was a situation that I worked…I went to work with this company that I worked overnights. I mean, understand, my diabetes, it was related to my vision. It was hurting my vision. So, I worked the shift straight, nightshift, and the next day, I had to wake up early in the morning. So, I took a day in the middle.

So, on my way to go to work, like 5:00 o’clock in the morning, I wake up, I was driving it, and all of a sudden my eyes started getting really blurry, and then all of a sudden I felt the light that was coming at me, oncoming traffic. I was seeing it was a really huge bright light, and like realizing, I was like, this is not normal. This is something happening, and then all of a sudden, I noticed that I was not reaching far, that my distance of seeing, and I was like, okay, something is wrong on my eye because I don’t see any of the traffic…usually regularly seeing it far away.

And all of a sudden, I saw like…I call it water, but I think it was tears. I mean, that’s what I thought it was…when you put water, right, on a window, and then you see that water the first drops coming down, I just saw that in my both eyes, not on the left eye only. Actually, on both eyes. And then all of a sudden, I didn’t understand that, nothing about high pressure inside the eye, nothing, like I said before. I didn’t have education for that.

All of a sudden, I just started seeing more cloudiness, and then I just pulled over, and then I was like, in my head, I was thinking that I was tired from that shiftwork the couple days before. So, I just pulled over and then rest, you know, for about an hour. So, when I wake up for the noises around me and try to see, and then all I was seeing, it was a big cloud in front of me, not even seeing the hood of my car. So, all that, I mean, rushed back. I mean, I called my father-in-law. They went and picked me up, and then they drove me back to the hospital, emergency room, ER, right here in, where I live. What they did is they just checked on me, and then because I didn’t have any insurance at that moment, so they said they tell me they cannot do anything for me.

So, long story, short story, I just end up going to, I mean, a day after, [redacted] University. Somebody tells me, listen, if you go up there, then tell them that you have no insurance at all. They’re going to take care of you. You need to go there. So, I just went to the ER in [redacted], and then one of the doctors…I don’t know if it was a practitioner or the doctor…they put eyedrops on me and my vision went back. So, he told me, you need to see your doctor immediately because you have a problem with your eyes. If you don’t treat this, you’re going to have a lot of problems. And that day, my vision went back to normal. I just walked out there, driving my car myself. I mean, even with drops on my eye, I could drive myself back home, that day.

But I was like, okay, this incident happened. I mean, so, I took a day off after that to start to make the arrangement to go back and see the regular local doctor, but they were so booked up, there was nothing. They couldn’t handle seeing me so soon.

So, in the days after, that increased again. So, I just ran out. I went back to the ER again in [redacted]. That’s when they told me and sent me where they have a Department out there, where they treat me, giving me a treatment. And then one of the doctors, I heard him, he told one of the students, he said, listen, he’s got a clot at nine o’clock here and then he told me, “Well, you need to go to that department and tell them that you need to have lasers done before your eyes get worse.”

So, I just went there, and I told them…this is the department for students. So, they start working on it, but I don’t think they understand what I say, or they don’t want to listen. So, they are students and teachers…and I don’t know what happened. So, just one doctor…I don’t remember his name…I forget his name. I know it was Ian. So, when he reads the chart, he tells me, “You’re going to be blind,” and I was like…I started crying. I went in tears. And then at this moment, my right eye…starts seeing, but my left eye clouded up. And they told me, if you don’t do anything, your eye is going to pop out, going to blow out. I had a 69 pressure on that eye, on the left eye.

Reference 4 - 0.45% Coverage

I’m not afraid to be blind at this point. Honest to you. Like before, I was afraid to be blind. [voice cracking] Sorry.1

Files\\EN9004_Transcript - § 5 references coded [ 4.21% Coverage]

Reference 1 - 1.20% Coverage

That’s when I thought I had an eye problem. That’s why I know. My eye was - when I first found out - was 50 in my eye. It was pain in my eye and I had to go to the emergency room. And it just so happened I didn’t know him them; Dr. [redacted] was one of the doctors. He was also at that - turned to be my - whatchacallit - surgeon. Yes, and then, to be my doctor after.

Reference 2 - 1.33% Coverage

Well, it’s weird to say that I was not - I was out a way and my eye got a pain on a - I even know the day - on a Wednesday. And then, all the sudden, my eye kept blinking out and coming back, going out and coming back. And then, it totally went out and I started getting a pain in my eye, which was - my eye pressure was 50. So, it was like having a baby in the eye. So, that’s why I made sure I had my eye exam.

Reference 3 - 1.41% Coverage

And you know what? Whatever you - whoever you’re talking to, whatever you do; if somebody says that they’re seeing white, everything looks kind of white to them and foggish, that happened to my eye, did not even realize it, that it was going on. So, I started seeing white and could hardly see stuff. And so, that’s what made me afraid to go, which was crazy. So, it ended up the way it did. You start seeing white.

Reference 4 - 0.20% Coverage

ecause my eye problem, I had an eye problem, I couldn’t see.

Reference 5 - 0.08% Coverage

You know you can’t see

Files\\EN9005 - § 3 references coded [ 2.09% Coverage]

Reference 1 - 0.89% Coverage

I said it must have affected my eyes at one point in order to have my eyes checked _____

Reference 2 - 0.52% Coverage

Okay. Have you had any big changes to your vision?

Reference 3 - 0.68% Coverage

Just like we talked about, blurry vision or something like that.

Files\\EN9006 - § 2 references coded [ 0.69% Coverage]

Reference 1 - 0.17% Coverage

My vision changed.

Reference 2 - 0.52% Coverage

I noticed that things that I used to could see were blurry.

Files\\EN9007 - § 3 references coded [ 7.16% Coverage]

Reference 1 - 3.61% Coverage

Interviewer: Okay. What did you see the eye doctor for?

EN9007: I forget. I think some type of cataracts. They say, I believe-- I’m not even for sure what they were seeing my eyes for. I really don’t remember. But I know they are giving me dilation, when they drop these drops in your eyes to look at your pupil, if I’m saying it right?

Interviewer: Yeah, pupil, yep.

EN9007: Yep. But I know I’m in the computer. And I used to go down on [redacted]. Actually, that’s the last time I went to the eye doctor, on [redacted].

Interviewer: On [redacted], okay. So you were saying that’s the last time you went.

Reference 2 - 2.04% Coverage

Interviewer: Okay, that’s good to hear. I wanted to ask how your vision has been since being diagnosed with diabetes.

EN9007: Pretty good. It’s been pretty good, I guess.

Interviewer: Have you noticed any changes?

EN9007: _____ [00:20:16] just for medications that I’m taking. I’ve had this blurriness.

Interviewer: Blurriness?

Reference 3 - 1.52% Coverage

Interviewer: Okay. Have you had double vision or any loss of vision?

EN9007: I wouldn’t say double vision. What I’m saying, there’s like two of some.

Interviewer: Okay. Do you currently wear glasses?

EN9007: Yes I do, but I don’t wear them every day.

Files\\EN9008 - § 5 references coded [ 7.86% Coverage]

Reference 1 - 0.87% Coverage

Well, I don’t know if you remember, I talked to you I think. When I’m looking straight, it’s okay, but when I look, I could see a shadow coming around my eye.

Reference 2 - 0.95% Coverage

It happened about a couple weeks ago. So when I got it, I called for Yale, but I have to be…oh gosh…someone would have to refer me to them, you know, the eye doctors in Yale.

Reference 3 - 0.81% Coverage

And I just didn’t want to go to any eye doctor. I prefer to go to something at Yale or Scott or whoever. You know, somebody that I’m familiar with.

Reference 4 - 1.64% Coverage

Well, especially now that I’m seeing a shadow, I don’t like that. But I was planning on going anyway to see an eye doctor. I just didn’t want to go to any eye doctor. I wanted to go to ones that I know. Because you know, they have an eye doctor near about on every corner. Excuse me again. Excuse me.

Reference 5 - 3.59% Coverage

Well, because like I said, like I can see far away—not far, far, by you know. Okay, like say I’m sitting on my couch and I can look at my TV pretty good. But when I look down, the words and stuff like run together and I really can’t see it. And sometimes when I’ll be reading, it’s really annoying me, and just like the shadow that I have, it annoys me. So I’m really…well, it kind of scares me a little bit because, _____ [00:25:53]. I don’t want to go blind. I want to see. I don’t want nobody leading me around, you know. You want to go one place and you came over here, but I want to see too.2 And I’m concerned, like yeah, that’s the wake-up for me too.

**Annotations**

1 This was a very humbling moment in the interview for me.

2 Independence, not wanting to inconvenience.

**Competing Concerns**

Files\\EN9001_Transcript - § 1 reference coded [ 1.34% Coverage]

Reference 1 - 1.34% Coverage

No, like I said, I, you know, maybe because of the kids or something like that, you know, I’m a foster parent. And, you know, it has been a time that I’m on my way and one of my kids artistic and he act out, you know, and so I’m in the car, and he’s throwing faces and punches. So I need to go home, you know, so yeah,

Files\\EN9003_Transcript - § 2 references coded [ 2.21% Coverage]

Reference 1 - 1.32% Coverage

I think I haven’t missed much [laughter] since that happened. But I mean, I think if I missed an appointment it was because I had no one to drive me down there, but I don’t think I missed any appointments. I think it was either switching schedule for any other reason, through the building or something happened, but I don’t think we missed any.

Reference 2 - 0.89% Coverage

Yes, I guess this is one of the times, with this pandemic, that they had to cancel one of my appointments. I guess it was twice they canceled appointments because of the pandemic. Besides that, I think I went to all my appointments.

Files\\EN9004_Transcript - § 2 references coded [ 1.64% Coverage]

Reference 1 - 0.59% Coverage

Okay, yeah. Because there’s always a lot of competing things that can go on. Do you think you were busy - that was a busy time in your life or there were other…

EN9004: No, no.

Reference 2 - 1.05% Coverage

Did you find it hard to get time off?

EN9004: No, never, never. If there was snow on the ground, whatever, I’ll get my ride to pick me up or somebody work in the store, whatever. No, never. I always made my eye - I came to my toe appointment, I cancel whatever if I have to. But I will not cancel eye appointments.

Files\\EN9007 - § 1 reference coded [ 3.18% Coverage]

Reference 1 - 3.18% Coverage

EN9007: No, I’m disabled.

Interviewer: Oh, okay. And are you able to get around okay? Or do you have difficulty seeing, even when wearing glasses?

EN9007: No.

Interviewer: Okay. Okay, then. I wanted to know, again, patients have also talked about this in the past. You mentioned that you’re on disability and that you have had some major surgeries in the past.

EN9007: Yes.

Interviewer: Has sometimes being in the hospital or having surgery prevented you from getting eye appointments done?

EN9007: I would say yes and no.

Files\\EN9008 - § 3 references coded [ 3.87% Coverage]

Reference 1 - 0.80% Coverage

No. Really, no. Only thing I can think of that I said one time I didn’t have insurance. That stopped me a lot, and I had to pay out of my own pocket.

Reference 2 - 0.72% Coverage

And one time me and my husband both was going, and we had to pay out of our own pocket. That was before we had Medicaid or Medicare.

Reference 3 - 2.35% Coverage

Well, I try to schedule around them, so when they’re in school—well, I don’t do the morning, I do the evenings. When I was working, I started doing evenings about 20-some years ago. So I just let it stay that way because I enjoy the evenings. But no, I schedule around them, and when the mother was working at night, so they didn’t get here for about 9-something because she go in at 11 until 9 in the morning. So it’s good.

**Emotional Context**

Files\\EN9001_Transcript - § 7 references coded [ 12.03% Coverage]

Reference 1 - 0.85% Coverage

Umm ignoranance, that that would be the best words that I could capture out of all that, because I think a lot of us, especially black folks, but you know, we tend to not accept being a diabetic.

Reference 2 - 0.62% Coverage

So, again, that’s where the ignorance comes in. And that was part of the first paragraph that I said to you, we hear it. But we don’t accept it.

Reference 3 - 0.91% Coverage

She had one of the best purchase attitudes I have ever, ever, ever run across, you know, she talked to me, like I was a little boy. And seriously, and she just she, you know, she, I got relaxed, like calm down.

Reference 4 - 0.12% Coverage

you felt the someone cared.

Reference 5 - 4.15% Coverage

But I don’t know if anything could have made it quote unquote, better. I’d say I can’t get I can’t get any better than what the doctor did. By by sitting down. by sitting down and giving me respect. And really showing that she cared was the best that it could be. You know, because by her care, and showing she cared, made me honestly start caring about me. You know that there is a chance you know, you know, we can get better? Because there’s some days she’ll tell you I’ve been there. And she look at the computer. Well, you know, we need to do laser surgery and the needle in your eye. That’s a different thing they call the name, you know, I get to looking at it, you know, cuz I hate needles. Oh, don’t worry about it. You know, it ain’t gonna hurt, you know, just a pinch, you know, you know, and so she, you know, she made it just so comfortable and relaxing. You know, and you felt she’s caring about you. You’re damn fool not to care about yourself.

Reference 6 - 2.29% Coverage

Yeah. Because when you go places and you find people with not nice personality, or attitude, you know, it kind of tends it. You don’t even want to go back. Right? You know what I mean? Your wife or husband say you got an appointment tomorrow, but I don’t even feel like I’m not going you know, yeah. And you’ll go through your troubles rather than go because, you know, they don’t even care. You know, so that’s why I don’t think that you know, anything could have they could have done anything better than than what she did.

Reference 7 - 3.09% Coverage

Fear is fear. The biggest fear is actually fear.

When you’re talking about people. now I can only a lot of times speak for black folks because we talk together. I’m not prejudice or nothing but I’m not in the circles you know of Spanish people or white folks. Around black folks we don’t like anybody messing in our eyes.

That’s just the fear itself. That’s the main thing. Even today when you know I talked to some of my friends and set of church friends and I tell them about I got a needle my eye and I am going next week to get one they cringe. Because if they’re gonna look at your feet or something then it’s we cool we OK. But we’re talking about the eyes you know that’s that’s a real drawback for a lot of us.

Files\\EN9002_Transcript - § 9 references coded [ 9.35% Coverage]

Reference 1 - 1.26% Coverage

No, I had my experience, I let it go. So if it happened, it happens. You know, whenever it comes back, like I said, Now, sometimes it comes back and it disappears before I can even get to my appointment. So the last time I went was seeing Dr. [redacted]. And it had already cleared up on its own.

Reference 2 - 0.96% Coverage

I didn’t see any bad thing about it. We hit it off. And the first time I walked through the door, we had, you know, me, her, her assistants and the nursing staff. Very good. nothing bad to say about any of them. They’re good.

Reference 3 - 0.63% Coverage

I’m aware, and I go about whatever I have to do, but you know, I keep an eye on it. I wear my glasses every day. Except for when I’m close to the phone.

Reference 4 - 0.59% Coverage

Don’t let anybody play with my eyes. trust her. Yes. to the fullest. You know, so when they told me she was out there, that’s where I went.

Reference 5 - 0.17% Coverage

I won’t let anybody else touch my eyes.

Reference 6 - 1.97% Coverage

I think she’s an all around good person. She’s good spirit is very happy. He enjoys what she does. And she takes the time out to enjoy her patients like me. Wonderful. So you found that she was making the time she made the time for patients like you. And yeah, I can hear her when I go and visit how she reacts with other patients and how they react with her. Because you know, she is kind of loud. So her voice does carry. She’s She’s just a complete sweetheart.

Reference 7 - 1.39% Coverage

Well, it really didn’t bother me. I was it takes a lot to really get me riled up about anything. I took care of it. It was rectified. And I know what to do. Now, if it ever happens again. I’m really not worried about it. Don’t worry about it. I know I’m in good hands with Dr. [redacted]. And I’ve got God on my side, so I’m good.

Reference 8 - 1.58% Coverage

A little bit of family. I’m very private person. I don’t tell everybody my business. So my friends don’t know. Except for you know, maybe if I post something on Facebook, about my closest friends and only ones my family that knows people who are on my list of emergency, which would be my sisters and my mother. Other than that, I family don’t know what I go through.

Reference 9 - 0.80% Coverage

You mentioned how you consider yourself to be a private person. Have you and your family members ever shared their experiences? Have you ever shared your experiences with your eyes?

Files\\EN9003_Transcript - § 10 references coded [ 29.63% Coverage]

Reference 1 - 0.14% Coverage

I did not think it was so important,

Reference 2 - 1.97% Coverage

Because of the way the doctor…I mean, I don’t blame at all, but I think there was a short explanation from the doctor to the patient and let me understand the situation that I was going through, because all I’ve seen, they’d given me a treatment, and they tell me, we’re doing this because we don’t want you to lose your vision. Okay. I understand that, but for me at that time, having 20/20 vision, I couldn’t understand the hardness, I would say, that I was running. …oh, hold on, let me just answer this real quick.

Reference 3 - 1.57% Coverage

Like I said, I mean, I didn’t feel, you know, they needed to have that treatment so bad. I thought it was something like, okay…it isn’t getting lost. So, I mean, it was like I was in that stage, okay, I’m going to need glasses pretty soon. It was like kind of, like, okay, I mean, I’ll go with the flow. I was not rushed or worried to go back to the treatment, because like I said, I did not understand how bad my eye was.

Reference 4 - 0.27% Coverage

I started crying. I went in tears. And then at this moment, my right eye

Reference 5 - 7.21% Coverage

Thanks to the Internet, YouTube, might as well, because being…like I told you before, I mean, from being a truckdriver and being on the street, and after this incident happened to me, kind of blind by myself…I mean, kind of blind. So, I had to find out something to help me to entertain myself and keep my mind busy and try to focus on something because it was a shocking moment. I’m sorry. [voice cracking] I’m sorry. It was a shocking moment, you know, so, crash, my life. I’ll say, this was a crash on my life.

So, being someone that was always willing to help and work…I had to focus on something that was…and I’d like to understand the study and be aware of where diabetes disease came from, and then all the eye diseases are from, and how exercise and how to reverse all this, how that impacted me.

So, YouTube, they have a lot of crap, [laughter] but they have a lot of good, really good information about it, like colleges, universities. They’re speaking and they have real facts and education. So, from some of those videos, I’ve been taking what I think is helpful for me. Because like I said, some other ones, I don’t even understand what they’re talking about, or I don’t care about it, and some of them are really fake, and they are more business and trying to sell products, stuff like that. So, they have some other ones, like [redacted] University. They really work really good on that, on stem cells and stuff like that and education about macular degeneration disease, diabetic retinopathy. And then they have another university in [redacted]. I forget the name of it right now. And they have another hospital in [redacted], where they work on all this, and they help the people on diabetic control and diet control to improve the eye health and then the diabetes itself.

That’s where I get more of the information. I really try to understand and educate myself.

Reference 6 - 1.93% Coverage

I’ve got four sisters. They all have diabetes. Before I used to be the one with the worst diabetes, and right now, just for you to know, I mean, my diabetes was 14 up to 16, my A1C, before this happened. It was really way out of control. They never put insulin on me. But after that, learning how to control the pancreas, the fluid, how to control myself in food and some other interests. So, right now, I’m the expert. My sisters, I’m helping them when their diabetes is out of control. My A1C right now is 6.3.

Reference 7 - 1.71% Coverage

Yeah, that’s what I’m becoming now. Now, when they’ve got a problem with their sugar and they are too high…I mean, when you understand how your body works, it’s so much easier to understand it. I mean, when you’re fasting, after night, and how the vegetables and the natural foods and how you prepare stuff like that, you almost don’t need medication no more because your body starts working by itself, little by little, and it’s getting back.

Reference 8 - 9.03% Coverage

Before, I felt like I was a number. I was another number, not even a patient. I mean, for some of the clinics where I used to go before, because I was asking questions and then not really many right answers. So, right now, Dr. [redacted], I’ve got to tell you, I love her. I’m not going to change. She has the best attitude.

Some of the doctors out there, I mean, I have to tell you the truth, some of those other doctors that’ve been helping her, they’re really good, but they have one or two of them, they need to have better concern in their careers, and change their careers because they don’t know how to handle patients, you know.

Dr. [redacted], she takes the time to talk to me and make me understand the problems that I’m going through. Sometimes I’m asking questions and she tells me, “You know the answer, EN9003.” [laughter] You already know. Yeah, like, okay, because I want to hear it from the expert. She’s the expert, right. [laughter] She will tell me and then repeat it again and then she goes back, and she shows me pictures, like right now for the injection. When I was kind of confused to have the injection, I mean, I’m being honest to you, to have that injection in the eye every month, and she makes me understand it. I mean, we’ve got to keep it under control.

Because, I mean, I was kind of disappointed to have the same treatment monthly…and I just told Dr. [redacted], “Listen, I don’t want this anymore because for two years and being through this, you think I can put an injection on me and have it making no changes.” So, even with the diet I’m using now, I have more control. I understand it there. If I have control, my diabetes, and all the stuff I’ve been doing, plus the injections, yes, it’s coming down, little by little. It’s not happening big in the eye, but it’s coming. I mean, I see improvement, little by little, but it’s coming.1

I had a good experience with the office out there at [redacted] and some other ones, but before that, I mean, some of the doctors…I don’t know if they don’t want to waste their time because they need to quota with the numbers of patients, and then they just want to wipe off the office, and then listen, I need to do what I need to do, and you just come back next month. I mean, I didn’t understand that. I did not understand that.

But right now, it seems like it is a good experience, the way I am right now.

Reference 9 - 3.80% Coverage

No, not at all, because I’m not employed. I’m kind of self-employed right now doing whatever stuff I can handle here or there because of my condition. Like I said, I used to be a truckdriver, so I’m not able to drive the big trucks anymore. I’m doing right now, some kind of house wax and truck wax. I’ll do all my vision allows me. That’s the kind of job where they need to be really careful, what I’m doing, the chemical supply and the wash and stuff, and the details, skip here, skip there. So, I have to go back.

So, if I don’t feel that vision is not allowing me that day, most likely, what I’m doing is friends and people that I know. So, they understand what I’m going through, and I can call them up and cancel. So, it’s not like I’m running a business with people that don’t know me. I don’t have to explain to every single one of them. Some of them can allow me to do the work. So, that’s why I try to keep moving and keep my head, and make me feel like I’m not useless. You know, I still can do something.

Reference 10 - 1.99% Coverage

Well, that’s a good question. [laughter] Right now, I’m doing the treatment, and honestly, I was hoping that I can get my vision back. Honest to you. I’m working together with you guys and then I want to have my vision back. The way the system is, I don’t understand the FDA, the regulations they have and stuff like that. I don’t feel right now at this moment my vision is stable, that it is controlled. I’m not afraid to be blind at this point. Honest to you. Like before, I was afraid to be blind. [voice cracking] Sorry.2

Files\\EN9004_Transcript - § 13 references coded [ 16.14% Coverage]

Reference 1 - 0.39% Coverage

No, I was just scared to go, I don’t know. I don’t know why. I guess I didn’t want to know what was going on with my eye.

Reference 2 - 0.21% Coverage

That’s what I’m saying. I was afraid of what I might find out.

Reference 3 - 2.17% Coverage

Okay, I have several answers to that question. I didn’t seek out any more information. I have three of my family; my father’s mother, sister, my father’s sister, and my father’s first cousin. All three of them had lost their sight. And now, my father’s first cousin, also, had lost his sight. They’re all from diabetes. So, I didn’t seek out any information because that’s what’s scaring me. It’s crazy to say but what happened to them, I was afraid that if they examined my eyes, they would tell me that. But it ended up that way anyway.

So, you know, I thank God I didn’t go that way. What I’m saying is I was like that until my surgery.

Reference 4 - 0.51% Coverage

And they didn’t tell me but I’m assuming that it was diabetes.

Interviewer: I see. So, if anything, that added to the fear that you were mentioning.

Reference 5 - 4.87% Coverage

Okay. That’s, you know, at first, and then, second. When I went there - well, I don’t want special attention. I just want to be treated like I’m a person. Okay, at first, the nurses I had with Dr. [redacted] were always great. Always, always great. But it seems like the nurses I get with Dr. [redacted], because I don’t know them, sometimes they can be a little rude-ish. Not rude. Like the other day, I was there and I was waiting and I have _____ [00:18:01]. So, I only had a limited amount of time and the time was going, going, going. And they brought me in and said, “You’ve got to be waiting in another room to be seen.” I said, “Fine.” So, I’m sitting out there waiting, waiting, waiting. Everybody’s getting called in except me. So, I said, “What’s wrong? Why am I not called?” “Well, there’s a lot of people ahead of you but I see where you are.” And I said, “I’m sure Dr. [redacted] wants to see me.” You know, he was going to do an injection in my eye that day but he didn’t. So, I said, “But I can’t be seen, I’ve got to come another day and I don’t really want to do that.”

So, another nurse came - the one was gone, that one, and another one came back and she brought me in the room. And I said, “Sweetie, why do you bring me in? Because they told me that they’re going to bring me in when Dr. [redacted] was done.” And she said, “Oh, if you don’t want to be in the room, somebody else can come in the room.” And I thought that rather rude.

Reference 6 - 1.62% Coverage

Oh, no. Oh, no. Because you know what? I try to be civil with my mouth because sometimes I can be a little curt; sometimes be a little out of the way. So, that’s why I was being really calm talking to her in the office, in the room.

So, no, I’m going to never not go but I’m going to have to go pretty - if they do it again, I’m going to have to go somebody’s - or whoever and explain to them that they’re doing that to me. No, it’s not going to never stop me from going to the eye doctor, no.

Reference 7 - 1.63% Coverage

My eyes, my eyes. That’s my eyes, that’s not their eye. They don’t care about what happens to my eye. I do. So, why would I not go for an eye docto3r’s appointment where I love the doctors? I love Dr. [redacted] and Dr. [redacted], I really do. And I told whoever my insurance people were, “I want them. I do not want to change where I go for my eye doctor. I don’t want to do nothing. If I can’t go to them, I’ll pay for it myself,” you know? I just like Dr. [redacted] and Dr. [redacted].

Reference 8 - 0.20% Coverage

And do you trust them as your doctors?

EN9004: Very much.

Reference 9 - 0.29% Coverage

But at this point, you want to see them. Like you don’t want to change.

EN9004: Never.

Reference 10 - 1.23% Coverage

I’m going to say this - excuse me - I’ll say this. One day, Dr. [redacted] was gone on a Friday and someone came and took her place. I’m like, “Who is this? I don’t want to see them. Why am I here if she’s not here?” [laughter] I think - I don’t know what I did. I was really snotty; I know I was. But because if it’s not Dr. [redacted], don’t have that appointment for me.

Reference 11 - 0.62% Coverage

I did know ahead of time, she did tell me. But I said, “I’ll come on Thursday, then,” because I didn’t want nobody else. I didn’t want nobody else. No, no, I’m not going to see them, no.

Reference 12 - 1.25% Coverage

Yeah. I’m just saying she’s so good. She knows me, I’m saying from 2015 to 2018, she knows me and, you know, she knows me; how I talk, how I act, you know. People at work didn’t even notice it. I’m at work and I was talking. Ain’t one of them say nothing. And I took the bus to the clinic downtown. It was weird, too. It was so weird. How can I have a stroke and not even know it?

Reference 13 - 1.14% Coverage

I hated it when I had to stop driving. You drive here, you drive there. Well, now you’ve got to depend on a bus or whatever to get where you want to go. I usually go to [redacted] - [redacted], [redacted], you know, drive in and wherever. But now, you can’t get on the bus and say, “I want to go to [redacted]. I want to go to [redacted] now.”

Files\\EN9005 - § 1 reference coded [ 0.48% Coverage]

Reference 1 - 0.48% Coverage

Appointments, really. Mainly, it’s been me.

Files\\EN9006 - § 11 references coded [ 9.40% Coverage]

Reference 1 - 0.54% Coverage

So, I keep them as current issues. They were priorities, so.

Reference 2 - 0.79% Coverage

You need to because it’s bad enough I lose my mobility. I’m a diabetic. You need to visit.

Reference 3 - 0.10% Coverage

Insecure.

Reference 4 - 1.40% Coverage

Just don’t have a good feeling about it, you know. Things that you’re familiar with or things you’re not familiar with, you know, lean on [redacted] things.

Reference 5 - 1.01% Coverage

--or not? Okay. So, you wanted to know more about diabetes—how it could affect your organs?

EN9006: Absolutely.

Reference 6 - 1.73% Coverage

Especially if you feel like you can’t eat the things that you think you can, you know. You’re already enjoying it even prior to being diagnosed. So, you have to consider something more popular--

Reference 7 - 0.65% Coverage

Reading is fundamental, no. It’s not hard to find information about it.

Reference 8 - 0.34% Coverage

It’s a part of your senses. You need it.

Reference 9 - 1.56% Coverage

Oh, well, what’s the A1C now compared to when it was recommended by your primary care, you know. Your vision changes as you exercise, you know. You just have to stay on top of it.

Reference 10 - 0.04% Coverage

Yes.

Reference 11 - 1.25% Coverage

They’re very informative. They tell you about whatever you needed prior to and they walk you through it. So, there isn’t any mystique to it.

Files\\EN9007 - § 7 references coded [ 15.48% Coverage]

Reference 1 - 5.12% Coverage

Interviewer: Okay. So it seems like transportation definitely resonates with you. I wanted to know-- again, and other examples have been not having secure housing, so being homeless or being incarcerated.

EN9007: Yes, yes, yes, yes. I’m homeless as we speak but I have somewhere to be at for now. But I am definitely homeless.

Interviewer: I’m sorry to hear that. How have you balanced not having housing with going to appointments?

EN9007: It’s difficult, but I manage.

Interviewer: Have there been any tools that have helped you, whether that’s shelters or family members?

EN9007: I refuse. I refuse to go in anybody’s shelter because I had a bad experience there once before. All of my belongings was taken away, stolen. And I addressed the problem. If I got too upset they were saying I’m aggressive and all of that. I don’t got time for that.

Reference 2 - 2.06% Coverage

EN9007: Well, just I wasn’t able to get there, that’s all. If I would have had somebody to come bring me, I would have been there. And then I have to go through stress of asking somebody to bring you, have to deal with they attitude and whatever they was supposed to be doing at the time. I don’t want to go through that. That’s why I didn’t make it.

Reference 3 - 0.81% Coverage

Interviewer: Yes. And what did you think of that information at the time?

EN9007: It was scary. Very scary. I hope I’m not one of those.

Reference 4 - 3.71% Coverage

EN9007: Well, I go back-- because my granddad had diabetes. And what ended up happening to him, he had to do dialysis, at the end of the day. I’m trying not to do that. How could I explain this? Yeah, I’m just trying not to do what he did, which is go on dialysis and he soon gave up and gave in. Now he’s in Heaven. I don’t want to go through that.

Interviewer: Thank you, [redacted], for sharing that about your father. I’m sorry to hear that for him--

EN9007: My granddad.

Interviewer: Oh, your granddad, yes, thank you for correcting me. And I wanted to know if you knew anyone in your family who went blind from diabetes.

Reference 5 - 1.40% Coverage

EN9007: Not that I can think of. I know, on my father’s side, they have a lot of diabetes. I just lost an aunt, recently, as far as last week, from complications with diabetes. Yep, all her organs collapsed on her and everything. Yes.

Reference 6 - 1.22% Coverage

Interviewer: Yeah. I see. And do you feel that you can trust your doctors?

EN9007: Yes, pretty much.

Interviewer: Okay. And I guess have you seen your primary care doctor recently?

EN9007: Yes, I have.

Reference 7 - 1.16% Coverage

EN9007: When I found out it, I just tried to do what I can to live as long as I can.

Interviewer: Yes. And--

EN9007: And actually, I have reversed it where I didn’t have to take the needles no more.

Files\\EN9008 - § 4 references coded [ 10.07% Coverage]

Reference 1 - 2.74% Coverage

Well, I know that it’s very important to get a checkup and take your medicine. And one thing I’m behind in, and I don’t really like do my blood test. I don’t do that, so I know that’s important. But I do have a family member that takes their blood sugar and see how much it is or, you know, if it’s low or if it’s too high. And I’m basically learning. I really didn’t want to accept that I had it, so. But I know in my family it runs in I think on my father’s side. I do believe that’s where it came from.

Reference 2 - 1.90% Coverage

Yes, because one time they diagnosed me with it. And I went on a diet and I lost like 100 pounds, but then I gained it back and it came back again. So I guess if I go and lose, lose, lose, lose, lose, lose weight, hopefully that it will go away again. So it really is my sisters, brother, basically the whole family in my _____ [00:13:31] has it.

Reference 3 - 1.84% Coverage

Yes, yes. And I had a doctor, he moved away. He went somewhere else. And someone was in something that he was talking about, and he was talking about me, how I went and lost weight and then how he was so proud of me. But he see me now and I’ve gained some back. So that was nice to know that he thought of me when he was speaking of me. Yes.

Reference 4 - 3.59% Coverage

Well, because like I said, like I can see far away—not far, far, by you know. Okay, like say I’m sitting on my couch and I can look at my TV pretty good. But when I look down, the words and stuff like run together and I really can’t see it. And sometimes when I’ll be reading, it’s really annoying me, and just like the shadow that I have, it annoys me. So I’m really…well, it kind of scares me a little bit because, _____ [00:25:53]. I don’t want to go blind. I want to see. I don’t want nobody leading me around, you know. You want to go one place and you came over here, but I want to see too.4 And I’m concerned, like yeah, that’s the wake-up for me too.

**Annotations**

1 Sub theme we were mentioning. More personal/emotional connection to ophthalmologist. Trust with ophthalmologist? Experience with specific ophthalmologist?

2 This was a very humbling moment in the interview for me.

3 Sub theme again about trust with doctors.

4 Independence, not wanting to inconvenience.

**Resource Availability**

Files\\EN9001_Transcript - § 1 reference coded [ 0.06% Coverage]

Reference 1 - 0.06% Coverage

None of those.

Files\\EN9002_Transcript - § 1 reference coded [ 0.29% Coverage]

Reference 1 - 0.29% Coverage

Nothing early, I’ve always had insurance to get an eye exam. Okay.

Files\\EN9003_Transcript - § 6 references coded [ 6.41% Coverage]

Reference 1 - 0.85% Coverage

Yes. Yes, it happened to me back around 2016, when they stopped it because I had no insurance, and then that stopped me to having an eye exam back then. And then from there to ’18, that’s when the problems occurred in my eye.

Reference 2 - 1.12% Coverage

And then I used to have a treatment on my doctor, private doctor, through that insurance, back then, locally over here. I forget the name. From that to ‘18, when the problems occurred for me, in November ‘18, I was not having any treatment in between it, because it was not affordable for me.

Reference 3 - 0.42% Coverage

You know, so, that’s why, I mean, I was waiting, hoping, to get a new job and then go back to those treatments.

Reference 4 - 1.06% Coverage

They went and picked me up, and then they drove me back to the hospital, emergency room, ER, right here in, where I live. What they did is they just checked on me, and then because I didn’t have any insurance at that moment, so they said they tell me they cannot do anything for me.

Reference 5 - 1.65% Coverage

I mean, they’ve got all the papers when you walk out of the office. You have the sheet with the appointments next to it and the planning. Between my sisters…and then one time I used Uber when I was driving from here because I live 40 minutes away, because I live in [redacted]. That’s a different district. So, it’s like 45 minutes distance. Between my sisters and my mother-in-law, we alternate, and then they drive me down there.

Reference 6 - 1.32% Coverage

I think I haven’t missed much [laughter] since that happened. But I mean, I think if I missed an appointment it was because I had no one to drive me down there, but I don’t think I missed any appointments. I think it was either switching schedule for any other reason, through the building or something happened, but I don’t think we missed any.

Files\\EN9004_Transcript - § 3 references coded [ 3.60% Coverage]

Reference 1 - 1.00% Coverage

So, I think they - I’m not sure what time but I was able to leave by 4:30 and I was - if you don’t get picked up, they won’t come out there and get you. They won’t come back. If they leave you, I’m saying. So, I was just a little afraid I was going to get left. Because the time was ticking, it’s going by.

Reference 2 - 0.48% Coverage

You know, I even used to take the bus - okay, I was at work and had an eye doctor appointment. I had no car; couldn’t drive because I couldn’t see

Reference 3 - 2.11% Coverage

Because my eye problem, I had an eye problem, I couldn’t see. I had to get time out on work. And then, they gave me FMLA. And then, I asked for another extension; they didn’t give it to me and I didn’t know it. They said they reached me but they never did. And they called me in one day with a letter; the next day, they wanted me to come and see them. Thursday, they gave me a letter; Friday, you’ve got to come in. Friday, I don’t know what I’m doing yet. We’ll let you know. Monday, they make a letter that I don’t know about. Then again, on Saturday, knocking on my door, saying resign by so-and-so date and the day had passed.

Files\\EN9005 - § 3 references coded [ 1.69% Coverage]

Reference 1 - 0.48% Coverage

Appointments, really. Mainly, it’s been me.

Reference 2 - 1.08% Coverage

That’s it. Convenience. _____ [00:05:07] passing by or it’s been some place real easy for me to get to.

Reference 3 - 0.13% Coverage

Walk or bus.

Files\\EN9006 - § 4 references coded [ 2.57% Coverage]

Reference 1 - 0.97% Coverage

All we have to do was basically seek the information. It’s readily available. You just have to be seeking it.

Reference 2 - 0.15% Coverage

I have a vehicle.

Reference 3 - 0.67% Coverage

A lot of to and from, and way overcrowded, obnoxious people on the city bus.

Reference 4 - 0.79% Coverage

You need to because it’s bad enough I lose my mobility. I’m a diabetic. You need to visit.

Files\\EN9007 - § 6 references coded [ 17.44% Coverage]

Reference 1 - 0.80% Coverage

EN9007: Yeah, so I would say yes.

Interviewer: So which ones seem to resonate with you?

EN9007: Not really having transportation.

Reference 2 - 2.97% Coverage

EN9007: Well, they got now-- they have, because of COVID, I can get a ride now through my insurance. Before, they didn’t have that. So my funds wouldn’t last me on different days I had to show up for this appointment. So I couldn’t make it there.

Interviewer: So how did you get to appointments before COVID, and how do you get to appointments now?

EN9007: The bus. I would take the city transportation when I was able to get on a bus. Right now, I’m in so much pain I can’t even ride a bus, young lady.

Reference 3 - 3.55% Coverage

Interviewer: And so you use a ride share service now?

EN9007: Yes.

Interviewer: Yes.

EN9007: Veyo, it’s called, now.

Interviewer: Okay. You said it’s Video?

EN9007: Veyo. That’s the name of the transportation department now.

Interviewer: Okay, Veyo. And how has that experience been?

EN9007: Well, they’ve been messing up on different dates that I have to be at an appointment. And they say-- I call them like I’m talking to you now and set it up with them on the phone. Why the driver don’t know where, half the time, that I am right now. And it caused me to miss out on some appointments.

Reference 4 - 5.12% Coverage

Interviewer: Okay. So it seems like transportation definitely resonates with you. I wanted to know-- again, and other examples have been not having secure housing, so being homeless or being incarcerated.

EN9007: Yes, yes, yes, yes. I’m homeless as we speak but I have somewhere to be at for now. But I am definitely homeless.

Interviewer: I’m sorry to hear that. How have you balanced not having housing with going to appointments?

EN9007: It’s difficult, but I manage.

Interviewer: Have there been any tools that have helped you, whether that’s shelters or family members?

EN9007: I refuse. I refuse to go in anybody’s shelter because I had a bad experience there once before. All of my belongings was taken away, stolen. And I addressed the problem. If I got too upset they were saying I’m aggressive and all of that. I don’t got time for that.

Reference 5 - 2.06% Coverage

EN9007: Well, just I wasn’t able to get there, that’s all. If I would have had somebody to come bring me, I would have been there. And then I have to go through stress of asking somebody to bring you, have to deal with they attitude and whatever they was supposed to be doing at the time. I don’t want to go through that. That’s why I didn’t make it.

Reference 6 - 2.94% Coverage

Interviewer: No, that’s okay. I guess, overall, do you think of it as a positive experience or a negative experience?

EN9007: Oh yeah, I think of it as a positive, because they’re trying to help keep my eyes strong and safe.

Interviewer: Yeah. And so what do you think has prevented you from going to eye appointments every year?

EN9007: Say again?

Interviewer: What do you think has prevented you from going to eye appointments every year?

EN9007: Again, I would say transportation.

Files\\EN9008 - § 6 references coded [ 8.01% Coverage]

Reference 1 - 2.54% Coverage

I think mine were I didn’t really _____ [00:05:22]. Oh, gosh. I just was going, then all of a sudden I stopped when my…it was kind of hard for me when my husband was sick and couldn’t get around. So when I did go…I went when I could, but if I had to take him or let him go to the doctor or something, I didn’t have a way. So all of a sudden, I just fell back in going. Now I know that it’s really important for me to get my eye exam, especially with diabetes and stuff.

Reference 2 - 2.29% Coverage

Yes. And another thing, I didn’t have the insurance at that time too. I didn’t have the insurance because I didn’t have full Medicaid. I had the one with the red and blue card, but I didn’t have the statement insurance. It was kind of hard like even…I know we we’re just talking about the eyes, but even with dentists, I always ended up paying a lot of money out of my pocket to go to the dentist, like over $1,000.

Reference 3 - 0.49% Coverage

Yes, like I didn’t have the money at that time. I had to pay for everything out of pocket.

Reference 4 - 0.80% Coverage

No. Really, no. Only thing I can think of that I said one time I didn’t have insurance. That stopped me a lot, and I had to pay out of my own pocket.

Reference 5 - 0.81% Coverage

So we had to pay for our own, and he was back and forth to the doctor a lot. Sometimes he had to pay…like his medicine sometimes would be over $300.

Reference 6 - 1.07% Coverage

They got their fund that they’re not supposed to refuse nobody if they need help. But thank God we made it. I got my Blue Cross Blue Shield right now until they switch me over. So I’m doing good.

**In-Clinic Experiences**

Files\\EN9001_Transcript - § 8 references coded [ 13.15% Coverage]

Reference 1 - 0.50% Coverage

I would ask him a question while we were in the hospital and the next day he will come back and answer the question.

Reference 2 - 0.50% Coverage

every time I went he was giving me laser surgery. Since like, when I left him my I was worse than it was when I went in

Reference 3 - 1.61% Coverage

I went to [redacted]. And there [redacted]. Now I’m just being on Chanel. Okay. Yeah, when I first got there, the doctors at Yale the first day was just not really nice and kind. They wouldn’t explain them to it to me properly and acceptor. And I was getting in an uproar. Right. And, and in comes, this lady who’s a doctor, and she has one. She had one of the cheeriest.

Reference 4 - 3.21% Coverage

So yeah, his name is [redacted]. Okay, that was his name. When I was first going. She had one of the best purchase attitudes I have ever, ever, ever run across, you know, she talked to me, like I was a little boy. And seriously, and she just she, you know, she, I got relaxed, like calm down. And she talked to me and went through what we need to do how we need to do it. She pulls things up on the computer, this is what swollen, and this is what we need to bring down. You know, and so that’s what’s really got me faithful. And I’m being switcheroo faithful to going to [redacted], and getting my eyes looked at, you know, I don’t get to see her all the time, you know, but she was in initial impact, you know, of to the kindness and the cetera.

Reference 5 - 0.12% Coverage

you felt the someone cared.

Reference 6 - 0.77% Coverage

But the home base, someone cared, you follow me? Yeah. You know, that’s, that’s what bored? at home, and this got me going. And I rarely miss an appointment. I rarely miss one.

Reference 7 - 4.15% Coverage

But I don’t know if anything could have made it quote unquote, better. I’d say I can’t get I can’t get any better than what the doctor did. By by sitting down. by sitting down and giving me respect. And really showing that she cared was the best that it could be. You know, because by her care, and showing she cared, made me honestly start caring about me. You know that there is a chance you know, you know, we can get better? Because there’s some days she’ll tell you I’ve been there. And she look at the computer. Well, you know, we need to do laser surgery and the needle in your eye. That’s a different thing they call the name, you know, I get to looking at it, you know, cuz I hate needles. Oh, don’t worry about it. You know, it ain’t gonna hurt, you know, just a pinch, you know, you know, and so she, you know, she made it just so comfortable and relaxing. You know, and you felt she’s caring about you. You’re damn fool not to care about yourself.

Reference 8 - 2.29% Coverage

Yeah. Because when you go places and you find people with not nice personality, or attitude, you know, it kind of tends it. You don’t even want to go back. Right? You know what I mean? Your wife or husband say you got an appointment tomorrow, but I don’t even feel like I’m not going you know, yeah. And you’ll go through your troubles rather than go because, you know, they don’t even care. You know, so that’s why I don’t think that you know, anything could have they could have done anything better than than what she did.

Files\\EN9002_Transcript - § 5 references coded [ 4.42% Coverage]

Reference 1 - 0.72% Coverage

Good, fine. I like to clinic, you know, I really won’t see anybody else but Dr. [redacted]. You know, unless it’s absolutely necessary, but I’m fine with the clinic.

Reference 2 - 0.96% Coverage

I didn’t see any bad thing about it. We hit it off. And the first time I walked through the door, we had, you know, me, her, her assistants and the nursing staff. Very good. nothing bad to say about any of them. They’re good.

Reference 3 - 0.59% Coverage

Don’t let anybody play with my eyes. trust her. Yes. to the fullest. You know, so when they told me she was out there, that’s where I went.

Reference 4 - 0.17% Coverage

I won’t let anybody else touch my eyes.

Reference 5 - 1.97% Coverage

I think she’s an all around good person. She’s good spirit is very happy. He enjoys what she does. And she takes the time out to enjoy her patients like me. Wonderful. So you found that she was making the time she made the time for patients like you. And yeah, I can hear her when I go and visit how she reacts with other patients and how they react with her. Because you know, she is kind of loud. So her voice does carry. She’s She’s just a complete sweetheart.

Files\\EN9003_Transcript - § 3 references coded [ 6.37% Coverage]

Reference 1 - 1.97% Coverage

Because of the way the doctor…I mean, I don’t blame at all, but I think there was a short explanation from the doctor to the patient and let me understand the situation that I was going through, because all I’ve seen, they’d given me a treatment, and they tell me, we’re doing this because we don’t want you to lose your vision. Okay. I understand that, but for me at that time, having 20/20 vision, I couldn’t understand the hardness, I would say, that I was running. …oh, hold on, let me just answer this real quick.

Reference 2 - 0.82% Coverage

So, I just went there, and I told them…this is the department for students. So, they start working on it, but I don’t think they understand what I say, or they don’t want to listen. So, they are students and teachers

Reference 3 - 3.57% Coverage

It was so high, it was 69. So, and then, he just asked me permission to poke the eye with a needle and take the fluid off. So, in that time, so he has the permission for whoever’s running that department out there, and that’s when they took me down here to [redacted]. He brought me down there, and then he helped me out to sign all the paperwork to get insurance and everything…but then I guess it was too late when they brought me there, because when they did the laser, and they started from that time, it took me to [redacted], it went on three more weeks before they did the first surgery on my eye. So, all that time, the eye was clouded. So, he was asking me to go there to see him, I mean, twice a week, so he was poking the fluid of the eye with the needle until they did the lasers and the stuff, and then that’s when I started to see that I had all these problems, and then I started treatment again through you guys out there, at [redacted].

Files\\EN9004_Transcript - § 10 references coded [ 16.77% Coverage]

Reference 1 - 0.09% Coverage

They didn’t, they didn’t.

Reference 2 - 2.27% Coverage

No, I was at - where was I? I was at - was I at [redacted] ? I was at a doctor’s office. She told me I had diabetes. She said nothing about it except, “You’ve got diabetes and you have to start taking pills,” at that time. And I believe - I wasn’t even told that you don’t have to take the medication. You could even try to like start eating correctly and not get on anything. No, first, right away, I was put on metformin, whatever it was back then. Glucophage - yeah, Glucophage back then, yeah. She told me nothing about my eyes or anything. And diabetes runs in my family. And it seems like someone would’ve told me to eat better or whatever or whatever but I wasn’t told.

Reference 3 - 4.87% Coverage

Okay. That’s, you know, at first, and then, second. When I went there - well, I don’t want special attention. I just want to be treated like I’m a person. Okay, at first, the nurses I had with Dr. [redacted] were always great. Always, always great. But it seems like the nurses I get with Dr. [redacted], because I don’t know them, sometimes they can be a little rude-ish. Not rude. Like the other day, I was there and I was waiting and I have _____ [00:18:01]. So, I only had a limited amount of time and the time was going, going, going. And they brought me in and said, “You’ve got to be waiting in another room to be seen.” I said, “Fine.” So, I’m sitting out there waiting, waiting, waiting. Everybody’s getting called in except me. So, I said, “What’s wrong? Why am I not called?” “Well, there’s a lot of people ahead of you but I see where you are.” And I said, “I’m sure Dr. [redacted] wants to see me.” You know, he was going to do an injection in my eye that day but he didn’t. So, I said, “But I can’t be seen, I’ve got to come another day and I don’t really want to do that.”

So, another nurse came - the one was gone, that one, and another one came back and she brought me in the room. And I said, “Sweetie, why do you bring me in? Because they told me that they’re going to bring me in when Dr. [redacted] was done.” And she said, “Oh, if you don’t want to be in the room, somebody else can come in the room.” And I thought that rather rude.

Reference 4 - 1.88% Coverage

Residents, right, came in and she did my eye. And she looked - or he, whatever it was, I forget. The older lady, she was really nice. And she looked in my eye and then, Dr. [redacted] came in and said what he said.

But my point was why Dr. [redacted]’s nurses always seem - or that side are always nice. And you go in Dr. [redacted]’s side, you be like, “Huh?” Because it was just most of them are - all the nurses I had with Dr. [redacted] were great. But on the other side, they’re not. You know, they’re a little iffy. It’s the way they talk to you sometimes.

Reference 5 - 1.65% Coverage

Yes, for the ride home, right. So, that’s all I explained to them when she said, “Well,” whatever, “A lot of people ahead of you,” that was one person. And there’s the other person in the room, I’m like, “What’s going on?” You know. So, I said, “Well, if I can’t be seeing him, I have to tell the doctor that I’m going to have to go.” They didn’t tell him, they didn’t tell him, no. Yeah, but he saw me, he saw me. And I’m going to see him again the 30th, maybe get a needle in my eye, who knows?

Reference 6 - 1.62% Coverage

Oh, no. Oh, no. Because you know what? I try to be civil with my mouth because sometimes I can be a little curt; sometimes be a little out of the way. So, that’s why I was being really calm talking to her in the office, in the room.

So, no, I’m going to never not go but I’m going to have to go pretty - if they do it again, I’m going to have to go somebody’s - or whoever and explain to them that they’re doing that to me. No, it’s not going to never stop me from going to the eye doctor, no.

Reference 7 - 1.63% Coverage

My eyes, my eyes. That’s my eyes, that’s not their eye. They don’t care about what happens to my eye. I do. So, why would I not go for an eye docto1r’s appointment where I love the doctors? I love Dr. [redacted] and Dr. [redacted], I really do. And I told whoever my insurance people were, “I want them. I do not want to change where I go for my eye doctor. I don’t want to do nothing. If I can’t go to them, I’ll pay for it myself,” you know? I just like Dr. [redacted] and Dr. [redacted].

Reference 8 - 0.29% Coverage

But at this point, you want to see them. Like you don’t want to change.

EN9004: Never.

Reference 9 - 1.23% Coverage

I’m going to say this - excuse me - I’ll say this. One day, Dr. [redacted] was gone on a Friday and someone came and took her place. I’m like, “Who is this? I don’t want to see them. Why am I here if she’s not here?” [laughter] I think - I don’t know what I did. I was really snotty; I know I was. But because if it’s not Dr. [redacted], don’t have that appointment for me.

Reference 10 - 1.23% Coverage

I couldn’t see - an eye doctor appointment to see Dr. [redacted] that day and that particular day in 2018, I saw her. She told me I didn’t sound right, I didn’t look right, and so, she called for the ambulance, told me, “Go to the hospital.” And I said, “I’m not going in the other _____ [00:26:21].” They all said, “Oh, yes, you are.” So, I went and I had a stroke.

Files\\EN9005 - § 1 reference coded [ 2.10% Coverage]

Reference 1 - 2.10% Coverage

I just haven’t been to one in about two years and it had to be really convenient. I got my eyes checked. They checked my eyes. They did the recommendations and that was it. There’s nothing more than that.

Files\\EN9006 - § 5 references coded [ 6.56% Coverage]

Reference 1 - 3.36% Coverage

Of course, that A1C level. We had to get it down below 6.5 and it was over 7 by then. It affects what you see. Diabetes is not good period. You have to watch what you eat. You have to watch what you do. Exercise is required. You don’t want to become dependent on medication. You need to do something to change your situation. So, you have to use physical exercise—walking, something.

Reference 2 - 1.62% Coverage

You go in, they dilate your eyes. They look at your eyes through that thing. The new technology, they’re very informative and I ask questions anyway. So, it was just a walk in the park.

Reference 3 - 0.20% Coverage

No, I go wherever he is.

Reference 4 - 0.13% Coverage

Oh, it’s fine.

Reference 5 - 1.25% Coverage

They’re very informative. They tell you about whatever you needed prior to and they walk you through it. So, there isn’t any mystique to it.

Files\\EN9007 - § 3 references coded [ 5.56% Coverage]

Reference 1 - 3.61% Coverage

Interviewer: Okay. What did you see the eye doctor for?

EN9007: I forget. I think some type of cataracts. They say, I believe-- I’m not even for sure what they were seeing my eyes for. I really don’t remember. But I know they are giving me dilation, when they drop these drops in your eyes to look at your pupil, if I’m saying it right?

Interviewer: Yeah, pupil, yep.

EN9007: Yep. But I know I’m in the computer. And I used to go down on [redacted]. Actually, that’s the last time I went to the eye doctor, on [redacted].

Interviewer: On [redacted], okay. So you were saying that’s the last time you went.

Reference 2 - 1.22% Coverage

Interviewer: Yeah. I see. And do you feel that you can trust your doctors?

EN9007: Yes, pretty much.

Interviewer: Okay. And I guess have you seen your primary care doctor recently?

EN9007: Yes, I have.

Reference 3 - 0.72% Coverage

Interviewer: Okay, perfect. You were talking about your primary care doctor.

EN9007: Yes. Very nice lady. I like her.

Files\\EN9008 - § 3 references coded [ 5.65% Coverage]

Reference 1 - 2.83% Coverage

Good. Every time I go…when I go there, everybody remembers me sometimes. But now it’s been so long apart, that maybe every now and then somebody will remember me. But back in the old days when I used to go, everybody remembered me. Hi, [redacted]! Hi, [redacted]! So I really, you know, takes getting used to. Of course, I’m not used to calling it…well, we called it [redacted]. I’m just used to going to [redacted]. So everything is nice. Every time I get a doctor, he is she is real nice, including you.

Reference 2 - 0.99% Coverage

Not really. I really have not. Like I said, everyone always treated me nice and respectful. I never had a bad experience, not with a nurse or the doctor. Everyone’s been good.

Reference 3 - 1.84% Coverage

Yes, yes. And I had a doctor, he moved away. He went somewhere else. And someone was in something that he was talking about, and he was talking about me, how I went and lost weight and then how he was so proud of me. But he see me now and I’ve gained some back. So that was nice to know that he thought of me when he was speaking of me. Yes.

**Annotations**

1 Sub theme again about trust with doctors.

**Cues to Action**

Files\\EN9001_Transcript - § 4 references coded [ 2.85% Coverage]

Reference 1 - 0.58% Coverage

And I went back to the same hospital emergency room. And that’s when they said, hey, you’re a diabetic. And that’s when they kept me.

Reference 2 - 0.26% Coverage

But we don’t pay attention until things start turning bad.

Reference 3 - 1.30% Coverage

I started to twitch a lot. Okay. And that’s when I sought out eye doctor. And so they were giving me Give me the help for that. When they do your eyes when you put it in that machine. And ah, it’s like that vision machine where they take pictures. Know, where they were zapping the blood out your eyes.

Reference 4 - 0.70% Coverage

And I went to an eye doctor, and that’s when, you know, he told me I needed laser surgery. And so I started just going there. And, and that’s the beginning of it.

Files\\EN9002_Transcript - § 8 references coded [ 5.22% Coverage]

Reference 1 - 0.06% Coverage

Reminders...

Reference 2 - 0.41% Coverage

Yeah. You know, they call you a day or so ahead of time and let you know, you have an appointment.

Reference 3 - 0.16% Coverage

I had little blood vessels in my eyes.

Reference 4 - 0.80% Coverage

I’ve always had diabetic eye exams. But when the blood vessels started coming in, that’s when I go more often and went to that’s when that’s when I first started saying Dr. [redacted].

Reference 5 - 0.19% Coverage

I’m just trying to keep up with new frames.

Reference 6 - 0.22% Coverage

Well you know, I didn’t know until that happened.

Reference 7 - 1.65% Coverage

I was like seeing I don’t know. It was like, seeing something in your eye. I thought something was hanging from my head. And nobody else could see it. But me so I figured it was on the inside. So I went to an eye doctor and they told me that I had some whatever it was blood vessels that popped or whatever. And that’s what I like to see Dr. [redacted] and they did a laser surgery on my eye.

Reference 8 - 1.72% Coverage

if it wasn’t for that, I probably wouldn’t have gone when I did. But on a regular I was go because you know, I had the insurance that they were paying for. So I went every year. Right? But if it wasn’t for that, I was as a matter of fact, I was at work and it just popped up all of a sudden I like I said, I thought it was a fly flying around my head and nobody else work could see it. And it started getting worse.

Files\\EN9003_Transcript - § 4 references coded [ 19.94% Coverage]

Reference 1 - 16.29% Coverage

Oh, when that situation, when it happened that day. It was a situation that I worked…I went to work with this company that I worked overnights. I mean, understand, my diabetes, it was related to my vision. It was hurting my vision. So, I worked the shift straight, nightshift, and the next day, I had to wake up early in the morning. So, I took a day in the middle.

So, on my way to go to work, like 5:00 o’clock in the morning, I wake up, I was driving it, and all of a sudden my eyes started getting really blurry, and then all of a sudden I felt the light that was coming at me, oncoming traffic. I was seeing it was a really huge bright light, and like realizing, I was like, this is not normal. This is something happening, and then all of a sudden, I noticed that I was not reaching far, that my distance of seeing, and I was like, okay, something is wrong on my eye because I don’t see any of the traffic…usually regularly seeing it far away.

And all of a sudden, I saw like…I call it water, but I think it was tears. I mean, that’s what I thought it was…when you put water, right, on a window, and then you see that water the first drops coming down, I just saw that in my both eyes, not on the left eye only. Actually, on both eyes. And then all of a sudden, I didn’t understand that, nothing about high pressure inside the eye, nothing, like I said before. I didn’t have education for that.

All of a sudden, I just started seeing more cloudiness, and then I just pulled over, and then I was like, in my head, I was thinking that I was tired from that shiftwork the couple days before. So, I just pulled over and then rest, you know, for about an hour. So, when I wake up for the noises around me and try to see, and then all I was seeing, it was a big cloud in front of me, not even seeing the hood of my car. So, all that, I mean, rushed back. I mean, I called my father-in-law. They went and picked me up, and then they drove me back to the hospital, emergency room, ER, right here in, where I live. What they did is they just checked on me, and then because I didn’t have any insurance at that moment, so they said they tell me they cannot do anything for me.

So, long story, short story, I just end up going to, I mean, a day after, [redacted] University. Somebody tells me, listen, if you go up there, then tell them that you have no insurance at all. They’re going to take care of you. You need to go there. So, I just went to the ER in [redacted], and then one of the doctors…I don’t know if it was a practitioner or the doctor…they put eyedrops on me and my vision went back. So, he told me, you need to see your doctor immediately because you have a problem with your eyes. If you don’t treat this, you’re going to have a lot of problems. And that day, my vision went back to normal. I just walked out there, driving my car myself. I mean, even with drops on my eye, I could drive myself back home, that day.

But I was like, okay, this incident happened. I mean, so, I took a day off after that to start to make the arrangement to go back and see the regular local doctor, but they were so booked up, there was nothing. They couldn’t handle seeing me so soon.

So, in the days after, that increased again. So, I just ran out. I went back to the ER again in [redacted]. That’s when they told me and sent me where they have a Department out there, where they treat me, giving me a treatment. And then one of the doctors, I heard him, he told one of the students, he said, listen, he’s got a clot at nine o’clock here and then he told me, “Well, you need to go to that department and tell them that you need to have lasers done before your eyes get worse.”

So, I just went there, and I told them…this is the department for students. So, they start working on it, but I don’t think they understand what I say, or they don’t want to listen. So, they are students and teachers…and I don’t know what happened. So, just one doctor…I don’t remember his name…I forget his name. I know it was Ian. So, when he reads the chart, he tells me, “You’re going to be blind,” and I was like…I started crying. I went in tears. And then at this moment, my right eye…starts seeing, but my left eye clouded up. And they told me, if you don’t do anything, your eye is going to pop out, going to blow out. I had a 69 pressure on that eye, on the left eye.

Reference 2 - 1.65% Coverage

I mean, they’ve got all the papers when you walk out of the office. You have the sheet with the appointments next to it and the planning. Between my sisters…and then one time I used Uber when I was driving from here because I live 40 minutes away, because I live in [redacted]. That’s a different district. So, it’s like 45 minutes distance. Between my sisters and my mother-in-law, we alternate, and then they drive me down there.

Reference 3 - 0.68% Coverage

No, no, I can remind myself, and then they remind me too. You know, they pay attention on that, so they’re aware about it, even my wife. So, we all, all the time, we keep an eye on it.

Reference 4 - 1.32% Coverage

I think I haven’t missed much [laughter] since that happened. But I mean, I think if I missed an appointment it was because I had no one to drive me down there, but I don’t think I missed any appointments. I think it was either switching schedule for any other reason, through the building or something happened, but I don’t think we missed any.

Files\\EN9004_Transcript - § 3 references coded [ 2.67% Coverage]

Reference 1 - 0.90% Coverage

No, no, no. I just remember my eye doctors. I have several but I always remember my eye doctor appointments. Hers is next week or the week after; it’s the Friday next week. And I have one with Dr. [redacted] on the 30th. So, I just remember the eye appointment. Yes.

Reference 2 - 0.57% Coverage

Yes, yes. Ever since the day I had my operation; ever since then, to one day - four days a week to three days a week to two days a week - made you remember that you have to go.

Reference 3 - 1.20% Coverage

That’s when I thought I had an eye problem. That’s why I know. My eye was - when I first found out - was 50 in my eye. It was pain in my eye and I had to go to the emergency room. And it just so happened I didn’t know him them; Dr. [redacted] was one of the doctors. He was also at that - turned to be my - whatchacallit - surgeon. Yes, and then, to be my doctor after.

Files\\EN9005 - § 5 references coded [ 3.33% Coverage]

Reference 1 - 0.43% Coverage

I didn’t set an appointment up. I forgot.

Reference 2 - 0.79% Coverage

Yeah, what has helped me was it probably set for me and someone reminded me.

Reference 3 - 1.08% Coverage

That’s it. Convenience. _____ [00:05:07] passing by or it’s been some place real easy for me to get to.

Reference 4 - 0.89% Coverage

I said it must have affected my eyes at one point in order to have my eyes checked _____

Reference 5 - 0.15% Coverage

Convenience.

Files\\EN9006 - § 4 references coded [ 2.63% Coverage]

Reference 1 - 0.54% Coverage

So, I keep them as current issues. They were priorities, so.

Reference 2 - 0.09% Coverage

My Chart.

Reference 3 - 1.19% Coverage

It’s not on my phone now because when I changed phones I can’t get it. I don’t know how it works with all that. I’m not all that tech savvy.

Reference 4 - 0.82% Coverage

I do have the color note app though, so at any point in time I just immediately put it in there.

Files\\EN9007 - § 2 references coded [ 3.08% Coverage]

Reference 1 - 2.08% Coverage

EN9007: Yes, I mentioned MyChart, I know that.

Interviewer: Yes. So it seems like it’s been very helpful for you.

EN9007: Oh yes. Yes, it has.

Interviewer: Yes. So I wanted--

EN9007: I’ve got all my images on there, because you know I had surgery. I had a brain tumor in 2012. They operated on me. And then I had a kidney removed, just this May past.

Reference 2 - 1.00% Coverage

Interviewer: I see. And how long have you been using MyChart?

EN9007: Oh, for a while, while. Over three, four years, if not more.

Interviewer: That’s great to hear.

Files\\EN9008 - § 3 references coded [ 3.38% Coverage]

Reference 1 - 1.72% Coverage

Well, when I start seeing like my…I haven’t really had an eye exam in about at least five years. And then I noticed I can see far off, but when I look down, it’s hard. The words like go together. And I need to change my eye glasses, because the ones I have, I’ve had those about eight years, so _____ [00:09:01].

Reference 2 - 0.87% Coverage

Well, I don’t know if you remember, I talked to you I think. When I’m looking straight, it’s okay, but when I look, I could see a shadow coming around my eye.

Reference 3 - 0.79% Coverage

I mostly remember it. Or if I can’t remember, I’ll call and ask when is it so I won’t have you waiting and I’m not coming in. You know how that is.

**Knowledge-Creating Experiences**

Files\\EN9001_Transcript - § 3 references coded [ 2.12% Coverage]

Reference 1 - 0.49% Coverage

You know, and you know, so one doctor wind up telling me that it’s genetic. It’s something inside your system.

Reference 2 - 0.58% Coverage

And I went back to the same hospital emergency room. And that’s when they said, hey, you’re a diabetic. And that’s when they kept me.

Reference 3 - 1.05% Coverage

And my sister’s a diabetic. I used to go over her house. She was taking pills at first, and she wasn’t eating anything with sugar in it at all. And you know, I’ll be over a house. She’s older than me. And she wind up still getting on the needle.

Files\\EN9002_Transcript - § 2 references coded [ 1.59% Coverage]

Reference 1 - 0.33% Coverage

I don’t even remember, it’s been so long ago. I just do what I have to do I take.

Reference 2 - 1.26% Coverage

No, I had my experience, I let it go. So if it happened, it happens. You know, whenever it comes back, like I said, Now, sometimes it comes back and it disappears before I can even get to my appointment. So the last time I went was seeing Dr. [redacted]. And it had already cleared up on its own.

Files\\EN9003_Transcript - § 5 references coded [ 20.50% Coverage]

Reference 1 - 0.62% Coverage

. So, he told me, you need to see your doctor immediately because you have a problem with your eyes. If you don’t treat this, you’re going to have a lot of problems.

Reference 2 - 7.21% Coverage

Thanks to the Internet, YouTube, might as well, because being…like I told you before, I mean, from being a truckdriver and being on the street, and after this incident happened to me, kind of blind by myself…I mean, kind of blind. So, I had to find out something to help me to entertain myself and keep my mind busy and try to focus on something because it was a shocking moment. I’m sorry. [voice cracking] I’m sorry. It was a shocking moment, you know, so, crash, my life. I’ll say, this was a crash on my life.

So, being someone that was always willing to help and work…I had to focus on something that was…and I’d like to understand the study and be aware of where diabetes disease came from, and then all the eye diseases are from, and how exercise and how to reverse all this, how that impacted me.

So, YouTube, they have a lot of crap, [laughter] but they have a lot of good, really good information about it, like colleges, universities. They’re speaking and they have real facts and education. So, from some of those videos, I’ve been taking what I think is helpful for me. Because like I said, some other ones, I don’t even understand what they’re talking about, or I don’t care about it, and some of them are really fake, and they are more business and trying to sell products, stuff like that. So, they have some other ones, like [redacted] University. They really work really good on that, on stem cells and stuff like that and education about macular degeneration disease, diabetic retinopathy. And then they have another university in [redacted]. I forget the name of it right now. And they have another hospital in [redacted], where they work on all this, and they help the people on diabetic control and diet control to improve the eye health and then the diabetes itself.

That’s where I get more of the information. I really try to understand and educate myself.

Reference 3 - 1.93% Coverage

I’ve got four sisters. They all have diabetes. Before I used to be the one with the worst diabetes, and right now, just for you to know, I mean, my diabetes was 14 up to 16, my A1C, before this happened. It was really way out of control. They never put insulin on me. But after that, learning how to control the pancreas, the fluid, how to control myself in food and some other interests. So, right now, I’m the expert. My sisters, I’m helping them when their diabetes is out of control. My A1C right now is 6.3.

Reference 4 - 1.71% Coverage

Yeah, that’s what I’m becoming now. Now, when they’ve got a problem with their sugar and they are too high…I mean, when you understand how your body works, it’s so much easier to understand it. I mean, when you’re fasting, after night, and how the vegetables and the natural foods and how you prepare stuff like that, you almost don’t need medication no more because your body starts working by itself, little by little, and it’s getting back.

Reference 5 - 9.03% Coverage

Before, I felt like I was a number. I was another number, not even a patient. I mean, for some of the clinics where I used to go before, because I was asking questions and then not really many right answers. So, right now, Dr. [redacted], I’ve got to tell you, I love her. I’m not going to change. She has the best attitude.

Some of the doctors out there, I mean, I have to tell you the truth, some of those other doctors that’ve been helping her, they’re really good, but they have one or two of them, they need to have better concern in their careers, and change their careers because they don’t know how to handle patients, you know.

Dr. [redacted], she takes the time to talk to me and make me understand the problems that I’m going through. Sometimes I’m asking questions and she tells me, “You know the answer, EN9003.” [laughter] You already know. Yeah, like, okay, because I want to hear it from the expert. She’s the expert, right. [laughter] She will tell me and then repeat it again and then she goes back, and she shows me pictures, like right now for the injection. When I was kind of confused to have the injection, I mean, I’m being honest to you, to have that injection in the eye every month, and she makes me understand it. I mean, we’ve got to keep it under control.

Because, I mean, I was kind of disappointed to have the same treatment monthly…and I just told Dr. [redacted], “Listen, I don’t want this anymore because for two years and being through this, you think I can put an injection on me and have it making no changes.” So, even with the diet I’m using now, I have more control. I understand it there. If I have control, my diabetes, and all the stuff I’ve been doing, plus the injections, yes, it’s coming down, little by little. It’s not happening big in the eye, but it’s coming. I mean, I see improvement, little by little, but it’s coming.1

I had a good experience with the office out there at [redacted] and some other ones, but before that, I mean, some of the doctors…I don’t know if they don’t want to waste their time because they need to quota with the numbers of patients, and then they just want to wipe off the office, and then listen, I need to do what I need to do, and you just come back next month. I mean, I didn’t understand that. I did not understand that.

But right now, it seems like it is a good experience, the way I am right now.

Files\\EN9004_Transcript - § 5 references coded [ 5.95% Coverage]

Reference 1 - 0.09% Coverage

They didn’t, they didn’t.

Reference 2 - 2.27% Coverage

No, I was at - where was I? I was at - was I at [redacted] ? I was at a doctor’s office. She told me I had diabetes. She said nothing about it except, “You’ve got diabetes and you have to start taking pills,” at that time. And I believe - I wasn’t even told that you don’t have to take the medication. You could even try to like start eating correctly and not get on anything. No, first, right away, I was put on metformin, whatever it was back then. Glucophage - yeah, Glucophage back then, yeah. She told me nothing about my eyes or anything. And diabetes runs in my family. And it seems like someone would’ve told me to eat better or whatever or whatever but I wasn’t told.

Reference 3 - 2.17% Coverage

Okay, I have several answers to that question. I didn’t seek out any more information. I have three of my family; my father’s mother, sister, my father’s sister, and my father’s first cousin. All three of them had lost their sight. And now, my father’s first cousin, also, had lost his sight. They’re all from diabetes. So, I didn’t seek out any information because that’s what’s scaring me. It’s crazy to say but what happened to them, I was afraid that if they examined my eyes, they would tell me that. But it ended up that way anyway.

So, you know, I thank God I didn’t go that way. What I’m saying is I was like that until my surgery.

Reference 4 - 0.91% Coverage

If I want to know something, I have Dr. [redacted] at [redacted] or Dr. [redacted] . And I can’t read to see - I’m reading better now but I couldn’t read to see anything. So, I couldn’t - and my phone doesn’t do it so, I couldn’t get information. I couldn’t get it anyway.

Reference 5 - 0.51% Coverage

And they didn’t tell me but I’m assuming that it was diabetes.

Interviewer: I see. So, if anything, that added to the fear that you were mentioning.

Files\\EN9006 - § 7 references coded [ 8.86% Coverage]

Reference 1 - 0.97% Coverage

All we have to do was basically seek the information. It’s readily available. You just have to be seeking it.

Reference 2 - 3.36% Coverage

Of course, that A1C level. We had to get it down below 6.5 and it was over 7 by then. It affects what you see. Diabetes is not good period. You have to watch what you eat. You have to watch what you do. Exercise is required. You don’t want to become dependent on medication. You need to do something to change your situation. So, you have to use physical exercise—walking, something.

Reference 3 - 1.41% Coverage

Well, I had spoken to my primary care and then I read all the pamphlets that they give you. A person with diabetes _____[00:07:14] as well. And they can read it.

Reference 4 - 0.23% Coverage

Reading’s fundamental.

Reference 5 - 1.01% Coverage

--or not? Okay. So, you wanted to know more about diabetes—how it could affect your organs?

EN9006: Absolutely.

Reference 6 - 0.65% Coverage

Reading is fundamental, no. It’s not hard to find information about it.

Reference 7 - 1.25% Coverage

They’re very informative. They tell you about whatever you needed prior to and they walk you through it. So, there isn’t any mystique to it.

Files\\EN9007 - § 3 references coded [ 8.37% Coverage]

Reference 1 - 3.26% Coverage

Interviewer: Okay. And do you remember what that experience was like?

EN9007: I think I was having like a lot of sweats at the time, and I was told that I had a lot of sugar.

Interviewer: Were you told anything else?

EN9007: No. If I did, I don’t remember, young lady.

Interviewer: Yeah, no, that’s okay. That was a while ago. So that’s more than understandable. I wanted to ask if you were ever told that diabetes could affect your eyes.

EN9007: Oh yes. Your eyes, your limbs, your hands, your fingers. Amputations. I already know all of that.

Reference 2 - 3.71% Coverage

EN9007: Well, I go back-- because my granddad had diabetes. And what ended up happening to him, he had to do dialysis, at the end of the day. I’m trying not to do that. How could I explain this? Yeah, I’m just trying not to do what he did, which is go on dialysis and he soon gave up and gave in. Now he’s in Heaven. I don’t want to go through that.

Interviewer: Thank you, [redacted], for sharing that about your father. I’m sorry to hear that for him--

EN9007: My granddad.

Interviewer: Oh, your granddad, yes, thank you for correcting me. And I wanted to know if you knew anyone in your family who went blind from diabetes.

Reference 3 - 1.40% Coverage

EN9007: Not that I can think of. I know, on my father’s side, they have a lot of diabetes. I just lost an aunt, recently, as far as last week, from complications with diabetes. Yep, all her organs collapsed on her and everything. Yes.

Files\\EN9008 - § 2 references coded [ 4.24% Coverage]

Reference 1 - 2.74% Coverage

Well, I know that it’s very important to get a checkup and take your medicine. And one thing I’m behind in, and I don’t really like do my blood test. I don’t do that, so I know that’s important. But I do have a family member that takes their blood sugar and see how much it is or, you know, if it’s low or if it’s too high. And I’m basically learning. I really didn’t want to accept that I had it, so. But I know in my family it runs in I think on my father’s side. I do believe that’s where it came from.

Reference 2 - 1.51% Coverage

Me and my sister and my niece and…you mean go and see where it comes from or where? No, not really. All of my older peoples that could tell us something where it came from, they don’t know. So it’s just me and my sisters here, and then I have a baby sister in North Carolina.

**Annotations**

1 Sub theme we were mentioning. More personal/emotional connection to ophthalmologist. Trust with ophthalmologist? Experience with specific ophthalmologist?
