# Supplementary material for: Interrogating a framework for diabetic retinopathy screening adherence: Qualitative insights from a severely affected and under-adherent population
Source: PLOS Glob Public Health. 2026 Apr 2;6(4):e0006230. doi: 10.1371/journal.pgph.0006230 (PMC13046135; doi:10.1371/journal.pgph.0006230)
Supplement: Attachment — Submitted filename: Response to Reviewers.docx [file pgph.0006230.s008.docx]

| Yale SCHOOL OF MEDICINE   *Department of Ophthalmology & Visual Science* |  | Yale Eye Center  40 Temple Street  Suite 1B  New Haven CT, 06510-2175 |
| --- | --- | --- |

**Editorial Office**

*PLOS Global Public Health*

January 26^th^, 2026

Dear Editors,

We thank both you and the reviewers for your thoughtful and detailed feedback, which has substantially strengthened the manuscript. Below, we address each comment point by point. All changes have been incorporated into the revised manuscript, with page and line numbers indicated where applicable.

**Reviewer 1:**

**Title**

*Comment 1:*

*The title could be re-framed as the phrase “qualitative interviews of a severe disease” seems inappropriate. Some suggestions: Interrogating a framework for diabetic retinopathy screening adherence: qualitative insights from a severely affected and unengaged population. Exploring screening adherence in severe diabetic retinopathy: a framework-based qualitative study.*

*Response:*

We agree that the phrase “qualitative interviews of a severe disease” is inappropriate and have revised the title of this manuscript accordingly to “Interrogating a framework for diabetic retinopathy screening adherence: qualitative insights from a severely affected and under-adherent population” (Title, Page 1, Lines 2-3).

**Introduction**

*Comment 1:*

*The paper could benefit with additional description on the context of DR in USA. Given the large number of immigrant communities who have made USA their home, providing information on which of these are most affected, whether DR is common across different ethnic groups, awareness about DR among these groups, issues concerning costs of seeking care, social support available to individuals to seek care, access to an easy availability of such care and influence of cultural beliefs and traditions would provide readers with a better grasp of the context in which the paper has been situated.*

*Response:*

We agree that providing more background on DR in the US is valuable to ensure readers understand the real-world setting this paper is situated in. In addition to the general barriers to DR care described previously in the second paragraph of the introduction, we have also added a paragraph to highlight how these challenges specifically affect racial and ethnic minority populations who are disproportionately affected by DR. We also incorporate a discussion on variations in awareness, social support, and cultural beliefs in these populations, emphasizing how these factors may influence engagement with eye care (Introduction, Page 3-4, Lines 82-89).

**Methods**

*Comment 1:*

*The small number of participants- 4 per group- is a cause for concern. With only 4 interviews per group, claiming thematic saturation may prove difficult. Do the authors feel confident that they have captured the full range of different experiences from just these 4 interviews?*

*Response:*

We thank the reviewer for highlighting the issue of sample size and thematic saturation. We agree that with only four participants per cohort, our findings cannot be considered exhaustive of all possible individual experiences within the two selected cohorts. Our aim, however, was not broad theory generation but rather to interrogate and refine an existing framework within two purposefully selected subgroups. We have revised the Methods section to clarify that we selected participants using purposive sampling to capture variation in engagement patterns and disease severity (Methods, Page 5, Lines 115-116).

Additionally, we have revised the Methods section to clarify that we achieved code saturation within these narrowly defined cohorts, rather than population-level thematic saturation (Methods, Page 7, Line 168). We have also revised the Study Limitations section to explicitly acknowledge that inclusion of more diverse participants from varied socioeconomic and cultural backgrounds could yield additional themes (Study Limitations, Page 20, Lines 595-597).

*Comment 2:*

*The definition of the two cohorts requires clarification. According to the authors the unengaged cohort were, “individuals with diabetes who had not received an eye exam in the past year”. I would think it should be framed as individual with diabetes who had “not sought an eye exam in the past year? After all, once informed about the importance of undergoing an eye check-up, the onus is on the patient to seek out this test? The severe disease cohort was defined as “individuals with DR who had undergone laser surgery or other procedures” Does this mean that severe disease cohort were regular in getting eye exams? Does it also mean that the unengaged cohort did not have severe disease and despite knowing that they had DR, were not seeking regular eye exams? Please clarify.*

*Response:*

We appreciate and agree with the reviewer’s request for clarification regarding cohort definitions. We have revised the Methods to more precisely define each group. The severe disease cohort is now explicitly described as individuals with clinically documented DR who had previously undergone laser treatment or other ocular procedures. We did not base this cohort assignment on engagement behavior, though a history of ocular interventions indicates some degree of prior interaction with eye care. However, this does not necessarily imply ongoing or regular engagement (Methods, Page 5, Lines 121-125). The unengaged cohort is now described as individuals with diabetes who had not sought or obtained an eye exam for the past year, regardless of the underlying reason. This does not imply that participants never sought eye care but rather reflects a lack of routine engagement consistent with screening guidelines but may still enable symptom-driven care. Therefore, we have renamed the “unengaged” cohort to the “under-adherent” cohort to signify that this cohort may still have obtained eye care, though not consistently. Additionally, we classified this cohort independently of DR status (Methods, Page 5, Lines 125-129).

*Comment 3:*

*The authors appear to have recruited participants from various sources, namely through the Yale Eye Center, through referrals from community partners and through word of mouth. It would be good to know, how many from each of these different sources responded to their call and fulfilled the eligibility criteria? Following this, how was the sampling of participants for the interviews actually done, were they all selected from different sources, what criteria were applied in the selection of these 8 participants? Ideally these participants, eg those in the severe disease group would need to be representing various aspects of engagement patterns. Similarly, these in the disengaged group should come from varied socio-economic and cultural backgrounds thereby bringing in diverse perceptions and experiences. Ensuring that the sample were maximally informative, combined with rich detailed interviews will strengthen the justification of the use of this small sample.*

*Response:*

We agree that greater transparency regarding recruitment and sampling strengthens methodological rigor. We have revised the Methods section to specify recruitment sources for the 8 interviewed participants: 4 were recruited through the Yale Eye Center and 4 through referrals from community partners (Methods, Page 5, Lines 118-119). We regret that we do not have recorded data on the total number of individuals contacted through each recruitment source or the number who met eligibility criteria, and we acknowledge this as a limitation.

Consistent with qualitative research practices, we selected participants using purposive sampling to capture variation in engagement patterns and disease severity rather than to achieve demographic representativeness, which we now specify in the revised Methods section (Methods, Page 5, Lines 115-116). Our intention was to recruit participants whose experiences would be maximally informative for exploring engagement and disengagement with care. In accordance with the reviewer’s comment, we have revised the Study Limitations section to explicitly note that inclusion of participants from more diverse socioeconomic and cultural backgrounds could yield additional themes and perspectives (Study Limitations, Page 20, Lines 595-597).

*Comment 4:*

*The authors also state that following introductions and documentation of demographic information the participants were shown a slide deck containing the framework previously developed by the research group. I believe that by so doing the authors may have compromised the inductive process which is so integral to qualitative research. While I agree, that the authors were seeking to validate the existing framework, they also sought to expand upon the framework. Presenting participants with the slide deck at the beginning of the interview, could have led participants to focus entirely on the existing framework with less focus on their own feelings and perceptions and in the process, perhaps, affecting the emergence of other issues!*

*Response:*

We thank the reviewer for this important methodological observation. Our intent in presenting the framework was to facilitate reflection and critique, as the study aim was to validate and expand upon a previously developed model. Even so, we agree it is important to consider that this approach may have introduced a degree of priming. We have revised the Methods section to clarify that despite presentation of the slide deck we still conducted interviews using open-ended prompts (Methods, Page 6, Lines 154-155). We have also added this issue as a limitation, acknowledging that early exposure to the framework may have shaped participants’ responses (Study Limitations, Page 21, Lines 624-625).

*Comment 5:*

*The author’s use of the grounded theory approach also needs justification. The authors need to explain why they decided on the use of this approach as against, say, a simple thematic analytical approach? Typically, Grounded theory is, inductive by design and best equipped to generate theory from data rather than test or validate a pre-existing framework. Having said this, GT can indeed be applied in validating frameworks, provided of course data are collected and coded inductively without forcing the framework. One could then see how (and to what extent) the categories identified from this analysis, aligns with the existing framework. But as participants were already exposed to the framework at the start of the study, the concern is that, perhaps, this inductive process may have been compromised which has implications for the comprehensiveness of the framework. I would have thought that a hybrid, deductive-inductive thematic analytical approach would have been more suitable.*

*Response:*

We appreciate the reviewer’s thoughtful critique of our analytic approach. We agree that traditional grounded theory is primarily inductive and oriented toward theory generation rather than framework validation. Our analytic strategy was therefore not classical grounded theory, but rather a hybrid deductive-inductive approach informed by grounded theory techniques, particularly constant comparison. Our original Methods section phrased this as “grounded theory and the constant comparative method to inductively and deductively develop themes”, which we agree is confusing and does not clearly explain the analytical methods used. Therefore, we have revised the Methods section to clarify this distinction and now describe our approach as “a hybrid deductive-inductive analytic approach informed by grounded theory techniques, particularly constant comparison,” in which we used a priori codes from the existing framework while remaining open to the emergence of new categories (Methods, Page 7, Lines 161-163).

*Comment 6:*

*The authors refer to having achieved thematic saturation when “no new codes emerged”. But given the small number of interviews, saturation may well have been achieved quite quickly, but the question is, would inclusion of other, perhaps more varied participants have contributed to inclusion of different codes, in which case can the authors really claim thematic saturation? Bernard (2012) recommended the inclusion of participants that ‘one would not normally consider and cautioned against the shaman effect, in that someone with specialized information on a topic can overshadow the data, whether intentionally or inadvertently’, (Bernard, R. H. (2012). Social research methods: Qualitative and quantitative approaches (2nd ed.). Thousand Oaks, CA: Sage).*

*Response:*

We agree with the reviewer that saturation in small samples must be interpreted cautiously. We have therefore revised our language in the Methods section to specify that we achieved within-sample code saturation rather than comprehensive thematic saturation across general populations (Methods, Page 7, Lines 168). We also explicitly acknowledge that inclusion of more diverse or atypical participants could have introduced additional codes in the Study Limitations section, consistent with study mentioned in the comment (Study Limitations, Page 20, Lines 595-597).

*Comment 7:*

*Equally important is the concept of triangulation which has a direct link with data saturation; data triangulation is a method to get to data saturation. The authors have not used any type of triangulation, ie data triangulation (getting similar information from more than one source), methods triangulations (use of multiple methods of data collection) both of which help build corroborative evidence thereby strengthening validity. Considering that the study aimed to validate the framework, getting inputs from multiple relevant stakeholders eg, close family members involved in the care of the patient with DR, ophthalmologists and other eye care health professionals etc could have further strengthened validity of the framework.*

*Response:*

We appreciate the reviewer’s emphasis on triangulation. We agree that methodological and data triangulation would have increased the validity of this study. We do believe we incorporated researcher triangulation in the analysis, with three independent coders including a trained community partner conducting the coding analysis. We now more explicitly define this in the Methods section (Methods, Page 7, Line 164-166). We also employed cohort-level triangulation by including two distinct participant groups with different disease severities and engagement patterns. We agree that including interviews of family members, ophthalmologists, and other healthcare professionals, as well as other methods of triangulation including recruiting participants from multiple institutions or geographical areas and involving multiple interviewers, would further strengthen validity. These points are now explicitly acknowledged as a limitation and are a key direction for future research (Study Limitations, Page 20, Lines 600-607).

*Comment 8:*

*The authors also need to describe what steps were taken to minimize researcher bias, given that all 8 interviews were carried out by one person. Use of researcher triangulation- more than one researcher doing the interviews - would have been of considerable help here. Additionally, the use of reflexivity as a process needs to be described, i.e whether the research team met after an interview to discuss how the interview went. As only few interviews were carried out, whether an interview was transcribed and reviewed by the whole team before moving on to the next? This would not only allow for more transparency but would enable a thorough review of the interview, guide the interviewer on how to be as open-ended as possible and identify gaps that could be addressed during the next interview. In this way one interview informs the next This method of ‘constant comparison’ a key characteristic of GT, is brought in not just at the data coding and analysis stage, but even earlier at the time of doing interviews.*

*Response:*

We agree that researcher reflexivity and interviewer effects warrant clearer reporting. We have revised the Methods section to describe the reflexive practices we used, including post-interview debriefing sessions mid-way through completing the interviews, iterative review of transcripts by the research team, and adjustment of subsequent interviews based on emerging insights, consistent with constant comparison (Methods, Page 7, Lines 166-168). As only one researcher conducted interviews, this limitation is now explicitly acknowledged in the Study Limitations section, and we emphasize that post-interview debriefing sessions, researcher triangulation in the analysis process, and involvement of a community partner in coding helped mitigate potential bias (Study Limitations, Page 20, Lines 600-603).

*Comment 9:*

*The use of two software platforms (NVivo and Dedoose) is redundant and not clearly justified, raising questions about data management consistency*

*Response:*

We thank the reviewer for raising this important point. We clarify that only NVivo was used for data management and analysis in the present study. The reference to Dedoose was an artifact from an earlier draft that included interviews from an additional cohort, which was subsequently removed from this manuscript. We apologize for the oversight. The Methods section has been corrected accordingly (Methods, Page 7, Lines 174).

**Results**

*Comment 1:*

*In Table 2 why is there number [8] placed against the head ‘Original Cohort’? A total of 24 interviews were done in the earlier study which is indicated below. The number 8 may be deleted to avoid confusion.*

*Response:*

We have deleted the number [8] in Table 2 to avoid confusion. This was added as a citation to the original paper (Table 2, page 8-9).

*Comment 2:*

*As a general rule please include qualifiers for every quote said by participants that has been included in the paper. For eg. this could include the age, gender, ethnicity and the specific cohort the respondent belonged to.*

*Response:*

We agree inclusion of these qualifiers for each quote are essential. We have included the participant number and specific cohort the respondent belonged to for each quote said by participants included in the paper. (Results, pages 10-16).

*Comment 3:*

*Vision Loss: Under this sub-theme the authors have reported that both cohorts reported that, “the need for new glasses motivated them to seek eye exams”. This implies that whether an individual belonged to the severe disease or unengaged cohort they had sought care when they felt they needed new glasses. How then can a cohort be described as being “unengaged”? how are they different from the severe disease cohort? For eg the following quote, from an individual belonging to the unengaged cohort states, “I could see a shadow coming around my eye. When, I got it, I called for Yale” Line No- 168-169. It is unclear who the unengaged really are, are these individuals who sought out eye care but did not receive it for whatever reason, or are they persons who did not at all seek care? In the case of the former, the problem then could lie with the health care set-up while in the latter, the onus is on the patient to seek out such care. A closer look at the data is called for to really flesh out what was unique to the two groups. In a similar kind of study that I had been involved in, complacency, poor appreciation of the serious harms of DR and about the importance of good control of blood sugars, the insidious course of the disease and others were some major reason that compromised care seeking among patients with DR. (Kumar S, Mohanraj R, Raman R, Kumar G, Luvies S, Machhi SS, et al. (2023) ‘I don‘t need an eye check-up’. A qualitative study using a behavioural model to understand treatment seeking behaviour of patients with sight threatening diabetic retinopathy (STDR) in India. PLoS ONE 18(6): e0270562. <https://doi.org/10.1371/journal.pone.0270562>*

*Response:*

We thank the reviewer for highlighting the need for greater conceptual clarity regarding the “unengaged” cohort. In our study, we defined the “unengaged” cohort, which we have renamed “under-adherent” as a response to this reviewer’s previous comment, as individuals with diabetes who had not sought or obtained an eye exam for the past year, regardless of the underlying reason. This does not imply that participants never sought eye care but rather reflects a lack of routine engagement consistent with screening guidelines but may still enable symptom-driven care. This definition is now more thoroughly explained in the Methods section (Methods, Page 5, Lines 125-129).

Although some under-adherent participants sought care when prompted by acute symptoms (ex. noticing a shadow in vision or needing new glasses), they did not maintain regular follow-up or adhere to recommended screening intervals. We have revised the Results section to explicitly distinguish between care-seeking in response to symptoms and guideline-based screening adherence, thereby clarifying what was unique to this under-adherent cohort (Results, Page 11, Lines 269-273).

*Comment 4:*

*Reading through the sub-theme on emotional context the respondent from the severe disease cohort said, “I hadn’t been to the eye doctor in over like seven years or more. I was just scared to go, I don’t know. I don’t know why. I guess I didn’t want to know what was going on with my eye”, while the respondent from the unengaged cohort said, “It kind of scares me a little bit because I don’t want to go blind. I want to see. I don’t want nobody leading me around, you know…that’s the wake-up for me too.” It appears that the respondent from the unengaged cohort was motivated to seek care while the one above from the severe disease cohort was too sacred to know. This is confusing!*

*Response:*

We agree that fear played different roles across participants and that our original presentation did not sufficiently articulate this distinction. Our findings suggest that fear can both inhibit and motivate care-seeking depending on context. In the severe disease cohort, fear was often described as a barrier earlier in the disease course but was later but was later transformed into sustained engagement through acceptance of their conditions and empowerment to seek care. In contrast, participants in the under-adherent cohort expressed fear of blindness but had not translated this concern into consistent screening engagement. We have revised the Results to clarify that emotional concern alone did not predict adherence and further explore this distinction observed in the two cohorts (Results, Page 12, Lines 305-309).

*Comment 5:*

*Line 197-199 “I feel right now at this moment my vision is stable, that it is controlled. I'm not afraid to be blind at this point. Honest to you. Like before, I was afraid to be blind”. This quote could have benefited with some explanation as to why the respondent did not fear becoming blind!*

*Response:*

We agree the context in which this participant is no longer afraid of becoming blind is crucial. We have revised that line to include an explanation as to what the quote is accomplishing: “Another participant expressed acceptance of their condition” (Results, Page 12, Line 302), and added an additional section to that quote to further contextualize why this participant does not fear becoming blind: “ ‘I don’t want that to happen, but if it happens, it happens’ “ (Results, Page 12, Lines 303-304). Namely, this participant has accepted their condition and the possible consequences of it and therefore does not fear becoming blind.

*Comment 6:*

*The sub-theme on patient doctor relationship has nicely brought out the needs of patients from the ‘severe disease cohort’ to feel cared which in turn fostered trust. What about issues concerning the comfort level of patients in asking questions, seeking information about their disease from the doctors? Did patients feel the need to seek out such information or preferred not to know more about the disease as this would only serve to accentuate their fear? I am also curious to understand why the authors state that the “unengaged cohort did not identify the patient-doctor relationship as a major factor in their eye care utilization”. What did these patients say that led the authors to come to this conclusion? The sections in the guide that elicits information on Resource Availability, Cues to Action, In-clinic Experience etc, I presume, would have also been asked of the unengaged cohort. In which case, the authors would do well to present issues that describe the nature of interaction the ‘unengaged’ may have had with the doctors earlier which may may have influenced any negative perceptions such patients may harbour towards the doctors? These are important issues that could better help understand ways of enhancing doctor-patients communications.*

*Response:*

Regarding the reviewer’s first point, we note that while our study did not explicitly probe participants’ comfort in asking questions or seeking information about their disease from their doctors, our findings suggest that positive patient-doctor relationships may facilitate such engagement. Participants in the severe disease cohort described feeling cared for and supported, which may encourage them to ask questions and better understand their condition. Conversely, under-adherent participants, who lacked sustained relationships with their doctors, may have had less opportunity or comfort to seek information. Future studies could explicitly explore patients’ comfort in obtaining information from their doctors to better understand how it interacts with adherence behaviors, which has been added to the Conclusions section (Conclusions, Page 21, Lines 633-635).

Regarding the reviewer’s second point, we appreciate request for greater transparency regarding how we concluded that patient-doctor relationships were not a theme influencing DR screening adherence in the under-adherent cohort. Participants in this cohort rarely discussed interpersonal relationships with physicians specifically as influencing their eye care utilization. Their accounts of care were largely regarding clinical staff as a whole, emphasizing logistical aspects of visits or the receipt of information rather than trust, emotional support, or continuity with a specific provider. No participant in this cohort described a sustained relationship with an eye care provider as a motivator for seeking or maintaining care. We have revised the Results to explicitly contrast this point between the severe disease cohort and the under-adherent cohort (Results, Page 15, Lines 414-419).

*Comment 7:*

*I feel it makes better sense to combine the sub-theme on ‘doctor-patient relationships’, and the sub-theme on ‘in-clinic experiences, as the latter, essentially describes the nature of their relationship with the nursing staff and others and how they were treated. Perhaps it could be labelled as Relationships/interactions with health care providers.*

*Response:*

We have revised the results section to include one theme, “In-Clinic Experiences” with a sub-theme of “Patient-Doctor Relationship” to better situation these related themes together (Results, Page 9, Line 204-211; Page 10, Line 234; Page 14-15, Lines 396-419). We have also revised Figure 1 to reflect this change.

*Comment 8:*

*Once again, reading the sub-themes on ‘Cues to action’ and ‘Knowledge creating experience’ the doubt about the definition of the two cohorts, “severe disease” and “unengaged”, remains. Here too the unengaged’ cohort are shown taking steps to “engage” with the health facility! Interestingly, a quote has been provided from an individual from the unengaged cohort who reported learning about DR from a primary doctor, “I had spoken to my primary care and then I read all the pamphlets that they give you” (Lines 273-274). There is thus, this confusion with using the label “unengaged cohort” as they do appear to have engaged with doctors. Needs explanation!*

*Response:*

We thank the reviewer for underscoring this important point regarding the definition of the under-adherent cohort. We have revised the manuscript to more clearly specify that this cohort consists of individuals with diabetes who did not receive an eye exam within the past year and were therefore under-adherent to diabetic retinopathy screening. Importantly, these individuals may still have engaged with the healthcare system in other ways during this period, such as through primary care, and may also have received eye exams prior to the 1-year timeframe (Methods, Page 5, Lines 125-129).

**Discussion**

*Comment 1:*

*Lines 295-296 in the discussion states, “When we asked participants where they learned about diabetic eye disease and screenings from, few cited the healthcare system”. This concept could be elaborated and made clearer. Do the authors mean that most patients do not get adequate information on DR from the health facility? Further, to complete this, could mention here other sources from where patients obtained information on DR, perhaps highlighting the more common sources.*

*Response:*

We agree this concept needs elaboration to increase clarity to the reader. We have revised this section of the discussion to specify that the “healthcare system” entails individual providers and clinic resources such as pamphlets and educational videos (Discussion, Page 17, Line 484-485). We have also included more common sources where patients obtained information on DR from including online resources and learning from the experiences of loved ones (Discussion, Page 17, Line 485-486).

*Comment 2:*

*Given that both groups spoke of lack of insurance and transportation as reasons that affected their care seeking, what then contributed to the severe disease group accessing care despite these barriers?*

*Response:*

We thank the reviewer for highlighting this important point. While both cohorts reported structural barriers such as lack of insurance and transportation, participants in the severe disease cohort were able to overcome these obstacles due to additional facilitators. Notably, long-term, trusting relationships with healthcare providers emerged as a key factor facilitating ongoing engagement in eye care. In contrast, participants in the under-adherent cohort lacked these interpersonal facilitators, illustrating how structural barriers may disproportionately inhibit care in the absence of strong patient-provider relationships. We have added this clarification to the Discussion section (Discussion, Page 18-19, Lines 525-538).

*Comment 3:*

*Lines 303-312 address issues concerning transportation and navigations costs. Lines 341 to 348 expand on this issue and may be best combined.*

*Response:*

We agree combining these lines will allow for the greatest impact of this point about resource barriers to the reader. We have revised the discussion section to combine these two sections (Discussion, Page 18, Lines 510-517).

*Comment 4:*

*Rather than say “sitting at eye level” (Line No. 335), perhaps a better phrase would be “sitting face to face”*

*Response:*

We agree that changing this phrasing more accurately reflect our point of improving communication through face-to-face contact. We have revised this line accordingly (Discussion, Page 19, Line 549).

*Comment 5:*

*The authors point out poor generalizability as a limitation of the study. But, qualitative studies in any case, cannot be generalized by virtue of the use of non-probability (Purposive sampling). Instead, I suggest, the authors talk about the concept of Transferability or the degree to which findings can be applied to other contexts. Typically, transferability can be enhanced through providing detailed, "thick" descriptions of the research context, participants, and methods, allowing others to assess if the findings are relevant to their own situations.*

*Response:*

We agree that a discussion of transferability is more valuable than generalizability in the context of this qualitative study. We have revised the Study Limitations section accordingly to reference “transferability” rather than “generalizability” and also included a description of how this is enhanced in our study through detailed descriptions of the research setting and participant cohorts (Study Limitations, Page 20, Lines 594-599).

*Comment 6:*

*The absence or both researcher and data triangulation is another limitation which the authors need to address. As the goal was to validate an existing framework, the authors need to clearly justify how a small sample of 8 participants (4 per group) and absence of other relevant stakeholders were deemed sufficient.*

*Response:*

We appreciate the reviewer’s concern regarding lack of triangulation and small sample size. First to address the small sample size and absence of other relevant stake holders, the purpose of our study was to validate and expand upon an existing DR screening adherence framework. As such, we intentionally selected a small, purposive sample of eight participants to capture specific cases that varied in disease severity and engagement patterns, which we now more explicitly outline in the Methods section (Methods, Page 5, Lines 115-116). Next, to address the issue of researcher and data triangulation, we did implement researcher triangulation in coding analysis with three researchers independently reviewing transcripts and collaboratively refining codes including a trained community partner, which we now more explicitly outline in the Methods section (Methods, Page 7, Lines 164-168). We acknowledge that data triangulation was limited, though the inclusion of two distinct participant cohorts allowing for comparison across experiences may partially limit this bias. We acknowledge that broader triangulation, including recruiting participants from multiple institutions or geographical areas, involving multiple interviewers, and interviewing additional stakeholders such as ophthalmologists, family members, or healthcare staff, would further strengthen validation. These clarifications have been added to the Study Limitations section (Study Limitations, Page 20, Lines 600-607).

*Comment 7:*

*Kindly refer to the COREQ (Consolidated criteria for reporting qualitative research) or the SRQR (Standards for reporting qualitative research).*

*Response:*

We have referenced the SPQR and ensured our study meets the standards outlined in this checklist.

**Reviewer 2:**

*Comment 1:*

*I appreciate the authors for conducting this study and enumerating all the factors associated with diabetic retinopathy screening adherence in detail. All the important factors have been explained thoroughly and these are very important for planning any program on Diabetic retinopathy screening. The limitations are a small sample size and participants from a single geographical area.*

*Response:*

We appreciate the reviewer’s time in assessing our paper and appreciate these comments.

**Reviewer 3:**

*Comment 1:*

*The authors must comply with the journal’s data-sharing policy by providing access to the analysis of transcripts and coding data to ensure transparency and reproducibility.*

Response:

We agree it is essential to share the transcripts and coding data. We have uploaded the interview transcripts with redacted HPI and coding stripes (S2 Appendix. Interview transcripts with coding stripes), NVivo node summaries with coded transcript excerpts by theme (S3 Appendix. Node summaries with coded transcript excerpts by theme), and the codebook with definitions (S4 Appendix. Qualitative codebook) as supplementary material to the journal submission website.

*Comment 2:*

*Address the limitations more thoroughly, including the small sample size and geographic scope, and discuss how future research could address these issues.*

Response:

We agree a further assessment of the limitations of this study is necessary. In line with the comments from Reviewer 1, the purpose of our study was to validate and expand upon an existing DR screening adherence framework. As such, we intentionally selected a small, purposive sample of eight participants to capture specific cases that varied in disease severity and engagement patterns (Methods, Page 5, Lines 115-116). To mitigate potential bias, we implemented implement researcher triangulation in coding analysis with three researchers independently reviewing transcripts and collaboratively refining codes including a trained community partner, which we now more explicitly outline in the Methods section (Methods, Page 7, Lines 164-166). While data triangulation was limited, the inclusion of two distinct participant cohorts allowed for comparison across experiences, partially addressing this concern. In terms of how future research could address these issues, we acknowledge potential solutions including addition of more diverse or atypical participant populations from varied socioeconomic and cultural backgrounds, recruiting participants from multiple institutions or geographical areas, involving multiple interviewers, and incorporating perspectives of family members, ophthalmologists, and other healthcare professionals. We have revised the Study Limitations section to incorporate these suggestions (Study Limitations, Page 20, Lines 604-607).

*Comment 3:*

*Clarify the potential impact of researcher bias and social desirability bias on the findings.*

*Response:*

We thank the reviewer for raising this important point. Researcher bias may have influenced findings through framing of questions or interactions with participants, particularly as a single researcher conducted all interviews. While we partially mitigated researcher bias through researcher triangulation and involvement of a community partner in coding analysis, it is possible that participants’ responses were shaped by the researcher’s presence or the framing of prompts. Similarly, social desirability bias may have led participants to over-report positive attitudes toward care, especially when discussing sensitive topics such as fear of blindness or relationships with healthcare providers. We acknowledge that these biases could influence the reporting and interpretation of themes, particularly around patient-doctor relationships. We have clarified these limitations in the revised manuscript (Study Limitations, Pages 20-21, Lines 608-629).
