# Supplementary material for: Interrogating a framework for diabetic retinopathy screening adherence: Qualitative insights from a severely affected and under-adherent population
Source: PLOS Glob Public Health. 2026 Apr 2;6(4):e0006230. doi: 10.1371/journal.pgph.0006230 (PMC13046135; doi:10.1371/journal.pgph.0006230)
Supplement: Attachment — Submitted filename: Response_to_Reviewers_auresp_2.docx [file pgph.0006230.s009.docx]

| Yale SCHOOL OF MEDICINE   *Department of Ophthalmology & Visual Science* |  | Yale Eye Center  40 Temple Street  Suite 1B  New Haven CT, 06510-2175 |
| --- | --- | --- |

**Editorial Office**

*PLOS Global Public Health*

February 25^th^, 2026

Dear Editors,

We thank both you and the reviewers for your thoughtful and detailed feedback, which has substantially strengthened the manuscript. Below, we address each comment point by point. All changes have been incorporated into the revised manuscript, with page and line numbers indicated where applicable.

**Reviewer 1:**

*Comment 1:*

*The definitions of the two cohorts continue to be confusing. From what I understand the recruitment of the two groups are based on different criteria:*

*Severe disease cohort -defined by clinical status (DR severity and prior treatment)*

*Under-adherent cohort - defined by care-seeking behaviour (recent engagement).*

*Thus, a patient could be severe and under-adherent, or mild and adherent, which begs the question whether these are really comparable cohorts, or just different types of study participants. I believe that it would be methodologically sound to frame this as maximum variation sampling, rather than as two discrete groups. By doing this you avoid the implication that these are two different analytic categories, but is a sampling dimension. Further it aligns with qualitative aims of capturing diverse perceptions from different kinds of patients. The authors could state that maximum variation sampling was used to capture a wide range of experiences related to diabetic eye care, including severity of diabetic retinopathy and patterns of engagement with eye care services.*

*Response:*

We appreciate the reviewer’s suggestion, which has strengthened the manuscript substantially. We agree that the original framing can imply that these were distinct comparison groups. Therefore, we have reframed the purpose of our manuscript to validate and expand upon our previously developed framework by examining barriers and facilitators across two patient populations rather than compare two discrete cohorts (Lines 25-27, Lines 79-81, Lines 430-432). We have also revised the Methods section to explicitly describe our approach as maximum variation sampling, with participants purposively selected to capture heterogeneity in both disease severity and engagement with eye care services (Methods, Page 5, Lines 94-96). We have also revised the manuscript throughout to remove comparative language and avoid positioning participants as distinct cohorts. In addition, we have consolidated the two prior framework figures into a single figure presenting the Enhanced Diabetic Retinopathy Screening Adherence Framework, integrating themes identified across all participants (Figure 1).

*Comment 2:*

*Authors should could also clarify that when they use the term “severe disease” whether it pertains to severe form of diabetes or severe form of DR.*

*Response:*

We agree that this clarification is important and thank the reviewer for highlighting this concern. We have revised the manuscript throughout to refer to this participant population as “severe diabetic retinopathy (DR)” rather than “severe disease” to avoid confusion.

*Comment 3:*

*Line No. 70 -72: Social and cultural factors, such as self-reliance, cultural competency of providers, and beliefs about health and illness can further influence whether individuals seek care. This sentence could be elaborated on to make issues clear. Typically, self -reliance is seen as a positive quality indicating independence. Given this, what is actually meant here? Do the authors mean that people believe they can manage their eye issues themselves and do not need to see an eye doctor? Also, what is implied by “cultural competency” of providers?*

*Response:*

We thank the reviewer for this thoughtful comment. We agree that the terms “self-reliance” and “cultural competency” could be interpreted in multiple ways and should be clarified. We refer to “self-reliance” as cultural norms or beliefs that emphasize managing health concerns independently, which may lead some individuals to delay or forgo professional eye care in favor of managing problems on their own. We did not intend to frame self-reliance as inherently negative but rather highlight how it may influence care-seeking behavior. In terms of the phrase “cultural competency of providers,” we refer to US providers’ ability to effectively communicate with and understand patients from diverse cultural backgrounds, such as being aware of culturally shaped health beliefs. Limited cultural competency or experience in caring for individuals from diverse backgrounds may contribute to misunderstandings, reduced trust, and lower adherence to recommended diabetic retinopathy screening. We have revised the Introduction to clarify and elaborate on these two terms (Introduction, Pages 3-4, Lines 67-72).

*Comment 4:*

*Line No. 213 -214: Rather, this engagement may be more reactive and therefore intermittent, occurring only when vision changes became subjectively noticeable. Could perhaps write in the past sentence and state, “Rather, this engagement was more reactive and therefore intermittent…”*

*Response:*

We agree that writing this sentence in the past-tense is more appropriate and have revised the manuscript accordingly (Results, Page 11, Line 200).

*Comment 5:*

*Line no. 232-234: “It kind of scares me a little bit because I don’t want to go blind. I want to see. I don’t want nobody leading me around, you know…that’s the wake-up for me too.” (Participant 4, Under-Adherent Cohort). Despite this fear of going blind why then was no care sought? The authors too have indicated in lines 245-246 that fear of blindness did not consistently translate into routine screening. Would be interesting to understand why.*

*Response:*

We thank the reviewer for raising this important point and agree that it warrants clearer discussion in the manuscript. The participant described insurance-related challenges that required out-of-pocket payment, as well as caregiving responsibilities for a sick family member, which limited their ability to seek care. These findings highlight that even when individuals are motivated to obtain eye examinations, whether due to fear of blindness or other concerns, structural and contextual barriers may still prevent engagement in eye care. Such barriers can include competing priorities, limited resources, and gaps in understanding about recommended care. We have now explicitly clarified this point in the Results section (Results, Page 12, Lines 228-232).

*Comment 6:*

*The piece on housing insecurity affecting access to care needs some clarification. Aside from stating that it was “difficult” it is unclear what about being homeless contributed to difficulties in accessing care? I can understand that homelessness would have made it difficult for the individual to maintain good eye health, eye hygiene, being regular in taking medication/eye drops etc but how did it affect “access to care”?*

*Response:*

We thank the reviewer for this thoughtful comment and agree that additional clarification is warranted. Upon revisiting the original interview transcript (S2 Appendix), the participant did not elaborate further on how homelessness specifically affected their access to care beyond describing it as “difficult.”

Given the limits of the available data, we are cautious not to overinterpret the participant’s experience. However, housing insecurity is well documented in the literature as a structural barrier to healthcare access due to factors such as lack of a primary care provider, low health literacy, lack of insurance and financial constraints, and lack of transportation. We have revised the Results section to reflect that while the participant did not provide specific details, housing instability likely created logistical and structural challenges that affected their ability to consistently engage in eye care (Results, Page 13, Lines 253-255).

*Comment 5:*

*Finally, the results clearly show that across the two groups there were no unique characteristics or differences, except of course the doctor-patient relationship, which again makes one question the relevance of dividing the participants, it actually undermines the rationale for treating them as distinct groups, but does not undermine the study. In fact, handled well, it can strengthen your argument.*

*Response:*

We thank the reviewer for this thoughtful and constructive observation. We agree that the substantial thematic overlap across participants is notable and we believe this consistency strengthens the argument that the identified barriers and facilitators operate across varying levels of disease severity and engagement with care. In alignment with our response to Comment 1, we have reframed the manuscript to emphasize framework validation and refinement rather than comparison between groups. The study is now positioned as applying the previously developed DR screening adherence framework across two participant populations using a maximum variation sampling strategy (Lines 25-27, Lines 79-81, Lines 430-432). The recurrence of core themes across participants supports the robustness and transferability of the framework across the disease spectrum.

At the same time, the emergence of the patient-doctor relationship as a salient sub-theme within the broader domain of in-clinic experiences further refines the framework by highlighting interpersonal relationships and trust as important facilitators of sustained engagement. We have revised the Results and Discussion sections and Figure 1 accordingly (Figure 1; Lines 38-40, Lines 160-163, Lines 284-286, Lines 321-323, Lines 368-376).

**Reviewer 2:**

*Comment 1:*

*All the reviewer’s comments have been addressed.*

*Response:*

We thank the reviewer for their time in reviewing our manuscript and greatly appreciate their thoughts and suggestions.

**Reviewer 3:**

*Comment 1:*

*(No Response)*

*Response:*

We thank the reviewer for their time in reviewing our manuscript and greatly appreciate their thoughts and suggestions.
