# Supplementary material for: Interrogating a framework for diabetic retinopathy screening adherence: Qualitative insights from a severely affected and under-adherent population
Source: PLOS Glob Public Health. 2026 Apr 2;6(4):e0006230. doi: 10.1371/journal.pgph.0006230 (PMC13046135; doi:10.1371/journal.pgph.0006230)
Supplement: Attachment — Submitted filename: Reviewer Comments- PLOS Global Public Health.docx [file pgph.0006230.s005.docx]

**PLOS Global Public Health**

**Interrogating a framework for diabetic retinopathy screening adherence:** **qualitative interviews of a severe disease and unengaged population**

**Reviewer Comments**

This is an interesting and well written paper on validating a framework for DR screening and adherence. While the use of qualitative research methods is appropriate, there are several methodological issues that have not been clearly addressed. These are described below:

**Title**

The title could be re-framed as the phrase “qualitative interviews of a severe disease” seems inappropriate. Some Suggestions:

- Interrogating a framework for diabetic retinopathy screening adherence: qualitative insights from a severely affected and unengaged population
- Exploring screening adherence in severe diabetic retinopathy: a framework-based qualitative study

**Introduction**

- The paper could benefit with additional description on the context of DR in USA. Given the large number of immigrant communities who have made USA their home, providing information on which of these are most affected, whether DR is common across different ethnic groups, awareness about DR among these groups, issues concerning costs of seeking care, social support available to individuals to seek care, access to an easy availability of such care and influence of cultural beliefs and traditions would provide readers with a better grasp of the context in which the paper has been situated

**Methods**

- The small number of participants- 4 per group- is a cause for concern. With only 4 interviews per group, claiming thematic saturation may prove difficult. Do the authors feel confident that they have captured the full range of different experiences from just these 4 interviews?
- The definition of the two cohorts requires clarification. According to the authors the unengaged cohort were, *“individuals with diabetes who* ***had not received an eye exam*** *in the past year”.* I would think it should be framed as individual with diabetes who had **“not sought an eye exam** in the past year? After all, once informed about the importance of undergoing an eye check-up, the onus is on the patient to seek out this test? The severe disease cohort was defined as *“individuals with DR who had undergone laser surgery or other procedures”* Does this mean that severe disease cohort were regular in getting eye exams? Does it also mean that the unengaged cohort did not have severe disease and despite knowing that they had DR, were **not seeking** regular eye exams? Please clarify.
- The authors appear to have recruited participants from various sources, namely through the Yale Eye Center, through referrals from community partners and through word of mouth. It would be good to know, how many from each of these different sources responded to their call and fulfilled the eligibility criteria? Following this, how was the sampling of participants for the interviews actually done, were they all selected from different sources, what criteria were applied in the selection of these 8 participants? Ideally these participants, eg those in the severe disease group would need to be representing various aspects of engagement patterns. Similarly, these in the disengaged group should come from varied socio-economic and cultural backgrounds thereby bringing in diverse perceptions and experiences. Ensuring that the sample were maximally informative, combined with rich detailed interviews will strengthen the justification of the use of this small sample.
- The authors also state that following introductions and documentation of demographic information the participants were shown a slide deck containing the framework previously developed by the research group. I believe that by so doing the authors may have compromised the inductive process which is so integral to qualitative research. While I agree, that the authors were seeking to validate the existing framework, they also sought to expand upon the framework. Presenting participants with the slide deck at the beginning of the interview, could have led participants to focus entirely on the existing framework with less focus on their own feelings and perceptions and in the process, perhaps, affecting the emergence of other issues!
- The author’s use of the grounded theory approach also needs justification. The authors need to explain why they decided on the use of this approach as against, say, a simple thematic analytical approach? Typically, Grounded theory is, inductive by design and best equipped to generate theory from data rather than test or validate a pre-existing framework. Having said this, GT can indeed be applied in validating frameworks, provided of course data are collected and coded inductively without forcing the framework. One could then see how (and to what extent) the categories identified from this analysis, aligns with the existing framework. But as participants were already exposed to the framework at the start of the study, the concern is that, perhaps, this inductive process may have been compromised which has implications for the comprehensiveness of the framework. I would have thought that a hybrid, deductive-inductive thematic analytical approach would have been more suitable.
- The authors refer to having achieved thematic saturation when “no new codes emerged”. But given the small number of interviews, saturation may well have been achieved quite quickly, but the question is, would inclusion of other, perhaps more varied participants have contributed to inclusion of different codes, in which case can the authors really claim thematic saturation? Bernard (2012) recommended the inclusion of participants that ‘one would not normally consider and cautioned against the *shaman effect*, in that someone with specialized information on a topic can overshadow the data, whether intentionally or inadvertently’, (Bernard, R. H. (2012). *Social research methods: Qualitative and quantitative approaches* (2nd ed.). Thousand Oaks, CA: Sage).
- Equally important is the concept of triangulation which has a direct link with data saturation; data triangulation is a method to get to data saturation. The authors have not used any type of triangulation, ie data triangulation (getting similar information from more than one source), methods triangulations (use of multiple methods of data collection) both of which help build corroborative evidence thereby strengthening validity. Considering that the study aimed to validate the framework, getting inputs from multiple relevant stakeholders eg, close family members involved in the care of the patient with DR, ophthalmologists and other eye care health professionals etc could have further strengthened validity of the framework.
- The authors also need to describe what steps were taken to minimize researcher bias, given that all 8 interviews were carried out by one person. Use of researcher triangulation- more than one researcher doing the interviews - would have been of considerable help here. Additionally, the use of reflexivity as a process needs to be described, i.e whether the research team met after an interview to discuss how the interview went. As only few interviews were carried out, whether an interview was transcribed and reviewed by the whole team before moving on to the next? This would not only allow for more transparency but would enable a thorough review of the interview, guide the interviewer on how to be as open-ended as possible and identify gaps that could be addressed during the next interview. In this way one interview informs the next This method of ‘constant comparison’ a key characteristic of GT, is brought in not just at the data coding and analysis stage, but even earlier at the time of doing interviews.
- The use of two software platforms (NVivo and Dedoose) is redundant and not clearly justified, raising questions about data management consistency

**Results**

- In Table 2 why is there number [8] placed against the head ‘Original Cohort’? A total of 24 interviews were done in the earlier study which is indicated below. The number 8 may be deleted to avoid confusion.
- As a general rule please include qualifiers for every quote said by participants that has been included in the paper. For eg. this could include the age, gender, ethnicity and the specific cohort the respondent belonged to.
- Vision Loss: Under this sub-theme the authors have reported that both cohorts reported that***,*** *“the need for new glasses motivated them to seek eye exams”*. This implies that whether an individual belonged to the severe disease or unengaged cohort they had sought care when they felt they needed new glasses. How then can a cohort be described as being “unengaged”? how are they different from the severe disease cohort? For eg the following quote, from an individual belonging to the unengaged cohort states, “*I could see a shadow coming around my eye. When, I got it, I called for Yale”* Line No- 168-169. It is unclear who the unengaged really are, are these individuals who sought out eye care but did not receive it for whatever reason, or are they persons who did not at all seek care? In the case of the former, the problem then could lie with the health care set-up while in the latter, the onus is on the patient to seek out such care. A closer look at the data is called for to really flesh out what was unique to the two groups. In a similar kind of study that I had been involved in, complacency, poor appreciation of the serious harms of DR and about the importance of good control of blood sugars, the insidious course of the disease and others were some major reason that compromised care seeking among patients with DR. (Kumar S, Mohanraj R, Raman R, Kumar G, Luvies S, Machhi SS, et al. (2023) ‘I don‘t need an eye check-up’. A qualitative study using a behavioural model to understand treatment seeking behaviour of patients with sight threatening diabetic retinopathy (STDR) in India. PLoS ONE 18(6): e0270562. <https://doi.org/10.1371/journal.pone.0270562>
- Reading through the sub-theme on emotional context the respondent from the severe disease cohort said, *“I hadn’t been to the eye doctor in over like seven years or more. I was just scared to go, I don’t know. I don’t know why. I guess I didn’t want to know what was going on with my eye”,* while the respondent from the unengaged cohort said, “*It kind of scares me a little bit because I don’t want to go blind. I want to see. I don’t want nobody leading me around, you know…that’s the wake-up for me too.”* It appears that the respondent from the unengaged cohort was motivated to seek care while the one above from the severe disease cohort was too sacred to know. This is confusing!
- Line 197-199 “*I feel right now at this moment my vision is stable, that it is controlled. I'm not afraid to be blind at this point. Honest to you. Like before, I was afraid to be blind”.* This quote could have benefited with some explanation as to why the respondent did not fear becoming blind!
- The sub-theme on patient doctor relationship has nicely brought out the needs of patients from the ‘severe disease cohort’ to feel cared which in turn fostered trust. What about issues concerning the comfort level of patients in asking questions, seeking information about their disease from the doctors? Did patients feel the need to seek out such information or preferred not to know more about the disease as this would only serve to accentuate their fear? I am also curious to understand why the authors state that the *“unengaged cohort did not identify the patient-doctor relationship as a major factor in their eye care utilization”.* What did these patients say that led the authors to come to this conclusion? The sections in the guide that elicits information on Resource Availability, Cues to Action, In-clinic Experience etc, I presume, would have also been asked of the unengaged cohort. In which case, the authors would do well to present issues that describe the nature of interaction the ‘unengaged’ may have had with the doctors earlier which may may have influenced any negative perceptions such patients may harbour towards the doctors? These are important issues that could better help understand ways of enhancing doctor-patients communications.
- I feel it makes better sense to combine the sub-theme on ‘doctor-patient relationships’, and the sub-theme on ‘in-clinic experiences, as the latter, essentially describes the nature of their relationship with the nursing staff and others and how they were treated. Perhaps it could be labelled as Relationships/interactions with health care providers
- Once again, reading the sub-themes on ‘Cues to action’ and ‘Knowledge creating experience’ the doubt about the definition of the two cohorts, “severe disease” and “unengaged”, remains. Here too the unengaged’ cohort are shown taking steps to “engage” with the health facility! Interestingly, a quote has been provided from an individual from the unengaged cohort who reported learning about DR from a primary doctor, *“I had spoken to my primary care and then I read all the pamphlets that they give you”* (Lines 273-274*).* There is thus, this confusion with using the label “unengaged cohort” as they do appear to have engaged with doctors. Needs explanation!

**Discussion**

- Lines 295-296 in the discussion states, *“When we asked participants where they learned about diabetic eye disease and screenings from, few cited the healthcare system”*. This concept could be elaborated and made clearer. Do the authors mean that most patients do not get adequate information on DR from the health facility? Further, to complete this, could mention here other sources from where patients obtained information on DR, perhaps highlighting the more common sources.

- Given that both groups spoke of lack of insurance and transportation as reasons that affected their care seeking, what then contributed to the severe disease group accessing care despite these barriers?
- Lines 303-312 address issues concerning transportation and navigations costs. Lines 341 to 348 expand on this issue and may be best combined
- Rather than say “*sitting at eye level*” (Line No. 335), perhaps a better phrase would be “sitting face to face”
- The authors point out poor generalizability as a limitation of the study. But, qualitative studies in any case, cannot be generalized by virtue of the use of non-probability (Purposive sampling). Instead, I suggest, the authors talk about the concept of Transferability or the degree to which findings can be applied to other contexts. Typically, transferability can be enhanced through providing detailed, "thick" descriptions of the research context, participants, and methods, allowing others to assess if the findings are relevant to their own situations.
- The absence or both researcher and data triangulation is another limitation which the authors need to address. As the goal was to validate an existing framework, the authors need to clearly justify how a small sample of 8 participants (4 per group) and absence of other relevant stakeholders were deemed sufficient.
- Kindly refer to the COREQ (Consolidated criteria for reporting qualitative research) or the SRQR (Standards for reporting qualitative research) .
