# Supplementary material for: Interrogating a framework for diabetic retinopathy screening adherence: Qualitative insights from a severely affected and under-adherent population
Source: PLOS Glob Public Health. 2026 Apr 2;6(4):e0006230. doi: 10.1371/journal.pgph.0006230 (PMC13046135; doi:10.1371/journal.pgph.0006230)
Supplement: Attachment — Submitted filename: Reviewer Comments.docx [file pgph.0006230.s007.docx]

**Interrogating a framework for diabetic retinopathy** **screening adherence: qualitative insights from a** **severely affected** **and under-adherent population**

The authors have worked hard to produce a much-improved draft and while most concerns raised earlier have been adequately addressed, there are a few issues that need to be examined.

- The definitions of the two cohorts continue to be confusing. From what I understand the recruitment of the two groups are based on different criteria:

Severe disease cohort -defined by clinical status (DR severity and prior treatment)

Under-adherent cohort - defined by care-seeking behaviour (recent engagement).

- Thus, a patient could be severe and under-adherent, or mild and adherent, which begs the question whether these are really comparable cohorts, or just different types of study participants. I believe that it would be methodologically sound to frame this as maximum variation sampling, rather than as two discrete groups. By doing this you avoid the implication that these are two different analytic categories, but is a sampling dimension. Further it aligns with qualitative aims of capturing diverse perceptions from different kinds of patients. The authors could state that maximum variation sampling was used to capture a wide range of experiences related to diabetic eye care, including severity of diabetic retinopathy and patterns of engagement with eye care services.
- Authors should could also clarify that when they use the term “severe disease” whether it pertains to severe form of diabetes or severe form of DR.
- Line No. 70 -72: *Social and cultural factors, such as self-reliance, cultural competency of providers, and beliefs about health and illness can further influence whether individuals seek care.* This sentence could be elaborated on to make issues clear. Typically, self -reliance is seen as a positive quality indicating independence. Given this, what is actually meant here? Do the authors mean that people believe they can manage their eye issues themselves and do not need to see an eye doctor? Also, what is implied by “cultural competency” of providers?
- Line No. 213 -214: *Rather, this engagement may be more reactive and therefore intermittent, occurring only when vision changes became subjectively noticeable.* Could perhaps write in the past sentence and state, “Rather, this engagement **was more reactive** and therefore intermittent…”
- Line no. 232-234: “*It kind of scares me a little bit because I don’t want to go blind. I want to see. I don’t want nobody leading me around, you know…that’s the wake-up for me too.”* (Participant 4, Under-Adherent Cohort). Despite this fear of going blind why then was no care sought? The authors too have indicated in lines 245-246 that fear of blindness did not consistently translate into routine screening. Would be interesting to understand why.
- The piece on housing insecurity affecting access to care needs some clarification. Aside from stating that it was *“difficult”* it is unclear what about being homeless contributed to difficulties in accessing care? I can understand that homelessness would have made it difficult for the individual to maintain good eye health, eye hygiene, being regular in taking medication/eye drops etc but how did it affect “access to care”?
- Finally, the results clearly show that across the two groups there were no unique characteristics or differences, except of course the doctor-patient relationship, which again makes one question the relevance of dividing the participants, it actually **undermines the rationale for treating them as distinct groups,** but does not undermine the study. In fact, handled well, it can **strengthen your argument.**
